# Drugs that reverse disease transcriptomic signatures are more effective in a mouse model of dyslipidemia

**DOI:** 10.15252/msb.20145486

**Published:** 2015-03-03

**Authors:** Allon Wagner, Noa Cohen, Thomas Kelder, Uri Amit, Elad Liebman, David M Steinberg, Marijana Radonjic, Eytan Ruppin

**Affiliations:** 1 The Blavatnik School of Computer Science, Tel Aviv UniversityTel Aviv, Israel; 2 Department of Electrical Engineering and Computer Science, University of CaliforniaBerkeley, CA, USA; 3 Microbiology and Systems Biology, TNOZeist, the Netherlands; 4 Neufeld Cardiac Research Institute, Tel Aviv UniversityTel Aviv, Israel; 5 Regenerative Medicine Stem Cells and Tissue Engineering Center, Sheba Medical CenterTel Hashomer, Israel; 6 Department of Computer Science, University of Texas at AustinAustin, TX, USA; 7 Department of Statistics and Operations Research, Tel Aviv UniversityTel Aviv, Israel; 8 The Sackler School of Medicine, Tel Aviv UniversityTel Aviv, Israel; 9 Department of Computer Science, Institute of Advanced Computer Sciences (UMIACS) & the Center for Bioinformatics and Computational Biology, University of MarylandCollege Park, MD, USA

**Keywords:** connectivity map, disease reversal, drug repositioning, homeostasis, systems medicine

## Abstract

High-throughput omics have proven invaluable in studying human disease, and yet day-to-day clinical practice still relies on physiological, non-omic markers. The metabolic syndrome, for example, is diagnosed and monitored by blood and urine indices such as blood cholesterol levels. Nevertheless, the association between the molecular and the physiological manifestations of the disease, especially in response to treatment, has not been investigated in a systematic manner. To this end, we studied a mouse model of diet-induced dyslipidemia and atherosclerosis that was subject to various drug treatments relevant to the disease in question. Both physiological data and gene expression data (from the liver and white adipose) were analyzed and compared. We find that treatments that restore gene expression patterns to their norm are associated with the successful restoration of physiological markers to their baselines. This holds in a tissue-specific manner—treatments that reverse the transcriptomic signatures of the disease in a particular tissue are associated with positive physiological effects in that tissue. Further, treatments that introduce large non-restorative gene expression alterations are associated with unfavorable physiological outcomes. These results provide a sound basis to *in silico* methods that rely on omic metrics for drug repurposing and drug discovery by searching for compounds that reverse a disease's omic signatures. Moreover, they highlight the need to develop drugs that restore the global cellular state to its healthy norm rather than rectify particular disease phenotypes.

## Introduction

High-throughput gene expression data serve to illuminate the molecular underpinnings of different cellular phenotypes. Such data have proven indispensable to studying the molecular basis of human disease (Cooper-Knock *et al*, 2012). They were further used to establish risk biomarkers (Scherzer *et al*, 2007), to predict prognosis and clinical outcome (Sørlie *et al*, 2001; Pomeroy *et al*, 2002; Singh *et al*, 2002; Sotiriou *et al*, 2003; Willenbrock *et al*, 2004; Chuang *et al*, 2007, 2012; Oh *et al*, 2012), and to examine the effects of drug treatment (Cheok *et al*, 2003; Baur *et al*, 2006; Fernald & Altman, 2013). Assays that examine gene expression have already been put to clinical use (McCarthy *et al*, 2013) and are expected to become a principal element of future personalized medical care.

Yet, present medical diagnosis and decisions concerning clinical interventions rely extensively on measurement of relevant physiological markers such as blood and urine indices that can be readily obtained from patients (Hood & Price, 2014). This is particularly true in the case of the metabolic syndrome and related disorders (Mattix *et al*, 2002; Huang, 2009; American Diabetes Association, 2013; Imran *et al*, 2013). A deviation of the physiological markers from their normal levels assists in establishing a diagnosis and indicates elevated disease-associated risk. For example, higher than normal levels of blood glucose and blood triglycerides form two of the criteria that define the metabolic syndrome (Huang, 2009), and are associated with elevated cardiovascular risk. By the same token, clinical interventions are expected to restore these markers to their normal levels, and such a restoration is construed as a sign of reduced disease-associated risk (Qaseem *et al*, 2007; Moghissi *et al*, 2009; American Diabetes Association, 2013; Harper *et al*, 2013).

Thus, the effectiveness of extant treatments is judged not only by their ability to reverse the clinical course of the disease, but also by their ability to restore the patients’ physiological markers to their norm. One might assume that the latter is also indicative of a restoration of disease-affected *molecular-level* phenotypes to their norm. In other words, it is natural to assume that effective clinical treatments serve, in parallel, to eliminate disease-induced abnormalities both at the organismic level and at the cellular level. Notably, several recent studies have made this implicit assumption by directly searching for drug therapies whose molecular effects anti-correlate with disease molecular signatures (Hu & Agarwal, 2009; Boyle *et al*, 2010; Chang *et al*, 2010; Josset *et al*, 2010; Dudley *et al*, 2011; Kunkel *et al*, 2011; Sirota *et al*, 2011; Qu & Rajpal, 2012; Jahchan *et al*, 2013; Pacini *et al*, 2013; Yizhak *et al*, 2013; Zerbini *et al*, 2014). The underlying logic is clear: A treatment that eliminates molecular-level abnormalities is assumed to do the same at the physiological level. These studies have already shown considerable success and offer an appealing way to search for novel drug targets, or to repurpose existing drugs rationally. However, to the best of our knowledge, the fundamental relationship between the effects of pharmacological interventions at the physiological, organismic level and their effects at the molecular level has not been rigorously studied up to date.

Here, we address this challenge and ask whether extant treatments that restore physiological indices back to their baseline levels also reverse disease phenotypes at the molecular level. This questions is studied in one case study, a mouse model of dyslipidemia for which pertaining physiological and molecular data have been collected (Radonjic *et al*, 2013). This model is particularly apt to study the questions at hand because diagnosis and clinical risk assessment in the case of the metabolic syndrome and related disorders depend almost exclusively on physiological markers (Huang, 2009).

## Results

### Design and methodology

We analyze hepatic and white adipose gene expression, as well as 26 disease-relevant physiological markers, measured in a LDLR^−/−^ mouse model of diet-induced dyslipidemia (Supplementary Table S1). Low-density lipoprotein receptor (LDLR)-deficient mice are genetically predisposed to develop hypercholesterolemia and atherosclerotic lesions. These manifestations appear in mild forms even under a normal diet, but are greatly aggravated when the mice are placed on a high-fat diet (Zadelaar *et al*, 2007; Getz & Reardon, 2012; Ma *et al*, 2012). The experimental setting was previously described (Radonjic *et al*, 2013); it is summarized here and in Box  1A for completeness’ sake.

**Box 1: Experimental design and methodology**

(A) The experimental design. Physiological markers and hepatic gene expression were measured in all animals. Adipose gene expression was measured in the experimental groups marked with a white outline. The colors that mark each experimental group are retained throughout the figures in the paper. (B) A schematic illustration of the main research question. Left panel: shows the *gene expression space*, onto which the gene expression of each subject is projected (Materials and Methods). Blue, red, and green markers represent LFD, untreated HFD, and treated HFD subjects, respectively. The transcriptome deviation index (TDI) of a subject (dashed arrows) is the distance of its gene expression from the mean gene expression of the LFD group (dotted yellow triangle), which represents the healthy baseline. Right panel: shows the physiological space. Organism-level physiological markers are collected from the same subjects and projected onto a *physiological space* (Materials and Methods). The *global physiological deviation index* (GPDI) (dashed arrows) is the distance of a sample from the mean of the LFD group in the physiological space. The main research question of the current study translates into asking whether TDIs and GPDIs are correlated.

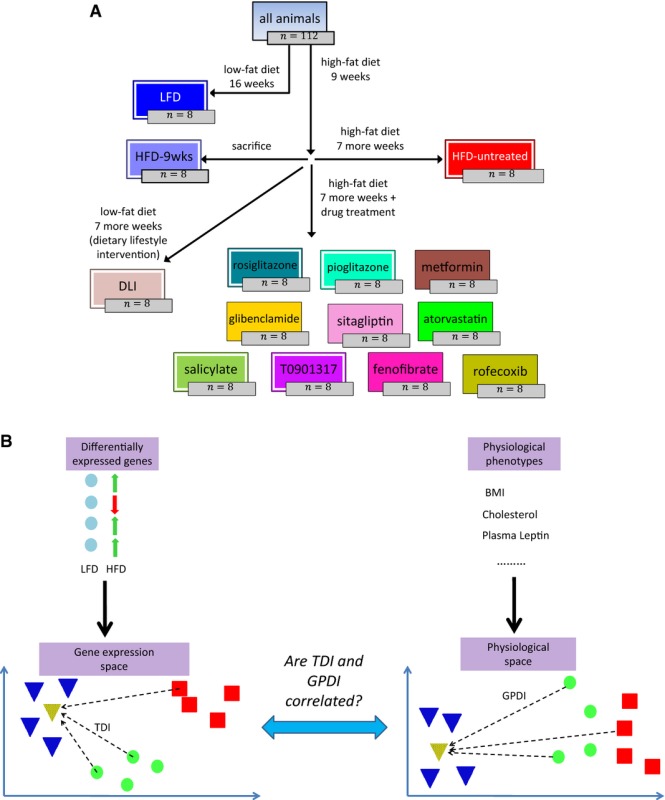

A control group of mice was kept on low-fat maintenance chow diet (LFD) throughout the experiment (16 weeks in total). The other mice were fed high-fat diet (HFD) for 9 weeks to establish dyslipidemia-associated disease markers and were then divided between the following intervention regimens: One group was sacrificed immediately (9-week HFD group) and represents the time-point at which interventions were commenced. The other mice were continued on HFD for 7 more weeks during which they were either left untreated (HFD-untreated) or treated with one of 10 relevant drugs (see below). One group of mice was switched after 9 weeks from HFD to LFD (again, for 7 weeks) and represents a dietary lifestyle intervention (DLI). Hepatic gene expression was measured in all animals. In addition, white adipose gene expression was measured in 4 of the pharmacological intervention groups (rosiglitazone, pioglitazone, T0901317, and salicylate), as well as in the LFD, 9-week HFD, HFD-untreated, and DLI groups.

The ten drugs studied here are all highly relevant to the disease in question (Table1, Supplementary Table S2). Eight of the drugs are FDA-approved and commonly prescribed to treat the metabolic syndrome in human patients. These include oral anti-diabetic compounds that work through diverse mechanisms (metformin, glibenclamide, sitagliptin, rosiglitazone, pioglitazone), lipid-modulating compounds (fenofibrate, atorvastatin), and the anti-inflammatory salicylate; salicylic acid is the main active compound in aspirin, which is prescribed to prevent atherosclerotic complications (Awtry & Loscalzo, 2000; Campbell *et al*, 2007; American Diabetes Association, 2013). One drug (T0901317) is an experimental compound that has been shown to exert anti-atherogenic effects in the mouse model studied here (Calkin & Tontonoz, 2010), and the last one (rofecoxib) is an anti-inflammatory compound, included because of the critical role of inflammation in atherosclerotic disease (Ross, 1999; Libby *et al*, 2002; Hansson & Hermansson, 2011; Weber & Noels, 2011; van Diepen *et al*, 2013).

**Table 1:** Drug compounds studied in this work.

Category	Drug	Status	Notes
Anti-diabetic	Metformin	Approved	Improve glycemic control
	Glibenclamide	Approved	
	Sitagliptin	Approved	
	Rosiglitazone	Approved	
	Pioglitazone	Approved	
Lipid-modulating	Fenofibrate	Approved	Reduces plasma triglycerides as well as low-density lipoprotein (LDL) cholesterol levels and increases plasma high-density lipoprotein (HDL) cholesterol levels
	T0901317	Experimental	A synthetic liver X receptor (LXR) agonist which is known to exert an anti-atherogenic effect in LDLR^−/−^ mice (Calkin & Tontonoz, 2010). LXR modifiers are actively studied as pharmacological targets for treating atherosclerosis (Hong & Tontonoz, 2014)
	Atorvastatin	Approved	Lowers plasma LDL cholesterol levels
Anti-inflammatory	Salicylate	Approved	A chronic inflammatory response underlies the metabolic syndrome and the progression of atherosclerotic disease (Libby *et al*, 2002; Hansson & Hermansson, 2011; Lumeng & Saltiel, 2011; Harper *et al*, 2013; Van Diepen *et al*, 2013; McNelis & Olefsky, 2014). Hence, anti-inflammatory drugs were expected to alleviate cardiovascular complications.
	Rofecoxib	Withdrawn several years after FDA approval due to an increased risk of cardiovascular events (Praticò & Dogné, 2005)	Note that salicylic acid is the main active compound in aspirin, which is commonly prescribed to prevent atherosclerotic complications (Awtry & Loscalzo, 2000; Campbell *et al*, 2007; American Diabetes Association, 2013)

Data are taken from Drugbank (Law *et al*, 2014) (accessed July 2014) except where noted otherwise. See Supplementary Table S2 for more detailed information.

Radonjic *et al* (2013) have recently analyzed a subset of these data and compared the effects of the pharmacological interventions with those of the dietary intervention. They concluded that the pharmacological interventions generally reduced the HFD-induced hyperglycemia, and yet had only a limited effect on other cardiovascular risk markers, whereas the dietary intervention attenuated all risk markers. Transcriptomic and metabolomic analyses of disease pathways confirmed a reversal of HFD-induced disturbances to the liver under DLI. Here, we utilize an extended collection of the data reported by Radonjic *et al* to rigorously investigate the correlation between the reversal of molecular disease phenotypes and the reversal of physiological disease phenotypes across multiple treatment groups.

We considered the LFD group to be representative of a low-risk baseline (for simplicity of explanation, we refer to these mice as ‘healthy’ despite their genetic deficiency). The untreated 16-week HFD group (henceforth designated ‘untreated HFD’ for brevity) was considered representative of an elevated risk to develop cardiovascular disease. To assess the success of each drug in restoring the normal (LFD) transcriptome, we first identified the genes that were most differentially expressed between the HFD and LFD groups in each of the two tissues, and are thus implicated in diet-induced pathologies and higher cardiovascular risk. These genes were used to define a Euclidean *gene expression space* for each of the two tissues, onto which we projected the gene expression vector of each animal (Materials and Methods, Discussion). We then computed the (tissue-specific) *transcriptome deviation index* (TDI) of each animal—the distance in the gene expression space between the animal and the centroid of the healthy (LFD) animals (Box 1B, Materials and Methods). The smaller the TDI, the closer is the animal's transcriptome to the normal, low-risk state. We defined a physiological analog to the TDI based on the aggregate data of the 26 disease-relevant physiological biomarkers measured in the study animals (Supplementary Table S1). These markers represent metabolic abnormalities shown to predispose patients to the development of type 2 diabetes and cardiovascular disease and are instrumental in the clinical management of human patients. We used the aggregated physiological data to compute a global physiological deviation index (GPDI) (Box 1B, Materials and Methods). The smaller is an animal's GPDI, the closer is its physiology to the normal baseline. Our key hypothesis, namely that a restoration of the normal transcriptome following a clinical intervention is correlated with a restoration of the normal physiology, translates into the hypothesis that a direct correlation exists between the TDIs and GPDIs across the studied animals.

### The restoration of gene expression correlates with a restoration of physiological markers to their baseline levels

**Figure 1:**
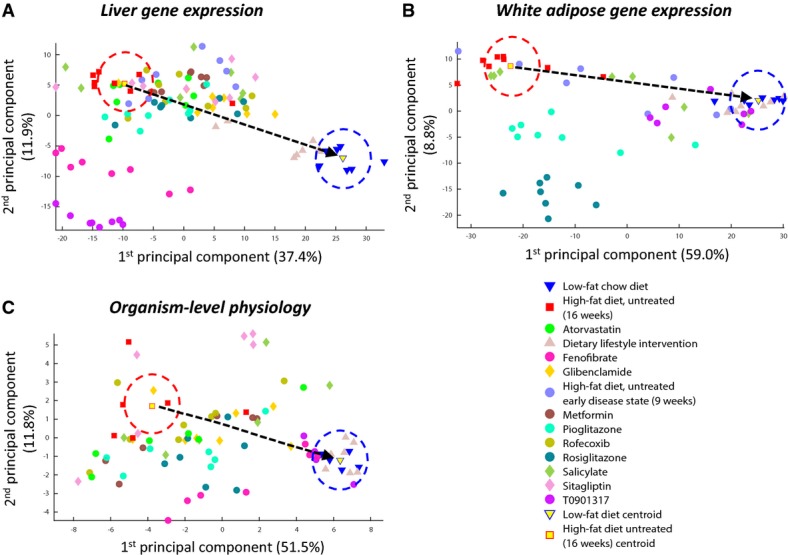
The gene expression and physiological spaces

The projection of the animal data into the transcriptomic and physiological spaces allowed us to study their relationship (Fig1, Supplementary Figs S1 and S2). We first examined the post-treatment transcriptomic changes in the hepatic and adipose gene expression spaces separately to obtain a tissue-specific view (Fig1A and B). Many of the treatments altered the gene expression of the treated HFD groups in a way that brought it closer to the gene expression of the LFD group (Supplementary Results S1, Supplementary Fig S1). This observation, which accords with the conceptual image depicted in Box 1B, can also be seen in Fig1A and B: Most dots are closer to the LFD mean (circled in blue) than are the untreated HFD animals. Nonetheless, there exists considerable variability between transcriptomic effects on the animals in each treatment group (Supplementary Results S2, Supplementary Figs S3 and S4). Quite remarkably, the dietary lifestyle intervention was the most effective in reversing the transcriptome back to its healthy state in both tissues, as well as in reinstating the healthy physiology (Fig1, Supplementary Figs S1 and S2), in line with the results of Radonjic *et al* (2013).

A-C The figure shows the first two principal components of (A) the gene expression space of the liver, (B) the gene expression space of white adipose tissue, and (C) the physiological space. In all panels, each dot represents one animal; color codes denote the different experimental groups. Red squares represent the HFD animals, and blue triangles represent the LFD animals. The dashed arrow connects the HFD centroid (yellow square, circled in red) to the LFD centroid (yellow triangle, circled in blue) and denotes the direction of a reversal of the gene expression or physiological state back to the norm.

Source data are available online for this figure.

Gene set enrichment analysis (GSEA; Subramanian *et al*, 2005) corroborated that pathways that were expected to be modulated by some of the drugs were indeed differentially expressed (except in the case of metformin, which may be due to the lack of statistical power). Interestingly, two particular observations that have been recently reported in other animal models were also detected by this analysis: dysregulation of hepatic ribosomal activity in HFD-fed mice, as in Oie *et al* (2014), and suppression of the complement and coagulation systems by T0901317, as in Sukardi *et al* (2012). See Supplementary Results S3 and Supplementary Tables S3 and S4 for details (see also Supplementary Results S4.1 for an alternative definition of TDI via GSEA).

**Figure 2:**
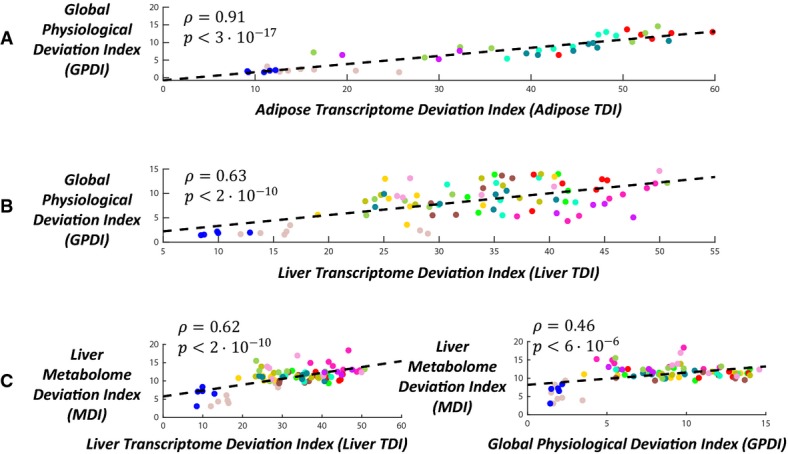
Post-treatment deviation from the baseline omic state (e.g. in gene expression) is correlated with a deviation from the baseline physiology

Examining the relationship between the transcriptomic and physiological changes, we find that both the adipose TDI and the hepatic TDI are correlated with the GPDI, as hypothesized. The correlation is particularly strong in the adipose case (Fig2A and B; Pearson rho = 0.91, 0.63 for adipose and liver, respectively; *P*-values <3e-17, 2e-10; all the results hold when Spearman correlations are used instead of Pearson correlations, see Supplementary Fig S6A and B). This indicates that HFD-induced cardiovascular risk is associated with both transcriptomic and physiological deviations from the norm and that its alleviation by extant treatments is correlated with a restoration of the norm at both levels. The hepatic metabolome was also measured in the animals (Materials and Methods) and represents another dimension of the molecular-level disease state. We projected these data into a *metabolome space*, and computed *metabolome deviation indices* (MDI) in a manner analogous to that of the transcriptomic and physiological data (Materials and Methods, Supplementary Fig S7). The hepatic MDI is significantly correlated both with the hepatic TDI (Fig2C, Supplementary Fig S6C; Pearson rho = 0.62, *P* < 2e-10) and with the GPDI (Pearson rho = 0.46, *P* < 6e-6). However, it is not correlated with the adipose TDI (Pearson rho = 0.15, *P* = 0.17).

A, B The correlation between the global physiological deviation index (GPDI) and the transcriptomic deviation index (TDI) in (A) white adipose tissue and (B) liver tissue.

C Left panel: the correlation between the liver metabolome deviation index (MDI) and the liver TDI. Right panel: the correlation between the liver MDI and the GPDI.

Data information: Each dot represents one animal; color codes denote the different experimental groups as in Fig1. Pearson correlations and their respective *P*-values are noted in each panel, as well as a linear regression line. All the results hold when Spearman correlations are used instead (see Supplementary Fig S6). Source data are available online for this figure.

**Figure 3:**
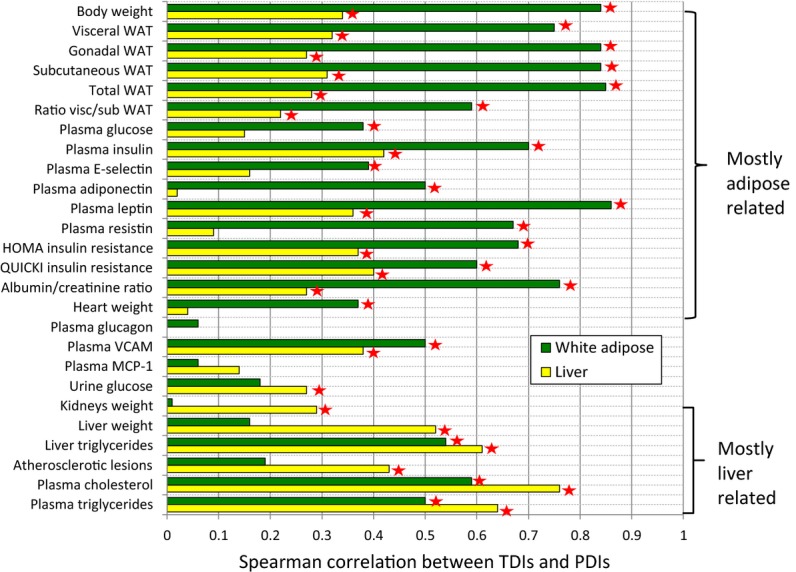
Deviations from the baseline gene expression and deviations from the baseline physiology are correlated in a tissue-specific manner Deviations from the baseline gene expression (TDIs) in liver and white adipose tissues are correlated with deviations from the normal physiology (PDIs) for markers that are known to be associated with those tissues. Bar lengths represent the Spearman correlations between the TDIs of the two tissues and PDIs of 26 different physiological markers. WAT stands for white adipose tissue, ratio visc/sub WAT for ratio of visceral to subcutaneous WAT. Asterisks mark statistically significant correlations (using the Benjamini–Hochberg correction for multiple hypotheses testing with a 5% FDR level). The top part of the figure displays markers whose PDI is more correlated with white adipose TDI than with hepatic TDI. The bottom part shows markers with a reverse tissue-specific pattern. Source data are available online for this figure.

We next turned to examine the correlation between the TDIs of the animals and individual physiological markers; for this purpose, we computed an individual *physiological deviation index* (PDI) for each of the markers (Materials and Methods). Overall, high correlations were observed and had noticeable tissue-specific trends (Fig3, Supplementary Table S7; Supplementary Figs S8 and S9; *P*-values were adjusted to multiple hypotheses testing according to the Benjamini–Hochberg FDR method, and false discovery rate was set at α = 5%; Spearman correlations, which are more robust than Pearson correlations in the presence of outliers, were used here because several animals had outlier physiological measurements in particular markers). The white adipose and hepatic TDIs were significantly correlated with 20 and 19 PDIs out of the 26 measured, respectively. The adipose transcriptome was most highly correlated with the animals’ total body weight, total white adipose weight, and plasma leptin levels (Spearman rho = 0.84, 0.85, 0.86, adjusted *P* < 2.6e-17, 1e-17, 2.6e-17). The latter is a hormone that is produced predominantly by white adipose tissue, and whose blood levels correlate well with total body fat (Considine *et al*, 1996; Klok *et al*, 2007; Myers *et al*, 2008). The hepatic transcriptome was most highly correlated with plasma cholesterol, plasma triglycerides, and liver triglycerides levels (Spearman rho = 0.76, 0.64, 0.61, adjusted *P* < 2.5e-21, 1.8e-13, 5.5e-12, respectively), reflecting the liver's central role in lipid metabolism (liver MDI was correlated with these markers’ PDIs as well, see Supplementary Table S8 and Supplementary Figs S10 and S11).

Significant correlations were also identified between TDIs and PDIs of known hallmark markers of cardiovascular risk: Albuminuria is correlated with adipose TDI, and atherosclerotic lesion size is correlated with hepatic TDI. Albuminuria is an established predictor for cardiovascular morbidity and mortality, as well as for poor renal outcome, which is often diagnosed via the albumin/creatinine ratio in the urine (Gerstein *et al*, 2001; Isomaa *et al*, 2001; Mattix *et al*, 2002; Bahrami *et al*, 2008; Basi *et al*, 2008). Interestingly, there exists in our data a strong correlation between the PDI of the albumin/creatinine ratio and the adipose TDI (Spearman rho = 0.76, adjusted *P* < 2.4e-10; only a weaker correlation was obtained with the hepatic TDI). This result is in accordance with recent findings concerning the role of adipose-derived hormones, and particularly adiponectin, in the development of albuminuria and renal dysfunction (Tsioufis *et al*, 2005; Sharma *et al*, 2008; Sharma, 2009; Ix & Sharma, 2010; Meyvis *et al*, 2013; Christou & Kiortsis, 2014). Liver TDI was significantly correlated with the PDI of atherosclerosis (Spearman rho = 0.43, adjusted *P* < 1.2e-5), which in turn triggers myocardial infarction and stroke (Weber & Noels, 2011). This reflects the causative role of dyslipidemia and liver inflammation in the formation of atherosclerotic lesions (Tannock *et al*, 2005; Kleemann *et al*, 2007; Harper *et al*, 2013; Van Diepen *et al*, 2013).

**Figure 4:**
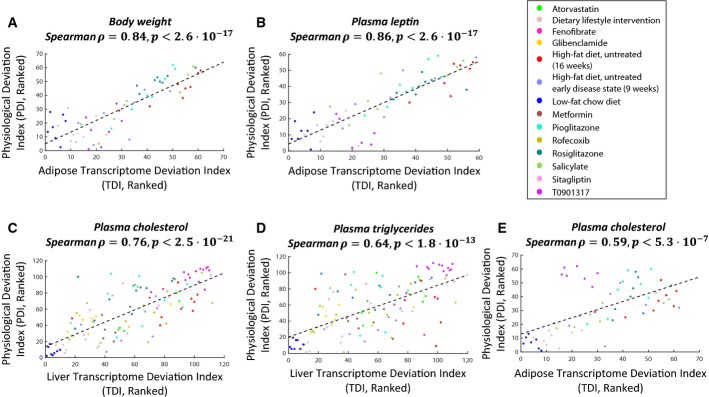
Deviations from the baseline gene expression and deviations from the baseline physiology are correlated across different experimental groups and treatment regimens

Notably, the associations between TDIs and PDIs hold across all the experimental groups: animals in different phases of disease progression, treated and untreated animals, and treatments that work through different mechanisms (Fig4A–D; four of the physiological markers for which particularly high correlations with TDIs were obtained are presented in this figure; the complete data are provided in Supplementary Table S7 and Supplementary Figs S8 and S9). This indicates that effective pharmacological interventions, which work through diverse mechanisms, ultimately serve to reverse the transcriptomic deviations from the healthy baseline, and that these effects are associated with a restoration of the normal physiology. The same holds also for the non-pharmacological, dietary intervention. Exceptions do occur, however. For example, the drug T090137 does not adhere to the general correlation trend between the adipose TDI and the plasma cholesterol and triglyceride PDIs (Fig4E). This probably stems from this drug's particular mechanism of action—it is an agonist of a transcription factor that has a pivotal role in lipid metabolism (Schultz *et al*, 2000; Steffensen & Gustafsson, 2004; Ulven *et al*, 2005; Zhao & Dahlman-Wright, 2010). This drug also has particularly high hepatic TDIs compared with other treatment groups, which again point to an interference with lipid metabolism (Fig1A, Supplementary Figs S1A and S12A, and see below).

A-D White adipose (A, B) and liver (C, D) TDIs (deviations from the baseline gene expression) are correlated with PDIs (deviations from the baseline physiology) across different experimental groups and treatment regimens. PDIs of four physiological markers are shown, two that are highly correlated with adipose transcriptome (A, B) and two that are highly correlated with hepatic transcriptome (C, D).

E Adipose TDIs and plasma cholesterol PDIs are generally correlated, but this does not hold for animals treated with T090137 (purple dots at the top left corner of the figure), probably due to this drug's particular mechanism of action (see main text). The correlation displayed in (E) increases from 0.59 to 0.8 (*P* < 1.3e-13) when excluding the T090137 animals.

Data information: Each dot represents one animal; color codes denote the different experimental groups. The dashed lines are linear regression lines. The complete data are available in Supplementary Table S7 and in Supplementary Figs S8 and S9. *P*-values are adjusted to multiple hypotheses testing as described in the main text. Source data are available online for this figure.

### Non-restorative alterations to the gene expression are associated with unfavorable outcomes

Four drugs in our data introduced large transcriptomic alterations that are incongruent with the direction of the disease reversal axis that goes from the HFD centroid to the LFD centroid (such alterations will be henceforth referred to in short as “non-restorative”): pioglitazone and rosiglitazone in white adipose, and fenofibrate and T090137 in the liver (Fig1A and B; Supplementary Figs S12 and S13A–C). This seems to stem from their respective mechanisms of action—each of these drugs modulates a master transcription regulator, which is highly expressed in the tissue in which it causes the major non-restorative transcriptomic shifts (Supplementary Table S2). In contrast, the dietary intervention, which was the most successful one in reversing disease gene expression patterns, was also characterized by small non-restorative transcriptomic effects in both tissues when compared with the other intervention groups. We hypothesized that non-restorative shifts in gene expression are associated with unfavorable physiological outcomes.

A comprehensive test of this hypothesis is impossible in the current dataset: The physiological indices collected in the study animals were selected in the first place because of their relevance to the disease model, and this precludes us from studying unfavorable effects that arise solely as a side effect of the drugs rather than outcomes of the HFD feeding (Supplementary Table S9). Therefore, we resorted to considering only a limited scope of unfavorable drug effects that are detectable in the set of 26 physiological markers measured in the study animals (the same ones that had been analyzed throughout this paper, Supplementary Table S1). We considered a marker to indicate the presence of an unfavorable treatment outcome if it fulfilled two conditions: (i) Its levels in the HFD group were higher than in the LFD group, and (ii) its levels in any of the treatment groups were even higher than in the HFD group in a statistically significant manner (one-sided *t*-test; a parallel criterion was used for markers in which the HFD levels were lower than the LFD levels; *P*-values were adjusted to multiple hypotheses testing according to the Benjamini–Hochberg method, and false discovery rate was set at α = 5%). In other words, a marker was considered as manifesting an unfavorable outcome of a certain treatment if its values in the treated animals were even farther from the baseline than its values in the untreated HFD animals (Supplementary Fig S13D). We found that according to this definition, four drugs were associated with statistically significant unfavorable physiological outcomes: pioglitazone, rosiglitazone, fenofibrate, and T090137 (Supplementary Fig S14). Notably, these are the same drugs that were previously shown to introduce the highest non-restorative transcriptomic alterations.

The unfavorable outcomes ascribed to these drugs by our analysis were all previously described in the medical literature. Moreover, the unfavorable outcomes ascribed to each drug concern physiological markers that are known to be associated with the tissue in which it causes major non-restorative transcriptional shifts. T0901317 and fenofibrate introduced large non-restorative alterations to hepatic gene expression. Indeed, our analysis implicated T0901317 in hepatomegaly (i.e., liver enlargement), atherogenesis, and abnormally high plasma cholesterol and plasma triglyceride levels (Chisholm *et al*, 2003; Jung *et al*, 2011), all indicative of an underlying liver pathology. The presence of drug-induced hepatotoxicity is further corroborated by the observation that T0901317 treatment also led to significant up-regulation, compared with the untreated HFD animals, of several pro-inflammatory genes (Supplementary Results S5). Fenofibrate was implicated in hepatomegaly and elevated plasma cholesterol (but not triglyceride) levels (Toda *et al*, 2003; Backes *et al*, 2007). Treatment with the two TZDs, pioglitazone and rosiglitazone, led to large non-restorative alterations in adipose gene expression. These treatments also led to abnormally high levels of the hormone adiponectin, which is secreted exclusively by adipocytes (Chandran *et al*, 2003; Lihn *et al*, 2005; Ye & Scherer, 2013). While increased adiponectin levels are associated with reversal of insulin resistance (Yamauchi *et al*, 2001; Gao *et al*, 2013) and are thus considered a beneficial effect of TZD administration (Maeda *et al*, 2001; Hirose *et al*, 2002; Yang *et al*, 2002), the data studied here suggest that TZDs increase them far beyond (3–6 fold) normal levels. Notably, irregularly high adiponectin levels have been reported to correlate with increased cardiovascular disease and all-cause mortality (Laughlin *et al*, 2007; Jorsal *et al*, 2008; Ohashi *et al*, 2008). We conclude that in the animal model studied here, treatments that induce non-restorative gene expression alterations tend to be associated with unfavorable physiological outcomes.

## Discussion

In this paper, we examined whether effective drug treatments, which restore various organism-level physiological indices to their norm, also serve to reverse molecular-level, disease-induced gene expression deviations from the norm. This assumption has profound implications to clinical practice. First, because restoration of disease-relevant physiological markers to baseline levels is construed as a sign of reduced risk, and hence as a sign of the treatment's efficacy. Second, because this assumption has already been made implicitly by successful studies that sought to identify novel drug therapies by searching for drug candidates whose molecular effects anti-correlate with disease molecular signatures (Hu & Agarwal, 2009; Boyle *et al*, 2010; Chang *et al*, 2010; Josset *et al*, 2010; Dudley *et al*, 2011; Kunkel *et al*, 2011; Sirota *et al*, 2011; Qu & Rajpal, 2012; Jahchan *et al*, 2013; Pacini *et al*, 2013; Yizhak *et al*, 2013). While this assumption seems reasonable enough, it is important to test it directly in a quantitative, systematic manner.

To this end, we studied data from a mouse model of diet-induced dyslipidemia and atherosclerosis that was subject to various drug treatments; both gene expression and physiological data were analyzed and compared. Overall, the data provide strong support to this hypothesis: Treatments that restore gene expression patterns to their norm are also more successful in restoring the physiological markers to their baselines. This observation holds in a tissue-specific manner, at least with respect to the two tissues studied here: Treatments that reverse the transcriptomic signatures of the disease in a particular tissue were associated with positive physiological effects in that tissue. For example, the LXR ligand T0901317 induced major transcriptional shifts in both liver and adipose. The shifts were mostly disease-reversing in adipose tissue, but not in the liver. Accordingly, mice treated with T0901317 were characterized by favorable adipose-related effects, including reduced body weight and normal levels of the adipose-derived hormones leptin, resistin, and adiponectin. However, they also exhibited adverse phenotypes consistent with drug-related impaired liver functions, such as elevated liver weight and blood cholesterol levels, which were both higher than those of untreated high-fat diet animals. A related and particularly troubling observation was that the T0901317 group had significantly larger atherosclerotic plaques than the untreated high-fat diet group.

One should note, however, that our findings are based only on the animal model studied here. While we hypothesize that they are more generally applicable, it remains for future work: (i) to extend them to other diseases and other organs than those studied here, (ii) to test them in human subjects, and (iii) to extend them to other omic data types that capture other facets of the molecular-level drug effects, as well as to other clinically relevant physiological indices. Using more sophisticated dimensionality reduction methods than the one employed here may prove illuminating. In addition, one notes that this study established only an association between treatment effects at the molecular and physiological levels; any causative relationship between the two levels is probably complex and multifaceted.

A major challenge left for future work is the incorporation of adverse drug side effects into the methodological framework employed in this study. We have shown that drugs that induce large non-restorative shifts in gene expression are associated, to some extent, with unfavorable physiological outcomes. We hypothesize that, more generally, the non-restorative gene expression alterations brought about by the drug may be correlated with the severity of the drug's side effects. The limited scope of the present data does not allow testing this hypothesis directly: Adverse drug side effects may manifest in biomarkers that are unaffected by the disease itself, whereas in the experiment analyzed here, only disease-relevant biomarkers were measured. Similarly, a study of side effects should consider genes that are unaffected by the disease itself but may still be affected by the drug treatment, while here we reduced the data's dimensionality by focusing only on disease-associated genes that were differentially expressed between the LFD and untreated HFD groups. Dimensionality reduction was necessary because distance metrics become unstable in high dimension (Beyer *et al*, 1999; Hinneburg *et al*, 2000; Aggarwal *et al*, 2001). Future studies that wish to consider adverse drug side effects may overcome the dimensionality challenge by augmenting the data-driven approach presented here with knowledge-based elements; that is, incorporating into the gene expression spaces known pathways and gene modules that are not disrupted by the disease, but might be related to adverse drug side effects.

In conclusion, the contribution of this study is twofold: First, it provides a sound basis to *in silico* methods that rely on omic metrics for drug repurposing and drug discovery; such methods are expected to develop into a cornerstone of future pharmacological research (Hurle *et al*, 2013; Iorio *et al*, 2013). Second, our results imply that drugs that are most successful in reversing the transcriptomic signatures of the disease across multiple tissues should also exert the most favorable outcomes. This highlights the need to develop drugs that restore the global cellular state of multiple tissues back to their healthy norms, rather than rectify particular disease phenotypes.

## Materials and Methods

### Data collection and processing

A detailed description of the experimental design, the pharmacological intervention regimens, and methods of data collection was given by Radonjic *et al* (2013). In summary, the experiment consisted of 132 animals (15 animals in the HFD-untreated group and 9 in all the other groups), but hepatic mRNA was collected only from 112 animals (8 per group). The other 20 animals were excluded from the current analysis. Out of these 112 animals, white adipose mRNA was collected only from 62 animals from 7 experimental groups as detailed in the main text (and see also Box 1A). White adipose was taken from the gonadal depot. In addition, 168 liver metabolites were measured for each animal, refer to Radonjic *et al* (2013) for details.

Microarray data were background-corrected, log2-transformed, and quantile-normalized by the lumi package of R/Bioconductor (Du *et al*, 2008). Statistical tests for differentially expressed genes were run through the limma package (Smyth, 2005). Liver and adipose data are publicly available at the ArrayExpress (Accession Number E-MTAB-1063) and GEO (Accession Number GSE57659) repositories, respectively.

### The gene expression space and the transcriptome deviation index

The top *N *=* *200 differentially expressed probes between the untreated 16-week HFD group and the LFD group were selected (Supplementary Tables S5 and S6; we verified that our results were not sensitive to the choice of *N* within an order of magnitude). *Z*-scores of their respective normalized expression (across all the study animals) were transformed by principal components analysis (PCA), with feature weights as described below, to form the gene expression space. All distance computations in this study used all the dimensions of the PCA space; that is, we did not use only the top principal components. We verified that using only the top principal components did not significantly alter the results.

In order to account for the different power that the selected *N* = 200 probes might have to separate the LFD and the untreated HFD groups, we weighted the features when computing the PCA by their Fisher scores, which are defined as follows:

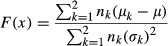
with *k* = 1,2 being indices for the two groups (LFD and untreated HFD). *F*(*x*) is the Fisher score of a particular feature *x* whose mean across both groups is *μ*. *μ*_*k*_ and *σ*_*k*_ denote the mean and standard deviation, respectively, of the feature in group *k*. *n*_*k*_ denotes the number of observations in group *k*. We note that the conclusions presented in this paper are robust to changes in the method by which features are weighted, and uphold even when assigning equal weights to all features.

Once the gene expression space has been defined, we project the gene expression data of all animals onto it. We then compute the centroid of all the LFD animals in this space, which represents the healthy baseline. The transcriptome deviation index (TDI) associated with a particular animal is the Euclidean distance between its representation in the gene expression space and the LFD centroid. We emphasize that the computation of the gene expression space and TDIs was done separately for each of the studied tissues.

### Physiological and metabolome deviation indices

The physiological space was defined in an analogous manner to the gene expression space. *Z*-scores were computed for each of the 26 physiological markers collected from the animals (Supplementary Table S1), and PCA-transformed with features weighting as described above. Again, all principal components, rather than only the top ones, were used to define distances in the PCA space. The global physiological deviation index (GPDI) of a particular animal is the Euclidean distance between its representation in the physiological space and the LFD centroid. Animals for which not all of the 26 markers were available (due to failing the quality control of at least one assay) were excluded from GPDI analyses (29 out of 112). PDI is a special case of the GPDI that pertains to only one physiological marker. It is defined as the absolute value of the difference between the value of this marker in a particular animal and the mean of the marker's values in the LFD animals. The metabolome space and metabolome deviation indices were computed by the same procedure as the gene expression and physiological ones. Metabolites whose values were not available for all animals (31 out of 168) were excluded from MDI analyses.

### Non-restorative gene expression alterations

Let *v*_LFD_, *v*_HFD_ be the centroids of the LFD and untreated HFD groups, respectively, in the gene expression space. The direction *v*_LFD_ − *v*_HFD_ can be considered the desired effect of the treatment, which corresponds to a reversal of the disease gene expression phenotypes; effects that occur in orthogonal directions are termed non-restorative. The gene expression vector *v* of a treated animal can be thus decomposed into two orthogonal components: *v*'s projection onto *v*_LFD_ − *v*_HFD_, which we denote *v*_1_, and *v*_2_ = *v *− *v*_1_. ||*v*_1_||_2_ and ||*v*_2_||_2_ are the magnitudes of the disease-reversal (i.e., restorative) and non-restorative alterations, respectively, brought about by the treatment (see also Supplementary Fig S13A).

### Implementation

The analysis was implemented in Matlab version 8.1.0.604 (R2013a). The source code used to compute the gene expression spaces, TDIs, and their correlations with PDIs can be downloaded from http://www.cs.tau.ac.il/~allonwag/LDLR_paper/LDLR_paper_code.zip. The source code is also available, along with the gene expression, physiological, and metabolome data, in Supplementary Dataset S1.

## Supporting Information

Supplementary Dataset S1

Review Process File
