# Supplementary material for: Drugs that reverse disease transcriptomic signatures are more effective in a mouse model of dyslipidemia
Source: Mol Syst Biol. 2015 Mar 3;11(3):0791. doi: 10.15252/msb.20145486 (PMC4380926; doi:10.15252/msb.20145486)
Supplement: Supplementary file 1 [file msb0011-0791-sd1.pdf]

Supplementary Information

Drugs that reverse disease transcriptomic signatures are more effective in a mouse model of dyslipidemia

Allon Wagner[1,2,*], Noa Cohen[1], Thomas Kelder[3,#], Uri Amit[4,5], Elad Liebman[6], David M. Steinberg[7], Marijana Radonjic[3,#] & Eytan Ruppin[1,8,9,*]

[1] The Blavatnik School of Computer Science, Tel Aviv University, Tel Aviv 69978, Israel;
[2] Department of Electrical Engineering and Computer Science, University of California, Berkeley, Berkeley, CA 94720-1770, USA;
[3] Microbiology and Systems Biology, TNO, 3700 AJ Zeist, the Netherlands;
[4] Neufeld Cardiac Research Institute, Tel Aviv University, Tel Aviv 69978, Israel;
[5] Regenerative Medicine Stem Cells and Tissue Engineering Center, Sheba Medical Center, Tel Hashomer 52621, Israel;
[6] Department of Computer Science, University of Texas at Austin, Austin, TX 78712, USA;
[7] Department of Statistics and Operations Research, Tel Aviv University, Tel Aviv 69978, Israel;
[8] The Sackler School of Medicine, Tel Aviv University, Tel Aviv 69978, Israel;
[9] Department of Computer Science, Institute of Advanced Computer Sciences (UMIACS) & the Center for Bioinformatics and Computational Biology, University of Maryland, College Park, MD 20742, USA;
[#] Current affiliation: EdgeLeap B.V., Hooghiemstraplein 15, 3514 AX Utrecht, the Netherlands;
[*] **To whom correspondence should be addressed**.
E-mail: (AW) allonwag@post.tau.ac.il; (ER) ruppin@post.tau.ac.il

Table of Contents

TABLE OF CONTENTS 2

SUPPLEMENTARY RESULTS 4

1. REVERSAL OF DISEASE PHENOTYPES IN THE GENE EXPRESSION SPACES 4
2. INTRA-GROUP VARIABILITY 4
3. GSEA ANALYSIS 5
4. ALTERNATIVE DEFINITIONS OF TDI 7
4.1. GSEA-BASED METHODS 7
4.2. GENES MAPPED TO MULTIPLE PROBES 8
5. UP-REGULATION OF PRO-INFLAMMATORY GENES IN THE T0901317 TREATMENT GROUP 8

SUPPLEMENTARY FIGURES 10

1. SUPPLEMENTARY FIGURE 1: TRANSCRIPTOME DEVIATIONS INDICES 10
2. SUPPLEMENTARY FIGURE 2: GLOBAL PHYSIOLOGICAL DEVIATION INDICES 11
3. SUPPLEMENTARY FIGURE 3: HIERARCHICAL CLUSTERING IN THE LIVER GENE EXPRESSION SPACE 12
4. SUPPLEMENTARY FIGURE 4: HIERARCHICAL CLUSTERING IN THE ADIPOSE GENE EXPRESSION SPACE 14
5. SUPPLEMENTARY FIGURE 5: CORRELATION BETWEEN GSEA-BASED SCORES AND TDIS 15
6. SUPPLEMENTARY FIGURE 6: SPEARMAN CORRELATIONS OF TDI, GPDI, AND MDI 16
7. SUPPLEMENTARY FIGURE 7: LIVER METABOLOME SPACE 17
8. SUPPLEMENTARY FIGURE 8: WHITE ADIPOSE TDI CORRELATION WITH INDIVIDUAL PDIS 18
9. SUPPLEMENTARY FIGURE 9: LIVER TDI CORRELATION WITH INDIVIDUAL PDIS 19
10. SUPPLEMENTARY FIGURE 10: LIVER MDI CORRELATION WITH INDIVIDUAL PDIS 20
11. SUPPLEMENTARY FIGURE 11: LIVER MDI CORRELATION WITH INDIVIDUAL PDIS (SCATTER PLOTS) 21
12. SUPPLEMENTARY FIGURE 12: DRUGS THAT INDUCE MAJOR NON-RESTORATIVE GENE EXPRESSION ALTERATIONS 22
13. SUPPLEMENTARY FIGURE 13: NON-RESTORATIVE GENE EXPRESSION ALTERATIONS ARE ASSOCIATED WITH UNFAVORABLE PHYSIOLOGICAL OUTCOMES 23
14. SUPPLEMENTARY FIGURE 14: UNFAVORABLE DRUG OUTCOMES IN PHYSIOLOGICAL MARKER DATA 25

SUPPLEMENTARY TABLES 26

1. SUPPLEMENTARY TABLE 1: LIST OF PHYSIOLOGICAL MARKERS MEASURED IN THE STUDY ANIMALS 26
2. SUPPLEMENTARY TABLE 2: DRUG MECHANISM OF ACTION 28
3. SUPPLEMENTARY TABLE 3: GSEA ANALYSIS OF THE LIVER TRANSCRIPTOME 30
4. SUPPLEMENTARY TABLE 4: GSEA ANALYSIS OF THE ADIPOSE TRANSCRIPTOME 34

5. SUPPLEMENTARY TABLE 5: PROBES USED TO CONSTRUCT THE LIVER GENE EXPRESSION SPACE 37
6. SUPPLEMENTARY TABLE 6: PROBES USED TO CONSTRUCT THE ADIPOSE GENE EXPRESSION SPACE 43
7. SUPPLEMENTARY TABLE 7: CORRELATIONS OF LIVER AND ADIPOSE TDIS WITH INDIVIDUAL PDIS 49
8. SUPPLEMENTARY TABLE 8: CORRELATIONS OF LIVER MDI WITH INDIVIDUAL PDIS 50
9. SUPPLEMENTARY TABLE 9: ADVERSE SIDE-EFFECTS OF DRUGS IN THE CURRENT STUDY 51

SUPPLEMENTARY REFERENCES 59

Supplementary Results

1. Reversal of disease phenotypes in the gene expression spaces

It is reasonable to assume that effective clinical interventions should reverse disease-induced pattern to the gene expression of affected organs. We therefore tested whether the treated animals tended to lie closer in the gene expression space to the healthy (LFD) group than did the untreated HFD group (see also Supplementary Figure 1). Formally, for each treatment group we computed the TDIs of animals in that group, and ran a two-sample t-test between them and the TDIs of the untreated HFD (16 weeks) group. The following table presents the one-sided p-values with which the null hypothesis of equal means for the TDIs of both groups can be rejected in favor of the alternative that the treatment led to a smaller mean TDI. P-values were adjusted to multiple hypotheses testing by the Benjamini-Hochberg (BH) method.

Experimental group	Liver BH-adjusted p-value	Adipose BH-adjusted p-value
Dietary intervention (DLI)	0.0001	4e-7
Rosiglitazone	0.0287	0.1560
Pioglitazone	0.0918	0.0516
Metformin	0.0189	N/A
Glibenclamide	0.0094	N/A
Sitagliptin	0.1081	N/A
Atorvastatin	0.2065	N/A
Salicylate	0.1081	0.0378
T0901317	0.9338	2e-5
Fenofibrate	0.9338	N/A
Rofecoxib	0.0469	N/A

2. Intra-group variability

Figure 2a-b suggests that there exists considerable variability in the transcriptomic effects of some of the treatments. For example, animals treated with salicylate do not tend to cluster together in both adipose and the liver, whereas animals treated with rosiglitazone tend to cluster together in the gene expression space. In order to better study intra-group variability, hierarchical clustering of the animals in the gene expression space was conducted. Results are shown in Supplementary Figures 3-4. Dendrograms were created with the Euclidean metric, conforming to the rest of the study, and with average linkage. Unlike the PCA plots, which show only the first two principal components, and thus do not capture the entire variability in the data, the dendrograms were computed based on all the dimensions of the gene expression

spaces. Nevertheless, the trends that they show are similar to those that can be observed in the PCA plots.

First, in both tissues, the LFD animals cluster together, and most of the dietary lifestyle intervention (DLI) animals cluster with them. The dietary intervention is so successful at reversing disease gene expression patterns that these animals become practically indistinguishable from the animals that were fed LFD throughout the experiment. In the case of adipose tissue, the T0901317 cluster with LFD and DLI animals, yet still forms a distinct subgroup, in contrast to LFD and DLI which are "mixed together". Yet, this occurs only in adipose; in the liver the T0901317 group is distinct from the LFD+DLI cluster.

Second, there exists large intra-group variability due to which many of the animals do not cluster with other animals from the same treatment group. Two notable exceptions occur: in the adipose tissue, rosiglitazone and pioglitazone cluster together and distinctly from other drugs. The same happens with fenofibrate and T0901317 in the liver. This seems to occur because each of these four compounds activates a key regulator that is expressed in the tissue in which it exerts considerable transcriptomic changes, whereas the other drugs either work through other mechanisms that have a subtler effect or exert their primary effect in tissues that were not examined in the current study, such as pancreatic β-cells (see Supplementary Table 2).

3. GSEA analysis

Gene set enrichment analysis (GSEA, (Subramanian *et al*, 2005)) was conducted to detect pathways that are enriched in genes that are either upregulated or downregulated in each treatment group compared with the HFD-16weeks group. Comparisons of the LFD and HFD-9weeks groups with the HFD-16weeks groups were made as well for the sake of completeness. The analysis was limited to gene sets from the collection of canonical KEGG pathways in the Molecular Signatures Database (MSigDB) v4.0 (accession: CP:KEGG; 186 gene sets in total) so as to retain statistical power in the face of multiple comparison. On the other hand, we emphasize that input to GSEA consisted of all the genes whose expression was measured, and not only the subset of top differentially-expressed genes that was used to define the gene expression space for the purpose of TDI computations (Methods).

Gene sets were downloaded from MSigDB (www.broadinstitute.org/gsea/msigdb; accessed July 2014) and translated from human gene identifiers to mouse gene identifiers using homology data from the Jackson laboratory (www.informatics.jax.org; accessed July 2014). GSEA software available from the Broad Institute (v2.1.0; www.broadinstitute.org/gsea/index.jsp; accessed July 2014) was run with default parameters. We note that phenotype permutation was used to assess the statistical significance of the enrichment scores. Phenotype permutation is more stringent and biologically reasonable than gene set permutation (Subramanian *et al*, 2005), and was therefore preferred despite the limits

it poses on statistical power in experiments with small number of samples in each group (our dataset typically has 8 animals per group).

Overall, the results agreed with prior expectations. Major hepatic and adipotic pathways were indeed modulated in the study animals by the drugs that are known to target them. Fenofibrate upregulated peroxisome proliferator-activated receptors (PPAR) signaling in the liver; fenofibrate, atorvastatin and T0901317 modulated hepatic fatty acid metabolism. Pioglitazone and rosiglitazone activated PPAR signaling and genes associated with fatty acid metabolism in white adipose. An exception to that was metformin, which did not alter any hepatic pathway in a statistically-significant way. This does not seem to stem from under-dosage because the dosage used was comparable to the one given in previous studies (250 mg/kg, 0.25% w/w), alleviated some of the clinical phenotypes of the disease (Radonjic *et al*, 2013), and significantly decreased the hepatic TDI compared with untreated HFD group (Supplementary Results 1). The indiscernibility of metformin's effects in GSEA analysis may thus stem from the lack of statistical power.

Interestingly, pro-inflammatory pathways were downregulated in adipose gene expression in the LFD and DLI groups compared with the HFD group, which accords with the importance of adipose-related inflammatory processes in HFD-induced pathologies (Wellen & Hotamisligil, 2003; Berg & Scherer, 2005). Nonetheless, inflammatory pathways were upregulated in the liver by T0901317, which is also apparent in direct inspection of the expression of known pro-inflammatory genes (Supplementary Results 5). The hepatic inflammatory response is probably associated with the deleterious physiological outcomes observed in T0901317 mice, most notably abnormal hepatomegaly (see main text in the results subsection "Non-restorative alterations to the gene expression are associated with unfavorable outcomes").

GSEA results also accord with observations that were reached in this study through other means: first, the dietary regimen seems particularly effective in inducing the opposite transcriptomic patterns than HFD. In both tissues there are multiple pathways which are altered between the LFD and the untreated HFD group due to HFD-feeding, and are altered in the opposite direction by DLI; the same reversal occurs in none or only in a handful of the drugs in each case, and only those that were shown to exert the most positive effect in the study animals. Second, taking the number of significantly altered gene sets as a proxy for the magnitude of the drug effect, we find that the drugs that had the most significant effects are the same ones that exert the largest effects as seen in the gene expression space (Supplementary Figure 12).

One result that we did not anticipate was the frequent occurrence of the KEGG_RIBOSOME gene set among the significantly altered ones in the liver. This gene set is upregulated in the liver by pioglitazone, rosiglitazone, fenofibrate, and T0901317 compared with the HFD group. Moreover, it is downregulated in the LFD and DLI group compared with the HFD

group, suggesting that this pathway's downregulation is a phenotype associated with HFD and rectified by DLI. The gene set is also upregulated in the animals fed HFD for 9 weeks compared with the ones fed HFD for 16 weeks, which may be interpreted as a sign that this pathway's downregulation should be associated with a late phase in the disease progression and as a marker for a severe form of the disease state. Indeed, it has been recently shown that HFD-feeding repressed liver ribosomal RNA transcription in both wildtype (C57BL6) mice fed HFD and in an obese mouse model (ob/ob) fed normal diet compared with wildtype mice fed a normal diet (Oie *et al*, 2014).

Another noteworthy effect occurs in the liver, where T0901317 downregulated the gene set KEGG_COMPLEMENT_AND_COAGULATION_CASCADES compared with the HFD group. A similar result was previously reported in a zebrafish study of T0901317's hepatic effects (Sukardi *et al*, 2012), suggesting that it is not accidental but rather concerns a conserved biological mechanism in the two species. In our data, rosiglitazone (yet not pioglitazone) had the opposite effect and significantly upregulated this gene set in the liver. The gene set was significantly downregulated in the adipose tissue of the LFD group, yet not in the livers of the LFD animals.

4. Alternative definitions of TDI

4.1. GSEA-based methods

We define the Transcritome Deviation Index as the Euclidean distance in the gene expression space between an animal and the mean of the healthy (low-fat diet) animals. Note that the gene expression space is defined through genes that are differentially expressed between the HFD and LFD groups, and are thus associated with the disease phenotypes. A different approach was taken by several previous studies (Lamb *et al*, 2006; Iorio *et al*, 2010; Sirota *et al*, 2011; Pacini *et al*, 2013) that sought inverse correlations between drug and disease profiles derived from gene expression data, and applied Gene Set Enrichment Analysis (GSEA) (Subramanian *et al*, 2005) towards that purpose. Briefly, these studies computed a score that is based on the Kolmogorov-Smirnov statistic and quantifies the extent by which genes that are up-regulated in the disease profile tend to be up-regulated in the treatment profile and, similarly, genes that are down-regulated in the disease profile tend to be down-regulated in the treatment profile. Negative scores occur when genes that are up-regulated by the disease tend to be down-regulated by the treatment, and vice-versa, and suggest that the drug might be effective in treating the disease. Following (Sirota *et al*, 2011), these score are denoted DDS (which stands for drug-disease-score, although here they are applied in the case of the non-pharmacological dietary intervention, and in an individual manner, see below).

TDIs and DDSs are thus two ways to quantify the success of a treatment to reverse gene expression patterns induced by the disease. Treatments that successfully act towards this goal should have both small (close to 0) TDIs and small ("very negative") DDSs compared with

unsuccessful treatments. Therefore, one expects TDIs and DDSs to be directly correlated; we verified that a strong correlation indeed exists.

DDSs were computed for each animal in the dataset studied here. There are minute differences between the ways the scores are computed in the various studies that used a GSEA-based approach; we followed (Sirota *et al*, 2011). As in the TDIs, a) DDSs were computed separately for the adipose and for the liver gene expression, and b) DDSs were computed for each individual animal, rather than for an entire treatment group. Thus, DDSs offer an alternative quantification for the tissue-specific reversal of the HFD gene expression patterns in a certain animal. As expected, TDIs and DDSs are highly correlated (Supplementary Figure 5; Pearson rho = 0.97, 0.96, p-values < 1.4e-38, 3.4e-62 for the adipose and liver tissues, respectively). On the other hand, the definition of TDIs allows a simple decomposition of the TDI into two orthogonal components: one that corresponds to disease reversal, and one which is associated with adverse outcomes (Supplementary Figure 13a). It is not as straightforward to do the same for GSEA-based scores, and therefore the definition of TDIs that is presented in the main text was chosen for the current study.

4.2. Genes mapped to multiple probes

A subtle choice in the definition of TDIs concerns the way probes that are mapped to multiple genes are handled. We opted for the most data-driven approach, and treated each probe as a separate feature, thus accommodating the possibility that a particular probe might be much more correlated with disease phenotypes than other probes mapped to the same gene. We verified, however, that our results do not depend on this choice. Similar results are obtained when all the probes that are associated with a particular gene are collapsed into a single feature.

5. Up-regulation of pro-inflammatory genes in the T0901317 treatment group

T0901317 and fenofibrate are associated with unfavorable physiological outcomes that are indicative of liver pathologies, and particularly with notable hepatomegaly (see main text and Supplementary Figure 14), as well as with large non-restorative alterations in the liver gene expression (Supplementary Figures 12a, 13b). We hypothesized that these unfavorable phenotypes are accompanied by hepatic inflammation (Reddy & Sambasiva Rao, 2006). Therefore, we tested whether 13 known inflammatory genes were up-regulated in the livers of mice treated with one of these drugs compared with untreated HFD mice (one-sided t-test; p-values were adjusted to multiple hypotheses by the Benjamini-Hochberg method; significance level was set at 5%). No significantly up-regulated genes were observed in the fenofibrate group. However, 6 out of the 13 tested genes were significantly up-regulated in the T0901317 (we stress that the comparison is with the untreated HFD group and not with the LFD group): MCP-1, CD86, EMR-1, ICAM-1, VCAM-1, and IL-1β. In addition, TNF-α was up-regulated, but not

in a statistically-significant manner (adjusted p-value = 0.11, unadjusted p-value = 0.036). The other 6 genes that were tested are: SELE, SELP, NOS-1, NOS-2, IL-6, IL-18.

Supplementary Figures

1. <u>Supplementary Figure 1: Transcriptome deviations indices</u>

(a) Liver and (b) adipose TDI distribution of the different experimental groups

(a)

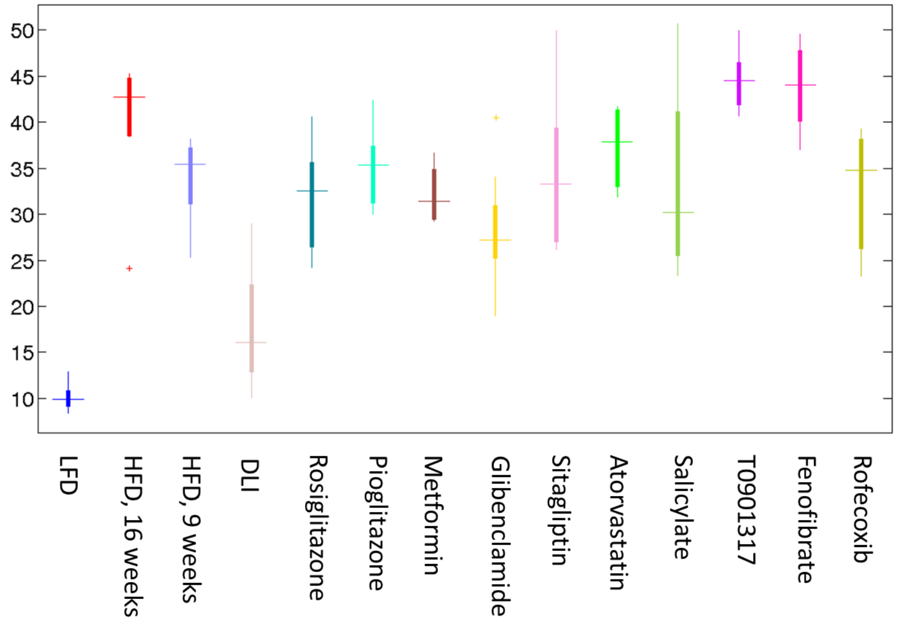

(b)

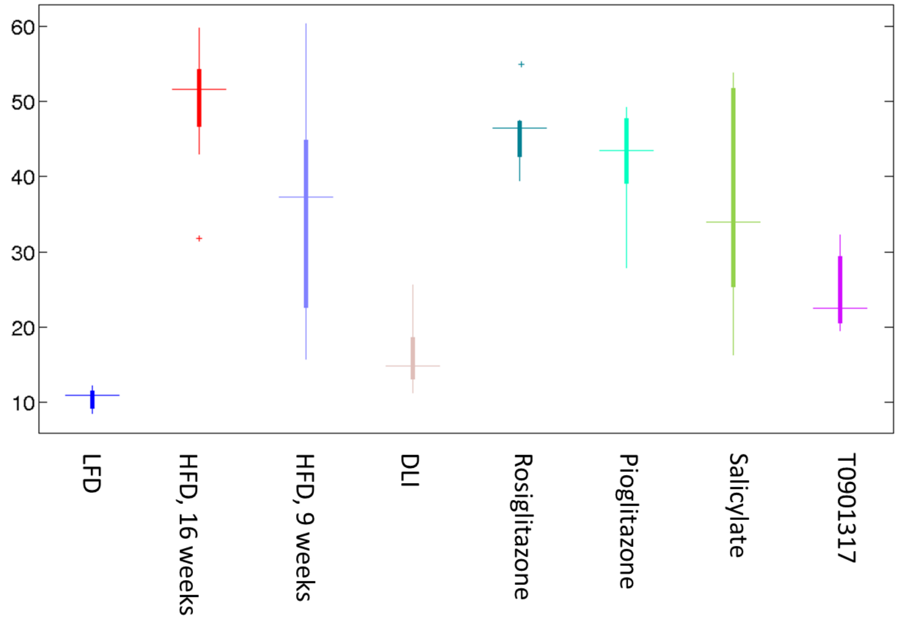

2. Supplementary Figure 2: Global physiological deviation indices

GPDI distribution of the different experimental groups

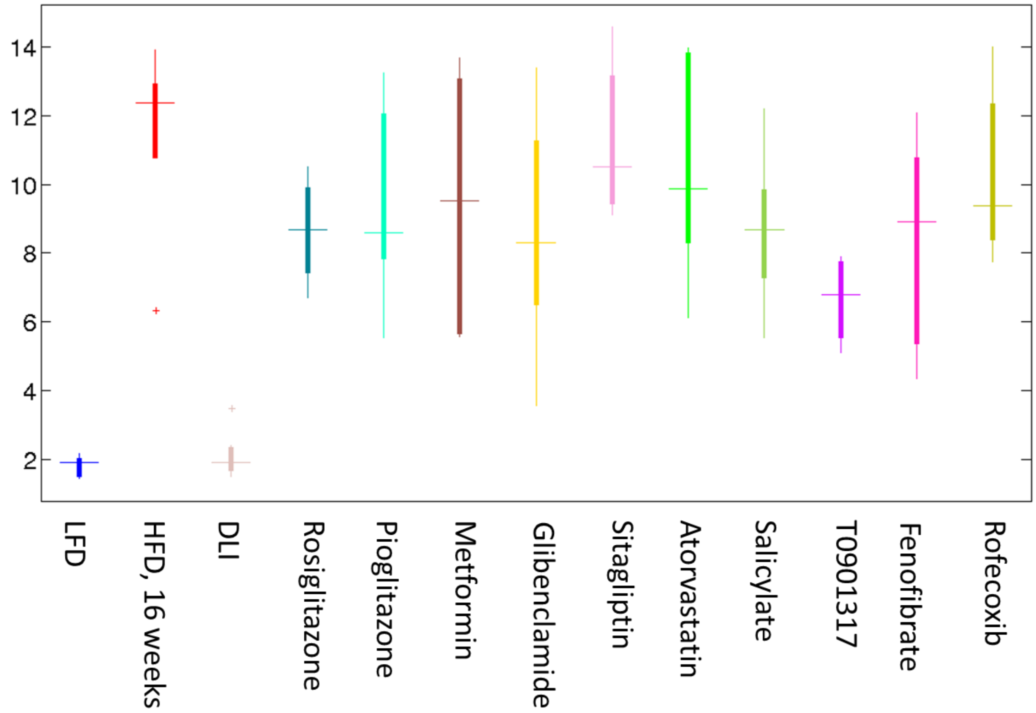

3. Supplementary Figure 3: Hierarchical clustering in the liver gene expression space

Dendrogram was built with Euclidean distances and average linkage. Each leaf in the dendrogram corresponds to one animal, and leaf labels denote the treatment group to which the animal belonged. Two clusters are highlighted. The first (blue, bottom part of the dendrogram) contains all the LFD animals, and 6 out of the 8 dietary intervention animals. The second (pink, upper part of the dendrogram) contains all the animals treated with the lipid-modulating drugs fenofibrate and T0901317. Both of which activate master transcription factor that are highly expressed in the liver. See Supplementary Results 2 and main text for details.

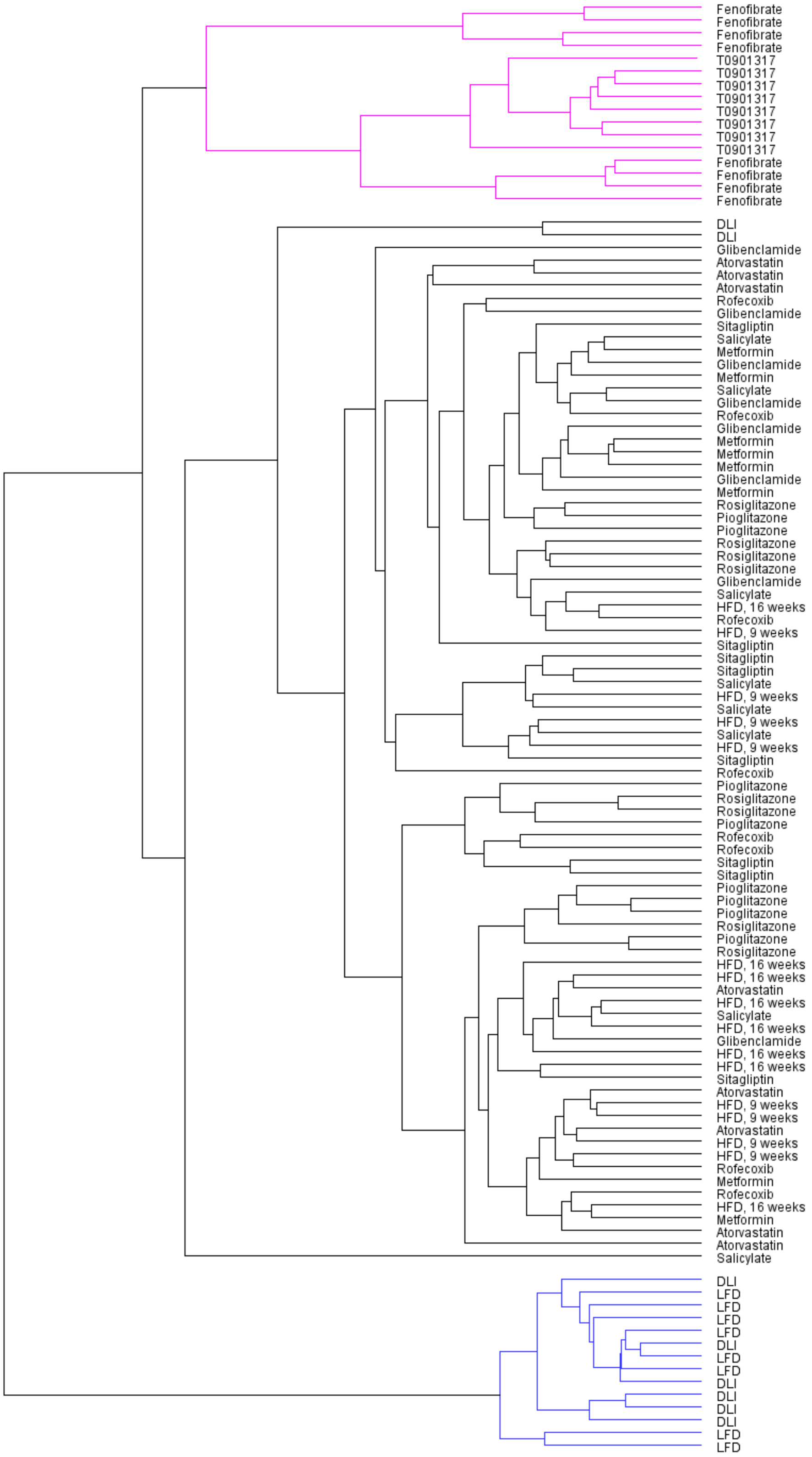
Fenofibrate
Fenofibrate
Fenofibrate
Fenofibrate
T0901317
T0901317
T0901317
T0901317
T0901317
T0901317
T0901317
T0901317
Fenofibrate
Fenofibrate
Fenofibrate
Fenofibrate
DLI
DLI
Glibenclamide
Atorvastatin
Atorvastatin
Atorvastatin
Rofecoxib
Glibenclamide
Sitagliptin
Salicylate
Metformin
Glibenclamide
Metformin
Salicylate
Glibenclamide
Rofecoxib
Glibenclamide
Metformin
Metformin
Metformin
Glibenclamide
Metformin
Rosiglitazone
Pioglitazone
Pioglitazone
Rosiglitazone
Rosiglitazone
Rosiglitazone
Glibenclamide
Salicylate
HFD, 16 weeks
Rofecoxib
HFD, 9 weeks
Sitagliptin
Sitagliptin
Sitagliptin
Salicylate
HFD, 9 weeks
Salicylate
HFD, 9 weeks
Salicylate
HFD, 9 weeks
Sitagliptin
Rofecoxib
Pioglitazone
Rosiglitazone
Rosiglitazone
Pioglitazone
Rofecoxib
Rofecoxib
Sitagliptin
Sitagliptin
Pioglitazone
Pioglitazone
Pioglitazone
Rosiglitazone
Pioglitazone
Rosiglitazone
HFD, 16 weeks
HFD, 16 weeks
Atorvastatin
HFD, 16 weeks
Salicylate
HFD, 16 weeks
Glibenclamide
HFD, 16 weeks
HFD, 16 weeks
Sitagliptin
Atorvastatin
HFD, 9 weeks
HFD, 9 weeks
Atorvastatin
HFD, 9 weeks
HFD, 9 weeks
Rofecoxib
Metformin
Rofecoxib
HFD, 16 weeks
Metformin
Atorvastatin
Atorvastatin
Salicylate
DLI
LFD
LFD
LFD
LFD
DLI
LFD
LFD
DLI
DLI
DLI
DLI
LFD
LFD

4. Supplementary Figure 4: Hierarchical clustering in the adipose gene expression space

Dendrogram was built with Euclidean distances and average linkage. Each leaf in the dendrogram corresponds to one animal, and leaf labels denote the treatment group to which the animal belonged. Two clusters are highlighted. The first (blue, upper part of the dendrogram) contains all the LFD animals, most of the dietary intervention animals, and all the animals treated with T0901317. The second (pink, bottom part of the dendrogram) contains almost all the animals treated with the thiazolidinediones (TZDs) rosiglitazone and pioglitazone. These two drugs activate a master transcription factor that is highly expressed in adipose. See Supplementary Results 2 and main text for details.

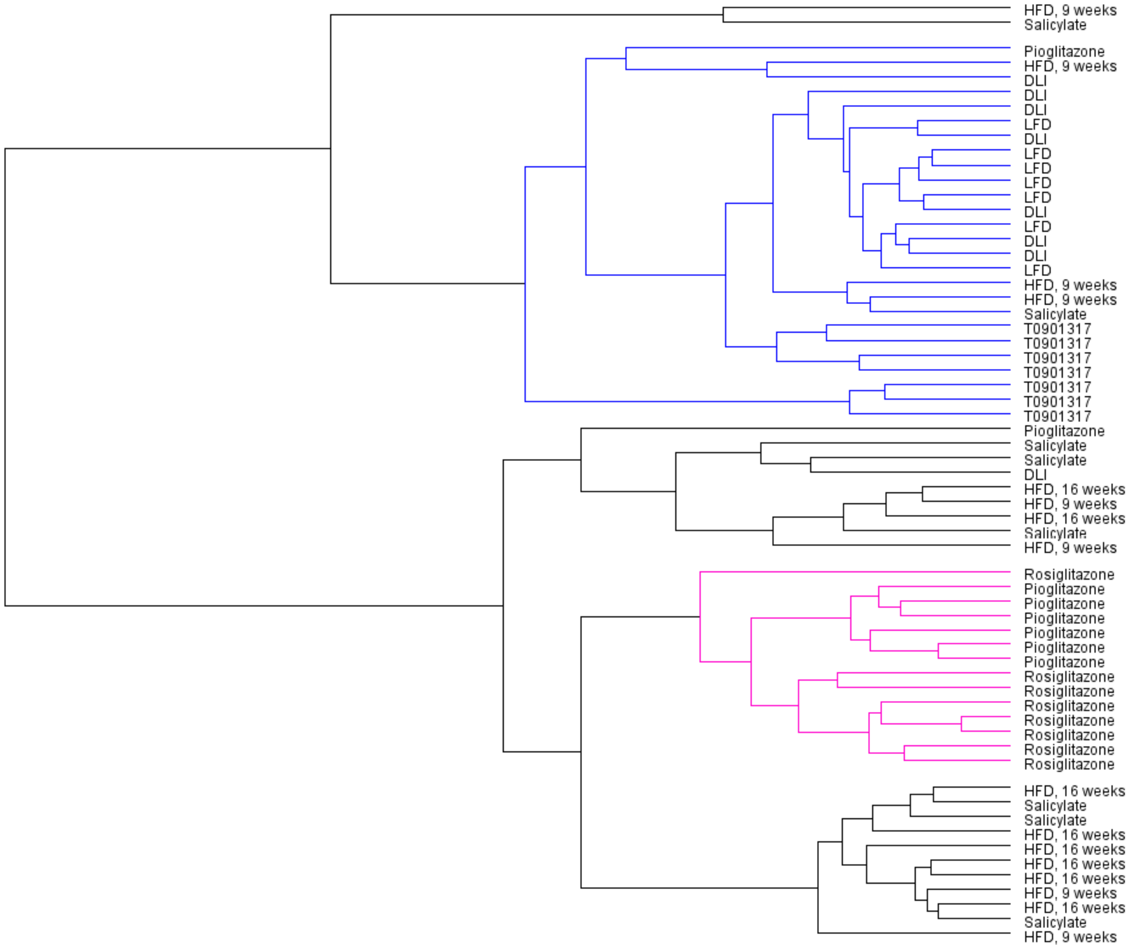

5. Supplementary Figure 5: Correlation between GSEA-based scores and TDIs

Scatter plots of DDSs, which are GSEA-based scores (see Supplementary Results 4.1) that measures a drug's ability to reverse the transcriptomic patters of the disease, and the Transcriptome Deviation Indices (TDIs), which quantify the same ability via other means. We find that the two are highly correlated both when computed for (a) liver and (b) white adipose gene expression. X and y axes are TDIs and DDSs, respectively. Each dot represents one animal, color-coded according to its treatment group as in the rest of the study. Pearson correlation coefficients and their corresponding p-values are given for each tissue.

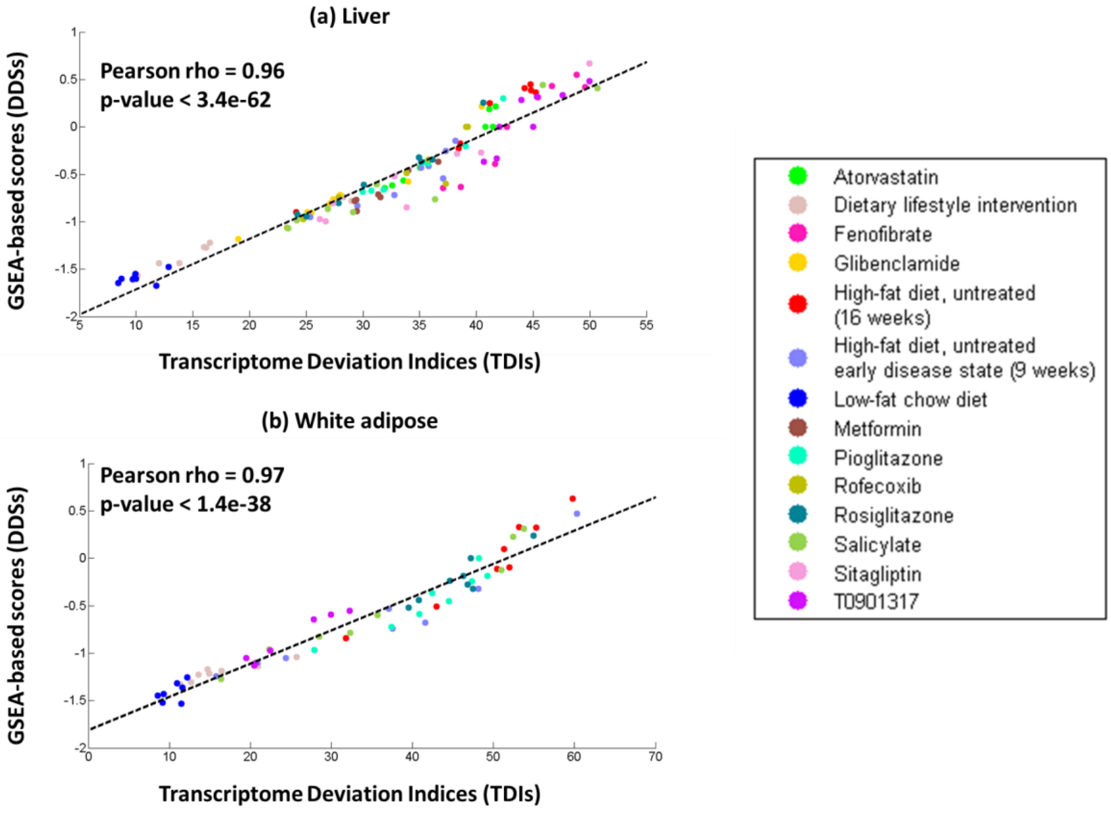

6. Supplementary Figure 6: Spearman correlations of TDI, GPDI, and MDI

This figure parallels Figure 2 of the main text, except that it presents Spearman correlations and their respective p-values instead of Pearson correlations. Accordingly, the x and y axes of each panel present the ranked values rather than the actual values. For example, the x coordinates of panel (a) are the rank of each animal's adipose TDI with respect to all the animals for which adipose TDI was available.

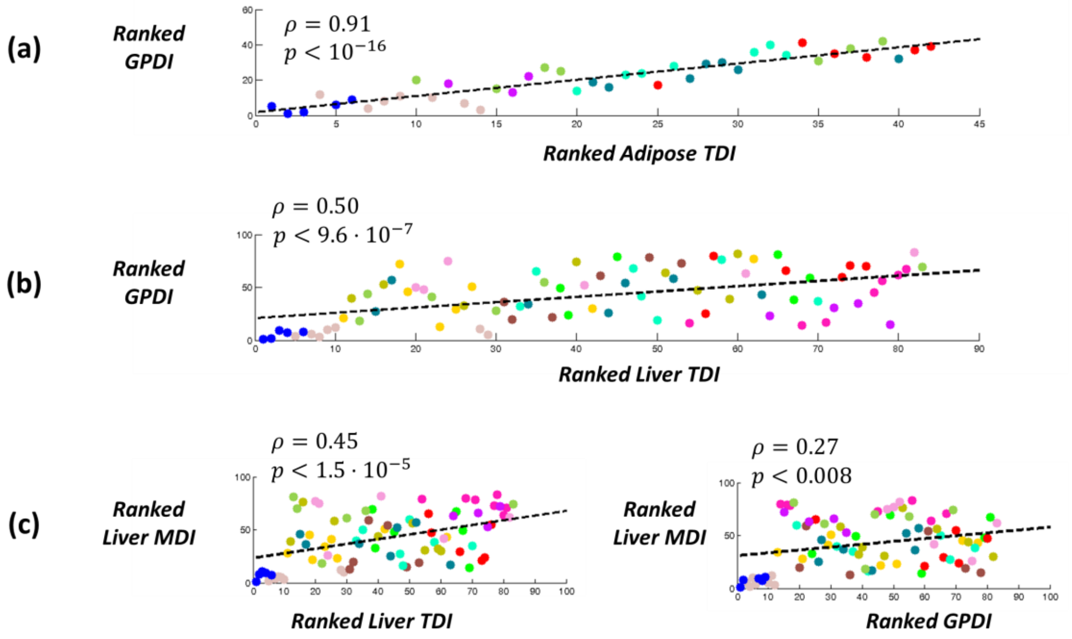

7. Supplementary Figure 7: Liver metabolome space

First two principal components of the liver metabolome space. Each dot represents one animal; color codes denote the different experimental groups. The dashed arrow connects the HFD centroid (yellow square) to the LFD centroid (yellow triangle), and denotes the direction of a reversal of the gene expression or physiological state back to the norm.

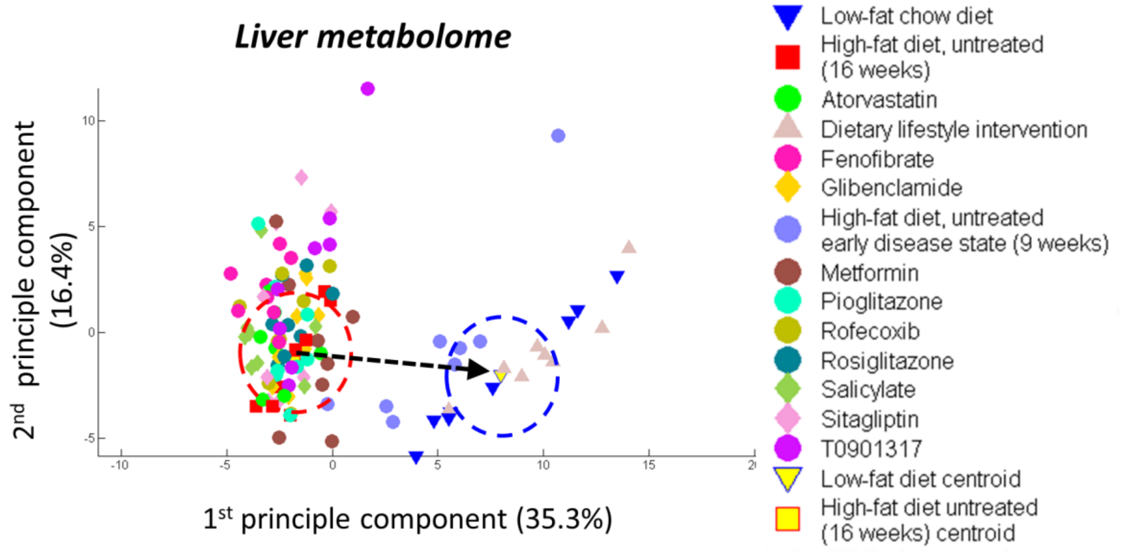

8. Supplementary Figure 8: White adipose TDI correlation with individual PDIs

Each panel presents the ranked PDI values of a particular physiological marker (y-axis) as a function of the ranked adipose TDI (x-axis). Each dot represents one animal, color-codes denote the different experimental groups. The dashed lines are linear regression lines. Refer to Supplementary Table 1 for complete details concerning the physiological markers. The Spearman correlations values and their respective p-values are given in Supplementary Table 7.

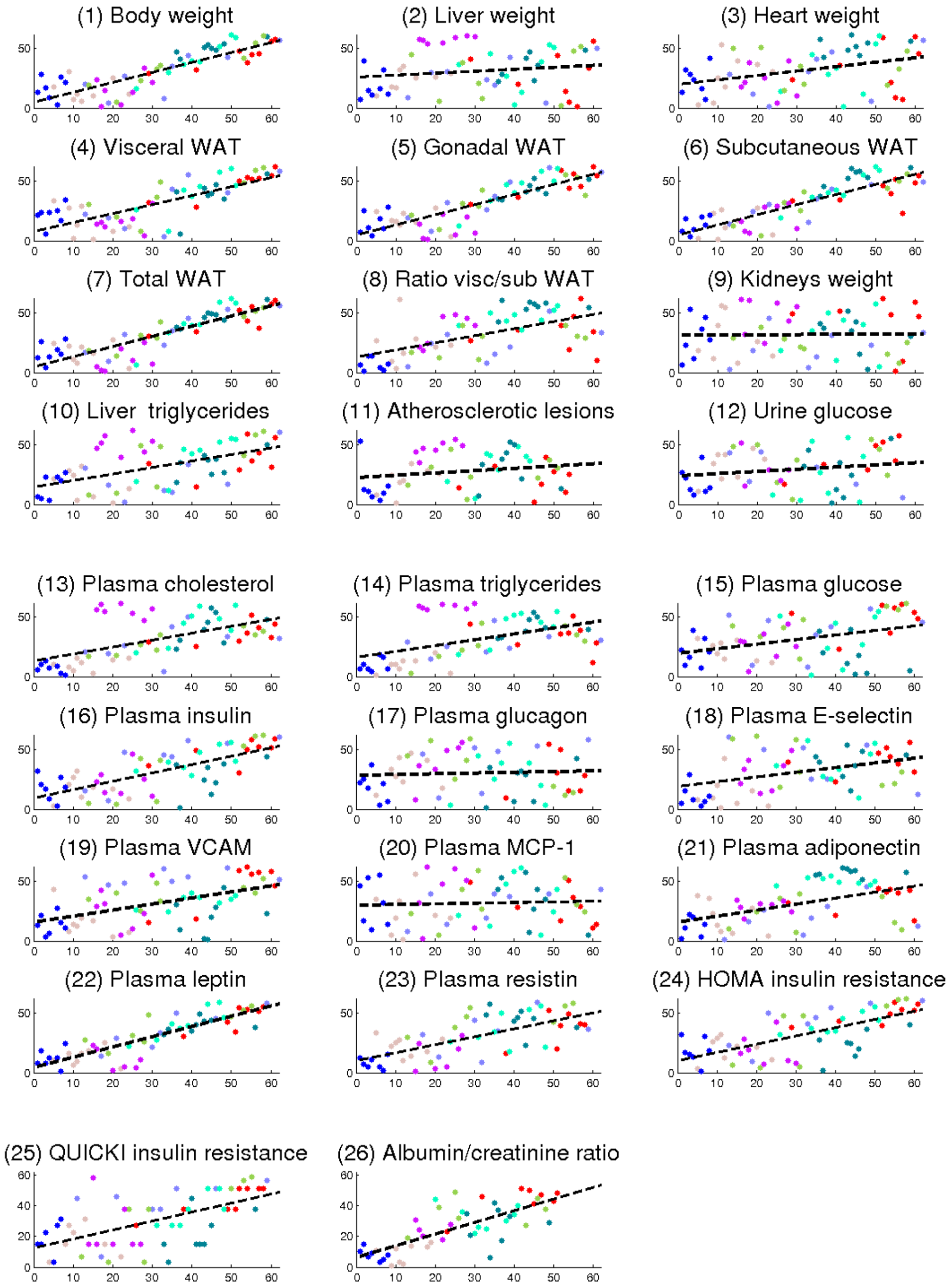

9. Supplementary Figure 9: Liver TDI correlation with individual PDIs

Each panel presents the ranked PDI values of a particular physiological marker (y-axis) as a function of the ranked liver TDI (x-axis). Each dot represents one animal, color-codes denote the different experimental groups. The dashed lines are linear regression lines. Refer to Supplementary Table 1 for complete details concerning the physiological markers. The Spearman correlations values and their respective p-values are given in Supplementary Table 7.

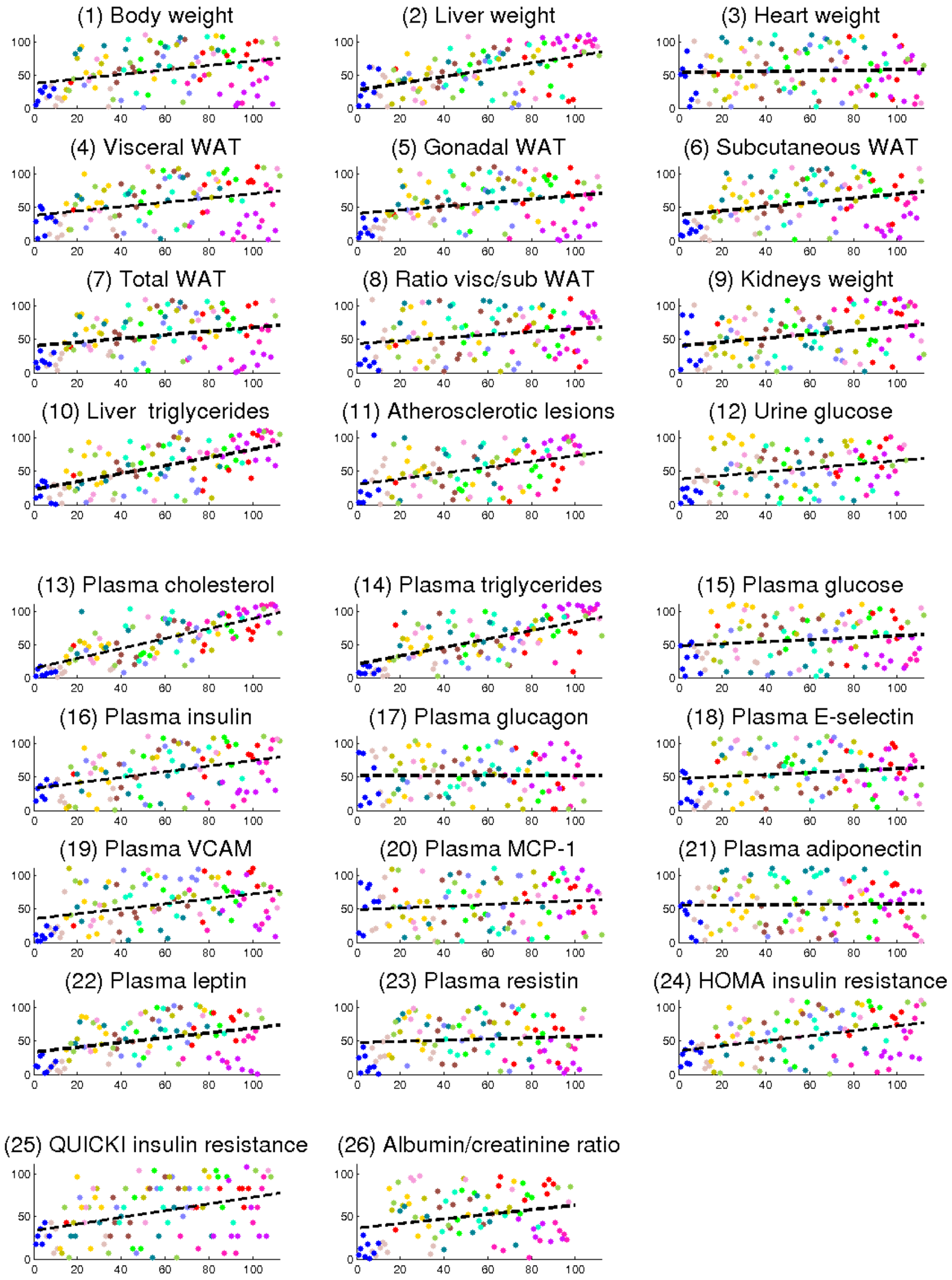

10. <u>Supplementary Figure 10: Liver MDI correlation with individual PDIs</u>

Deviations from the baseline liver metabolome (MDI) are correlated with deviations from the normal physiology (PDIs) in markers that are known to be associated with liver functions. Bar lengths represent the Spearman correlations between the hepatic MDI and PDIs of the measured 26 physiological markers. The liver has a central role in lipid metabolism, reflected in the relatively high correlations of its MDI and the physiological markers at the bottom part of the figure. WAT stands for white adipose tissue, ratio visc/sub WAT for ratio of visceral to subcutaneous WAT. Asterisks mark statistically-significant correlations (using the Benjamini-Hochberg correction for multiple hypotheses testing with a 5% FDR level). One marker (plasma MCP-1) had a negative correlation of -0.11 with the liver MDI, which is not shown in this figure.

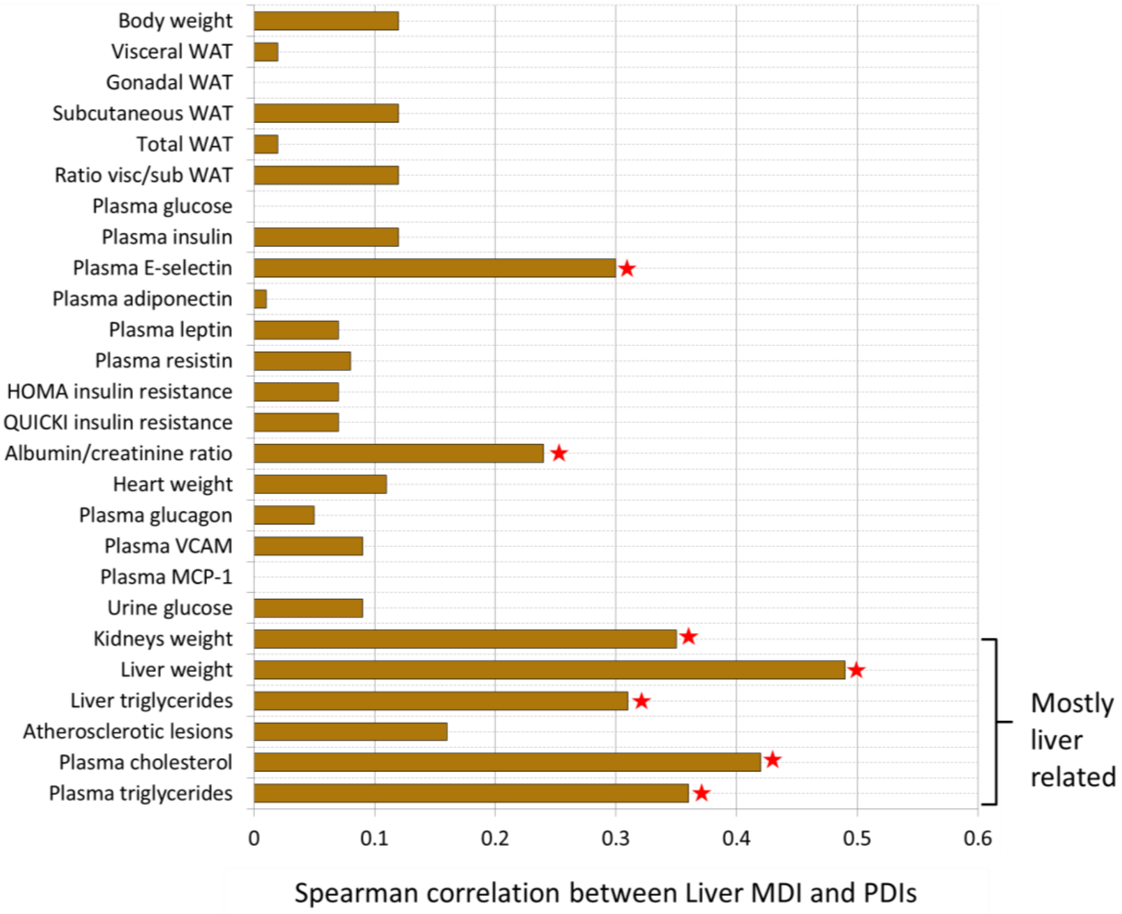

11. Supplementary Figure 11: Liver MDI correlation with individual PDIs (scatter plots)

Each panel presents the ranked PDI values of a particular physiological marker (y-axis) as a function of the ranked liver MDI (x-axis). Each dot represents one animal, color-codes denote the different experimental groups. The dashed lines are linear regression lines. Refer to Supplementary Table 1 for complete details concerning the physiological markers. The Spearman correlations values and their respective p-values are given in Supplementary Table 8.

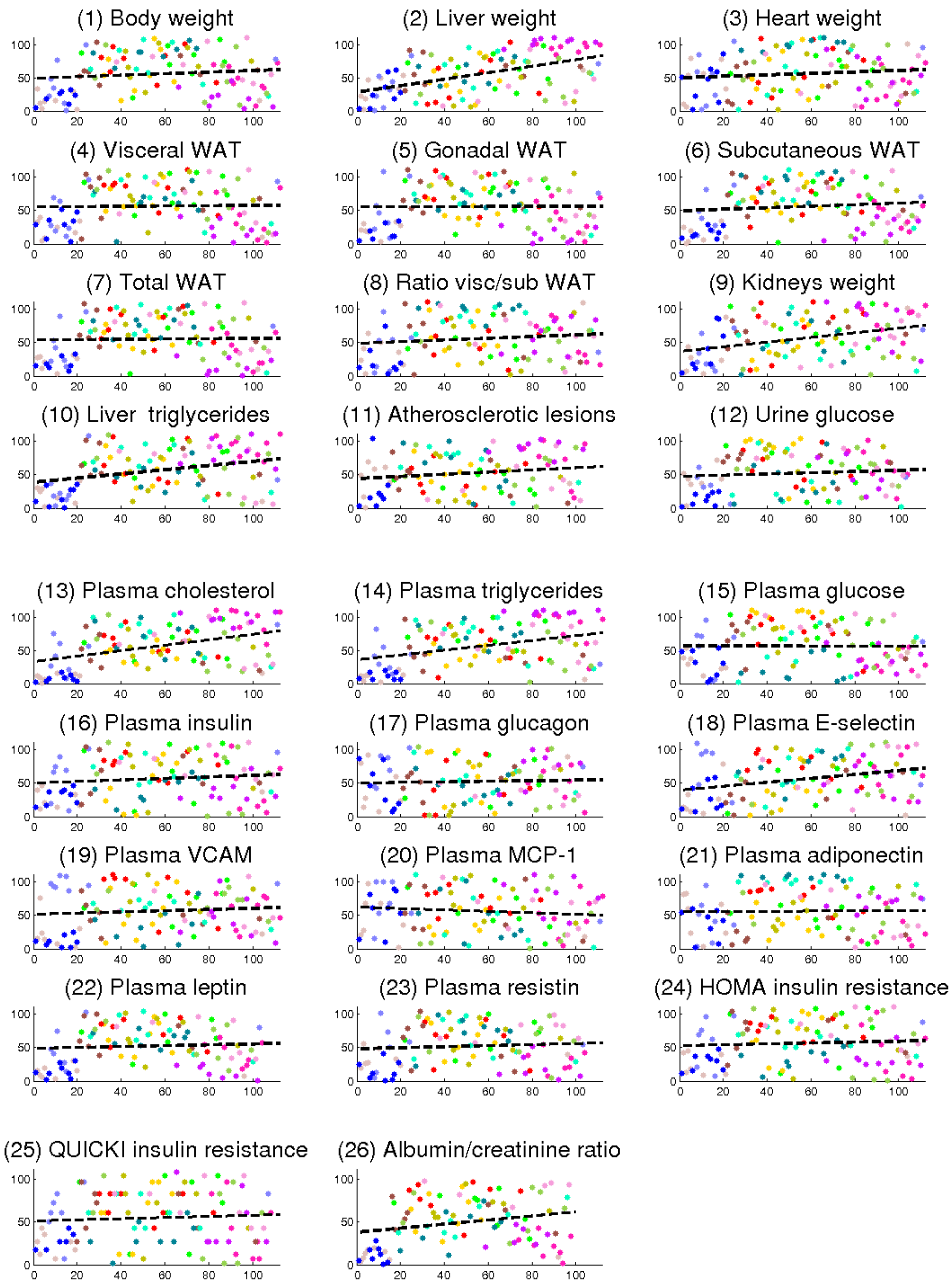

12. Supplementary Figure 12: Drugs that induce major non-restorative gene expression alterations

This figure reproduces Figure 1a-b from the main text, highlighting the four "outlier" drugs, Fenofibrate (pink) and T0901317 (purple) in the liver, and the two thiazolidinediones rosiglitazone (dark cyan) and pioglitazone (light cyan). These drugs induce major gene expression changes that are not congruent with reversal of the disease transcriptomic patterns. The direction of reversal is denoted by the dashed arrow that connects the HFD centroid (yellow square, circled in red) to the LFD centroid (yellow triangle, circled in blue).

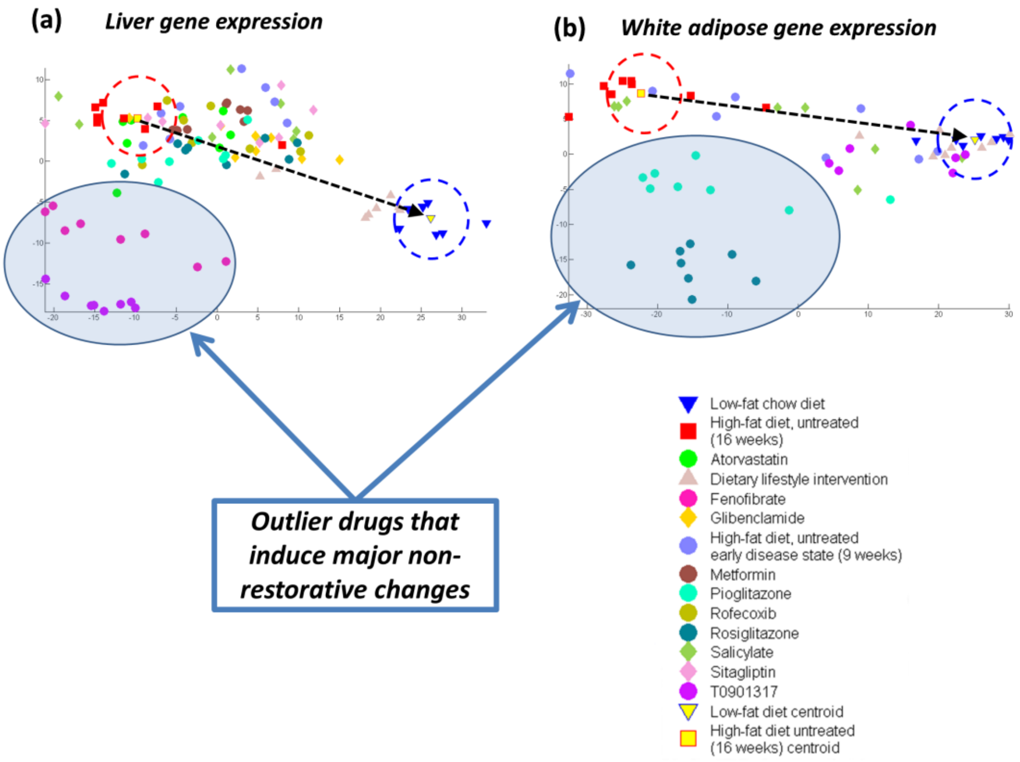

13. Supplementary Figure 13: Non-restorative gene expression alterations are associated with unfavorable physiological outcomes

(a) A schematic illustration demonstrating the definition of non-restorative gene expression alterations. The gene expression space of a particular tissue is shown. Blue, red, and green markers represent LFD, untreated HFD, and treated HFD subjects, respectively. The dashed axis goes from the HFD mean to the LFD mean (yellow square and triangle, respectively). The treatment effects on each subject can be decomposed into two components: (1) reversal of the disease-induced gene expression patterns, which operates along the direction of the axis that goes from the HFD mean to the LFD mean, and (2) additional alterations which are orthogonal to that axis and hence incongruent with the healthy (LFD) state (Methods). We term the latter "non-restorative alterations" and hypothesize that they are associated with unfavorable physiological outcomes. (b-c) The distributions of the magnitudes of non-restorative alterations to the (b) liver and (c) white adipose gene expression induced by the various drugs. In (b) experimental groups are ordered from left to right in the same order that they appear in the legend; in (c) the groups are ordered from left to right as follows: DLI, pioglitazone, rosiglitazone, salicylate, T0901317. Evidently, four drugs induce the largest non-restorative alterations: fenofibrate and T0901317 in the liver and rosiglitazone and pioglitazone in white adipose (compare Figure 1a-b and Supplementary Figure 12). (d) a schematic illustration of the method employed to detect unfavorable outcomes in the physiological data available in the studied animals. Intuitively, a marker was considered as manifesting an unfavorable outcome of a certain treatment if its values in the treated animals were even farther from the LFD baseline than its values in the untreated HFD animals. This is exemplified in the illustration: while the untreated HFD animals (red bar) have higher blood triglycerides levels than the LFD animals (blue bar), the animals treated with a certain bar (green bar) have even higher blood triglycerides than the untreated HFD animals. Hence, the marker represents an unfavorable outcome of the treatment in this case. See the main text for a complete definition that also quantifies the statistical significance of the observation.

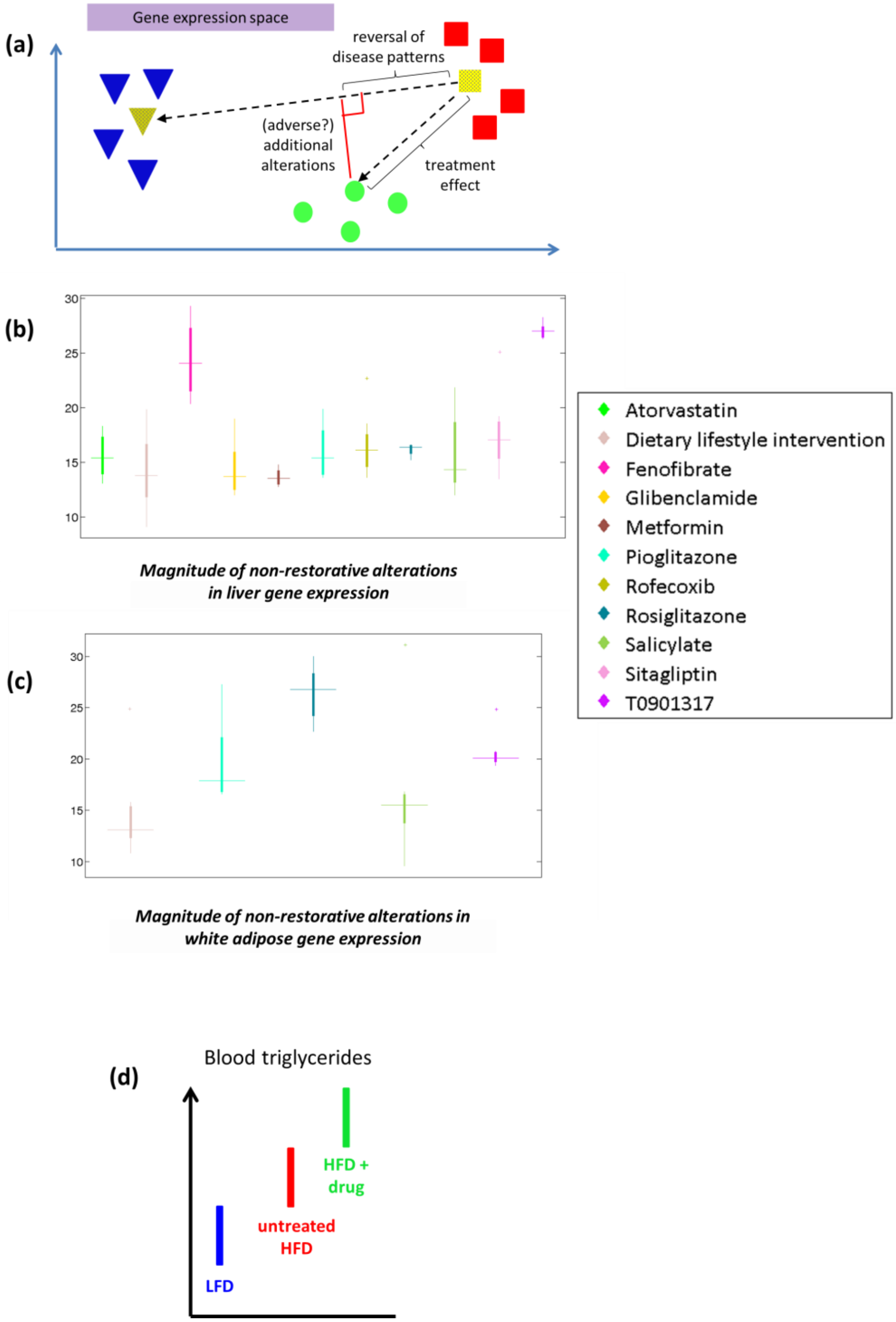
(a)
Gene expression space
reversal of
disease patterns
(adverse?)
additional
alterations
treatment
effect
(b)
30
25
20
15
10
Magnitude of non-restorative alterations
in liver gene expression
Atorvastatin
Dietary lifestyle intervention
Fenofibrate
Glibenclamide
Metformin
Pioglitazone
Rofecoxib
Rosiglitazone
Salicylate
Sitagliptin
T0901317
(c)
30
25
20
15
10
Magnitude of non-restorative alterations in
white adipose gene expression
(d)
Blood triglycerides
HFD +
drug
untreated
HFD
LFD

14. <u>Supplementary Figure 14: Unfavorable drug outcomes in physiological marker data</u>

This figure highlights the statistically-significant unfavorable physiological outcomes that were ascribed to particular drugs. Each panel presents the distribution of one physiological marker, with each bar representing one experimental group, color-coded as in the rest of the paper. Refer to Supplementary Table 1 for details concerning the measured markers, their units etc. A marker was considered as an unfavorable outcome of a certain treatment if its values in the treated animals were even farther from the baseline than its values in the untreated HFD animals in a statistically-significant manner (see main text for details). The figure shows all the statistically-significant associations of an unfavorable physiological outcome and a drug found in the data (i.e., the panels correspond to all the markers, interpreted as unfavorable outcomes, for which a statistically-significant association with at least one drug is detected; boxes shown in the panel correspond to the all the drugs which were associated with this unfavorable outcome). Note that an exception was made in the case of fenofibrate, which was not associated with elevated plasma triglycerides and atherosclerotic lesion area as unfavorable outcomes in a statistically significant manner; it is shown in those panels only for completeness of the presentation. One LFD outlier in the right panel of the middle row had a value of 1.6, but was clipped to a value of 3 (dashed line) for the sake of visualization.

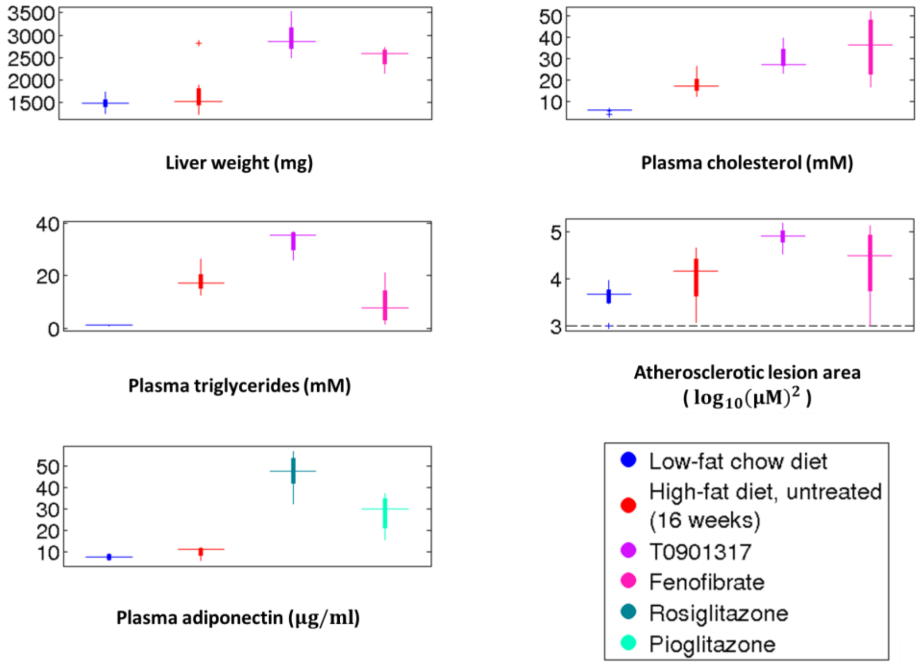

Supplementary Tables

1. Supplementary Table 1: List of physiological markers measured in the study animals

WAT = White Adipose Tissue.
The termination column indicates the time point at which these markers were measured: 9 weeks for the HFD-9wks group, and 15 or 16 weeks for the other groups.

	**Physiological marker**	**Units**	**Fasted / non-fasted**	**Termination**
1	Body weight	g	non-fasted	t=16/t=9
2	Liver weight	mg	non-fasted	t=16/t=9
3	Heart weight	mg	non-fasted	t=16/t=9
4	Visceral WAT	mg	non-fasted	t=16/t=9
5	Gonadal WAT	mg	non-fasted	t=16/t=9
6	Subcutaneous WAT	mg	non-fasted	t=16/t=9
7	Total WAT (visceral + gonadal + subcutaneous)	mg	non-fasted	t=16/t=9
8	Ratio visceral / subcutaneous WAT	mg/mg	non-fasted	t=16/t=9
9	Kidneys weight (total both kidneys)	mg	non-fasted	t=16/t=9
10	Liver triglycerides	mmol/mg liver	non-fasted	t=16/t=9
11	Atherosclerotic lesion area (*)	$\log_{10}(um^2)$	non-fasted	t=16/t=9
12	Urine glucose	mM	non-fasted	t=15/t=9
13	Plasma cholesterol	mM	fasted	t=15/t=9
14	Plasma triglycerides	mM	fasted	t=15/t=9
15	Plasma glucose	mM	fasted	t=15/t=9
16	Plasma insulin	ng/ml	fasted	t=15/t=9
17	Plasma glucagon	pg/ml	non-fasted	t=16/t=9
18	Plasma E-selectin	ng/ml	fasted	t=15/t=9
19	Plasma VCAM	ug/ml	fasted	t=15/t=9
20	Plasma MCP-1	pg/ml	non-fasted	t=16/t=9
21	Plasma adiponectin	ug/ml	fasted	t=15/t=9
22	Plasma leptin	ng/ml	non-fasted	t=16/t=9
23	Plasma resistin	ng/ml	non-fasted	t=16/t=9
24	HOMA insulin resistance	(**)	fasted	t=15/t=9
25	QUICKI insulin resistance	(***)	fasted	t=15/t=9
26	ACR (urine albumin /	ug/mg	fasted	t=15/t=9

	creatinine ratio)			

(*) The marker was log-transformed because it was highly skewed, and followed an approximately normal distribution much more closely after taking the log. Note that (Radonjic *et al*, 2013) did not carry a similar transformation.

(**) computed as $(fasting\ glucose\ \times fasting\ insulin)/22.5$, fasting glucose in mmol/l and fasting insulin in mU/l (Matthews *et al*, 1985).

(***) computed as $1/[\log(fasting\ insulin) + \log(fasting\ glucose)]$, fasting insulin in uU/ml and fasting glucose in mg/dl (Katz *et al*, 2000).

2. Supplementary Table 2: Drug mechanism of action

The mechanism of action of drugs studied in this paper. Data is based on Drugbank (Law *et al*, 2013) (accessed July 2014) except where otherwise noted.

Drug	***Mechanism of action***
metformin	Metformin's mechanism of action is two-fold, inhibiting liver glucose production, and additionally augmenting peripheral glucose uptake, mainly in muscles. These effects are believed to be partly mediated by activation of liver kinase B1 (LKB-1) (Shaw *et al*, 2005), which in turn regulates 5' adenosine monophosphatase-activated protein kinase (AMPK), a key sensor of cellular metabolism and energetics. Nonetheless, metformin has been reported to improve glucose tolerance in liver AMPK-deficient mice (Foretz *et al*, 2010), which suggests that part of its effects occurs through AMPK-independent pathways (Rena *et al*, 2013).
glibenclamide	Glibenclamide is a second generation sulfonylurea, which stimulate insulin secretion by pancreatic β cells. Sulfonylureas bind to a sulfonylurea receptor that is associated with inward rectifying adenosine triphosphate (ATP)-sensitive potassium channels in β cells. Binding of a sulfonylurea inhibits the efflux of potassium ions through the channels and results in depolarization that opens voltage-gated calcium channels. This leads to calcium influx and to the release of preformed insulin (Nolte Kennedy, 2012).
sitagliptin	Sitagliptin inhibits the enzyme dipeptidyl peptidase-4 (DPP-4), which degrades the incretins glucagon-like peptide-1 (GLP-1) and glucose-dependent insulinotropic peptide (GIP). Incretins act on pancreatic β-cells to enhance glucose-dependent insulin secretion. Therefore, suppressing their degradation by DPP-4 improves glycemic control (Waget *et al*, 2011; Mudaliar & Henry, 2012).
rosiglitazone<br>pioglitazone	Pioglitazone and rosiglitazone are thiazolidinediones (TZDs), which exert their antidiabetic effects through activation of the gamma isoform of the peroxisome proliferator-activated receptor (PPARγ), a transcription factor that is highly expressed in adipose tissue, and is known to be a key regulator of adipogenesis and insulin sensitivity (Escher *et al*, 2001; Larsen *et al*, 2003; Evans *et al*, 2004; Vasudevan & Balasubramanyam, 2004; Poulsen *et al*, 2012; Ahmadian *et al*, 2013). Activation of PPARγ receptors regulates the transcription of insulin-responsive genes involved in the control of glucose production, transport and utilization. Thus, TZDs improve glycemic control in type 2 diabetic patients through insulin sensitization, rather than increased insulin secretion by pancreatic β cells (Soccio *et al*, 2014).
fenofibrate	The chief mode of action of fenofibrate is binding to PPARα, a transcription factor that is highly expressed in the liver (as well is in brown, but not white, adipose cells) (Escher *et al*, 2001; Evans *et al*, 2004; Oosterveer *et al*, 2009; Poulsen *et al*, 2012). Upon its activation PPARα heterodimerizes with retinoid X receptor (RXR); the heterodimers

	recognize specific PPARα response elements and modulate the expression of genes responsible for fatty acids and cholesterol metabolism (Staels *et al*, 1998). The decrease in plasma triglycerides induced by fibrates has been attributed to an inhibition of the synthesis and secretion of VLDL by the liver and increased degradation of triglyceride-rich lipoproteins through the expression of lipoprotein lipase and a decreased expression of apolipoprotein CIII (Forcheron *et al*, 2002).
T0901317	T0901317 is a synthetic Liver X Receptor (LXR) agonist. LXR has two isoforms, one of them (LXRβ) is ubiquitously expressed, whereas the other (LXRα) is restricted to particular tissues, including the liver. LXRs regulates lipid and cholesterol metabolism and also have anti-inflammatory properties (Schultz *et al*, 2000; Steffensen & Gustafsson, 2004; Ulven *et al*, 2005; Zhao & Dahlman-Wright, 2010). T0901317 has been found unsuitable for clinical use due to its pleotropic effects, but LXRs continue to be studied as an attractive drug targets (Jakobsson *et al*, 2012; Hong & Tontonoz, 2014).
atorvastatin	Atorvastatin lowers LDL cholesterol by inhibiting hydroxymethylglutaryl-coenzyme A (HMG-CoA) reductase, which catalyzes the conversion of HMG-CoA to mevalonate in the cholesterol biosynthesis pathway. Ample evidence support that statins' protective cardiovascular effects is not restricted to cholesterol metabolism but may also be related to their anti-inflammatory properties (Tousoulis *et al*, 2014).
salicylate	Salicylates are anti-inflammatory compounds that inhibit the activity of both types of cyclooxygenase (COX-1 and COX-2) and thus suppress platelet thromboxane synthesis. The artificial derivative acetylsalicylic acid, better known as aspirin, is broadly used to prevent atherosclerotic complications, most importantly myocardial infarction and ischemic stroke (Awtry & Loscalzo, 2000; Campbell *et al*, 2007; American Diabetes Association, 2013). Aspirin effectively inhibits platelet aggregation, yet this effect is partly mediated through its acetyl group (Furst *et al*, 2012; Steinberg *et al*, 2013). There may also be other mechanisms through which salicylates exert their favorable effects, such as inhibition of the pro-inflammatory κ-light-chain-enhancer of activated B cells (NF-κB) signaling pathway ((Kopp & Ghosh, 1994) but see also (Frantz *et al*, 1995; Steinberg *et al*, 2013)), and activation of AMPK (Hawley *et al*, 2012; Steinberg *et al*, 2013) that is also a target of metformin (see above). Notably, it has long been observed that salicylates have positive outcomes in in diabetic patients (Williamson, 1901; Gilgore, 1960; Gilgore & Rupp, 1962; Baron, 1982; Hundal *et al*, 2002; Shoelson, 2002; Goldfine *et al*, 2013).
rofecoxib	Rofecoxib is a selective cyclooxygenase-2 (COX-2) inhibitor, which has been withdrawn in 2004 worldwide by Merck & Co, due to an increased risk of cardiovascular events (Praticò & Dogné, 2005).

3. Supplementary Table 3: GSEA analysis of the liver transcriptome

Gene set enrichment analysis (GSEA, (Subramanian *et al*, 2005)) of the effects of the pharmacological and dietary interventions was conducted on the hepatic transcriptome. The analysis sought KEGG gene sets that were enriched with either upregulated or downregulated genes when comparing the treated animals with the untreated HFD-16weeks group. Comparisons of the LFD and HFD-9weeks groups with the HFD-16weeks groups were made as well for the sake of completeness. The table lists all the KEGG gene sets that were enriched at FDR < 25%. See Supplementary Results 3 for full details of the analysis.

Table columns:

- Direction:
  - up = upregulated in the treatment group compared with the HFD-16weeks group.
  - down = downregulated in the treatment group compared with the HFD-16weeks group.
- Gene set size: number of genes in the gene set
- ES, NES: Enrichment Score and Normalized Enrichment Score, respectively. See (Subramanian *et al*, 2005).

Gene set	*Direction*	*Gene set size*	*ES*	*NES*	*Nominal p-value*	*FDR q-value*
**Metformin**						
No enriched gene sets						
**Glibenclamide**						
No enriched gene sets						
**Sitagliptin**						
No enriched gene sets						
**Rosiglitazone**						
KEGG_RIBOSOME	up	53	-0.52	-2.28	0	0.001
KEGG_COMPLEMENT_AND_COAGULATION_CASCADES	up	67	-0.46	-1.86	0	0.036
**Pioglitazone**						
KEGG_MISMATCH_REPAIR	down	22	0.61	1.91	0.002	0.051
KEGG_RIBOSOME	up	53	-0.46	-1.97	0	0.015
KEGG_FATTY_ACID_METABOLISM	up	38	-0.47	-1.67	0.024	0.247
KEGG_HISTIDINE_METABOLISM	up	24	-0.52	-1.64	0.006	0.234
KEGG_PPAR_SIGNALING_PATHWAY	up	65	-0.44	-1.63	0.004	0.187
**Fenofibrate**						
KEGG_RIBOSOME	up	53	-0.58	-2.23	0	0
KEGG_PEROXISOME	up	72	-0.61	-1.99	0	0.007
KEGG_FATTY_ACID_METABOLISM	up	38	-0.77	-1.88	0	0.022

KEGG_PPAR_SIGNALING_PATHWAY	up	65	-0.75	-1.86	0	0.018
KEGG_ALZHEIMERS_DISEASE	up	129	-0.4	-1.86	0.002	0.015
KEGG_OXIDATIVE_PHOSPHORYLATION	up	94	-0.47	-1.82	0.018	0.022
KEGG_PARKINSONS_DISEASE	up	92	-0.45	-1.73	0.014	0.05
KEGG_VALINE_LEUCINE_AND_ISOLEUCINE_DEGRADATION	up	41	-0.51	-1.73	0.002	0.044
KEGG_PROPANOATE_METABOLISM	up	27	-0.61	-1.68	0.013	0.07
KEGG_GLYCEROPHOSPHOLIPID_METABOLISM	up	67	-0.46	-1.64	0.012	0.102
KEGG_GLYCEROLIPID_METABOLISM	up	42	-0.57	-1.63	0.012	0.096
KEGG_HUNTINGTONS_DISEASE	up	136	-0.34	-1.62	0.018	0.099
KEGG_BIOSYNTHESIS_OF_UNSATURATED_FATTY_ACIDS	up	19	-0.76	-1.62	0	0.092
KEGG_LYSINE_DEGRADATION	up	40	-0.46	-1.62	0.016	0.087
KEGG_TRYPTOPHAN_METABOLISM	up	35	-0.51	-1.61	0.008	0.088
KEGG_BETA_ALANINE_METABOLISM	up	21	-0.59	-1.54	0.006	0.145
KEGG_FRUCTOSE_AND_MANNOSE_METABOLISM	up	31	-0.45	-1.52	0.027	0.161
KEGG_ALPHA_LINOLENIC_ACID_METABOLISM	up	17	-0.62	-1.52	0.024	0.162
KEGG_ARACHIDONIC_ACID_METABOLISM	up	56	-0.52	-1.51	0.004	0.158
KEGG_ADIPOCYTOKINE_SIGNALING_PATHWAY	up	62	-0.36	-1.46	0.002	0.22
**T0901317**						
KEGG_COMPLEMENT_AND_COAGULATION_CASCADES	down	67	0.47	1.64	0.004	0.235
KEGG_GLYCINE_SERINE_AND_THREONINE_METABOLISM	down	30	0.56	1.63	0.002	0.182
KEGG_ALANINE_ASPARTATE_AND_GLUTAMATE_METABOLISM	down	27	0.48	1.62	0.015	0.149
KEGG_BASAL_TRANSCRIPTION_FACTORS	down	30	0.5	1.61	0.014	0.127
KEGG_ONE_CARBON_POOL_BY_FOLATE	down	15	0.61	1.6	0.015	0.119
KEGG_RIBOSOME	up	53	-0.67	-2.01	0	0.004
KEGG_B_CELL_RECEPTOR_SIGNALING_PATHWAY	up	66	-0.51	-1.83	0	0.068
KEGG_FC_GAMMA_R_MEDIATED_PHAGOCYTOSIS	up	83	-0.53	-1.8	0	0.055
KEGG_VIRAL_MYOCARDITIS	up	58	-0.56	-1.74	0.002	0.084
KEGG_RETINOL_METABOLISM	up	54	-0.6	-1.73	0	0.081
KEGG_METABOLISM_OF_XENOBIOTICS_BY_CYTOCHROME_P450	up	57	-0.6	-1.71	0	0.087
KEGG_FATTY_ACID_METABOLISM	up	38	-0.61	-1.71	0	0.08
KEGG_ARACHIDONIC_ACID_METABOLISM	up	56	-0.65	-1.7	0.002	0.073
KEGG_ALZHEIMERS_DISEASE	up	129	-0.36	-1.69	0.002	0.076
KEGG_APOPTOSIS	up	73	-0.41	-1.69	0.006	0.073
KEGG_DRUG_METABOLISM_CYTOCHROME_P450	up	57	-0.57	-1.67	0	0.081
KEGG_GLYCEROPHOSPHOLIPID_METABOLISM	up	67	-0.46	-1.66	0	0.079
KEGG_COLORECTAL_CANCER	up	56	-0.45	-1.65	0.014	0.08
KEGG_FC_EPSILON_RI_SIGNALING_PATHWAY	up	73	-0.45	-1.65	0.01	0.074
KEGG_LINOLEIC_ACID_METABOLISM	up	28	-0.65	-1.64	0.002	0.081
KEGG_LEISHMANIA_INFECTION	up	59	-0.65	-1.62	0.01	0.089
KEGG_LYSOSOME	up	111	-0.36	-1.6	0.019	0.11
KEGG_TYPE_I_DIABETES_MELLITUS	up	36	-0.59	-1.59	0.008	0.106
KEGG_PARKINSONS_DISEASE	up	92	-0.42	-1.59	0.046	0.103
KEGG_NOD_LIKE_RECEPTOR_SIGNALING_PAT	up	43	-0.48	-1.59	0.035	0.103

HWAY						
KEGG_OXIDATIVE_PHOSPHORYLATION	up	94	-0.36	-1.58	0.059	0.104
KEGG_PPAR_SIGNALING_PATHWAY	up	65	-0.58	-1.58	0.006	0.102
KEGG_RENAL_CELL_CARCINOMA	up	63	-0.37	-1.57	0.04	0.103
KEGG_LEUKOCYTE_TRANSENDOTHELIAL_MIGRATION	up	98	-0.4	-1.56	0.01	0.11
KEGG_ALLOGRAFT_REJECTION	up	31	-0.61	-1.55	0.027	0.11
KEGG_GRAFT_VERSUS_HOST_DISEASE	up	28	-0.67	-1.54	0.029	0.114
KEGG_CELL_ADHESION_MOLECULES_CAMS	up	112	-0.45	-1.54	0.01	0.117
KEGG_TOLL_LIKE_RECEPTOR_SIGNALING_PATHWAY	up	91	-0.47	-1.53	0.038	0.12
KEGG_ASTHMA	up	22	-0.7	-1.53	0.021	0.119
KEGG_VEGF_SIGNALING_PATHWAY	up	67	-0.35	-1.52	0.015	0.117
KEGG_BIOSYNTHESIS_OF_UNSATURATED_FATTY_ACIDS	up	19	-0.67	-1.5	0.031	0.133
KEGG_BLADDER_CANCER	up	36	-0.4	-1.46	0.043	0.177
KEGG_FRUCTOSE_AND_MANNOSE_METABOLISM	up	31	-0.36	-1.46	0.034	0.177
KEGG_GLYCEROLIPID_METABOLISM	up	42	-0.49	-1.45	0.049	0.183
KEGG_HUNTINGTONS_DISEASE	up	136	-0.3	-1.45	0.058	0.183
KEGG_PROPANOATE_METABOLISM	up	27	-0.5	-1.44	0.078	0.186
KEGG_ETHER_LIPID_METABOLISM	up	29	-0.48	-1.44	0.034	0.186
KEGG_SPHINGOLIPID_METABOLISM	up	31	-0.46	-1.44	0.074	0.182
KEGG_HEMATOPOIETIC_CELL_LINEAGE	up	72	-0.51	-1.44	0.035	0.178
KEGG_NON_SMALL_CELL_LUNG_CANCER	up	48	-0.35	-1.44	0.068	0.174
KEGG_PENTOSE_PHOSPHATE_PATHWAY	up	25	-0.49	-1.43	0.044	0.178
KEGG_MAPK_SIGNALING_PATHWAY	up	225	-0.34	-1.43	0.027	0.175
KEGG_P53_SIGNALING_PATHWAY	up	59	-0.42	-1.41	0.052	0.193
KEGG_THYROID_CANCER	up	23	-0.37	-1.41	0.057	0.191
KEGG_GLUTATHIONE_METABOLISM	up	45	-0.47	-1.41	0.076	0.189
KEGG_ANTIGEN_PROCESSING_AND_PRESENTATION	up	63	-0.45	-1.4	0.076	0.188
KEGG_ENDOCYTOSIS	up	144	-0.3	-1.4	0.026	0.185
KEGG_PATHOGENIC_ESCHERICHIA_COLI_INFECTION	up	42	-0.42	-1.39	0.113	0.193
KEGG_PROSTATE_CANCER	up	95	-0.33	-1.37	0.045	0.215
KEGG_CHRONIC_MYELOID_LEUKEMIA	up	63	-0.36	-1.36	0.135	0.232
KEGG_PANCREATIC_CANCER	up	65	-0.33	-1.35	0.102	0.233
KEGG_NATURAL_KILLER_CELL_MEDIATED_CYTOTOXICITY	up	99	-0.39	-1.35	0.117	0.23
KEGG_AUTOIMMUNE_THYROID_DISEASE	up	44	-0.48	-1.35	0.094	0.234
KEGG_PATHWAYS_IN_CANCER	up	305	-0.3	-1.34	0.059	0.231
KEGG_ECM_RECEPTOR_INTERACTION	up	72	-0.4	-1.34	0.101	0.232
KEGG_CHEMOKINE_SIGNALING_PATHWAY	up	149	-0.35	-1.33	0.079	0.243
KEGG_REGULATION_OF_ACTIN_CYTOSKELETON	up	181	-0.31	-1.33	0.056	0.244
KEGG_GLYCOLYSIS_GLUCONEOGENESIS	up	49	-0.39	-1.32	0.092	0.243
KEGG_SYSTEMIC_LUPUS_ERYTHEMATOSUS	up	98	-0.4	-1.32	0.163	0.243
**Atorvastatin**						
KEGG_SNARE_INTERACTIONS_IN_VESICULAR_TRANSPORT	down	32	0.48	1.81	0.008	0.109
KEGG_PROPANOATE_METABOLISM	up	27	-0.6	-1.85	0	0.08
KEGG_FATTY_ACID_METABOLISM	up	38	-0.5	-1.72	0.01	0.2

**Salicylate**						
No enriched gene sets						
**Rofecoxib**						
KEGG_MISMATCH_REPAIR	down	22	0.58	1.76	0.002	0.227
**LFD**						
KEGG_GLYCOLYSIS_GLUCONEOGENESIS	down	49	0.44	1.7	0.002	0.611
KEGG_RIBOSOME	up	53	-0.48	-1.99	0.004	0.016
KEGG_DRUG_METABOLISM_CYTOCHROME_P450	up	57	-0.57	-1.76	0.002	0.105
KEGG_RETINOL_METABOLISM	up	54	-0.55	-1.71	0.002	0.113
KEGG_METABOLISM_OF_XENOBIOTICS_BY_CYTOCHROME_P450	up	57	-0.55	-1.7	0.002	0.092
KEGG_TYROSINE_METABOLISM	up	33	-0.58	-1.65	0.01	0.117
KEGG_SPLICEOSOME	up	81	-0.32	-1.64	0.006	0.114
KEGG_N_GLYCAN_BIOSYNTHESIS	up	42	-0.39	-1.63	0.03	0.105
KEGG_TRYPTOPHAN_METABOLISM	up	35	-0.43	-1.55	0.035	0.182
KEGG_LINOLEIC_ACID_METABOLISM	up	28	-0.61	-1.55	0.012	0.162
KEGG_PROTEIN_EXPORT	up	20	-0.55	-1.51	0.074	0.198
KEGG_CYSTEINE_AND_METHIONINE_METABOLISM	up	28	-0.47	-1.47	0.07	0.229
KEGG_ASCORBATE_AND_ALDARATE_METABOLISM	up	19	-0.57	-1.46	0.076	0.225
KEGG_ALANINE_ASPARTATE_AND_GLUTAMATE_METABOLISM	up	27	-0.42	-1.44	0.047	0.245
KEGG_SELENOAMINO_ACID_METABOLISM	up	23	-0.45	-1.44	0.101	0.23
KEGG_FATTY_ACID_METABOLISM	up	38	-0.35	-1.44	0.118	0.215
KEGG_PHENYLALANINE_METABOLISM	up	16	-0.56	-1.43	0.057	0.216
KEGG_NITROGEN_METABOLISM	up	17	-0.59	-1.41	0.087	0.233
**HFD-9weeks**						
KEGG_RIBOSOME	up	53	-0.51	-2.05	0	0.001
**DLI**						
KEGG_RIBOSOME	up	53	-0.53	-2.05	0	0.004
KEGG_METABOLISM_OF_XENOBIOTICS_BY_CYTOCHROME_P450	up	57	-0.54	-1.73	0	0.11
KEGG_DRUG_METABOLISM_CYTOCHROME_P450	up	57	-0.55	-1.72	0	0.082
KEGG_RETINOL_METABOLISM	up	54	-0.48	-1.6	0.006	0.191
KEGG_PHENYLALANINE_METABOLISM	up	16	-0.62	-1.58	0.041	0.191
KEGG_NITROGEN_METABOLISM	up	17	-0.64	-1.51	0.049	0.246

4. Supplementary Table 4: GSEA analysis of the adipose transcriptome

This table presents the results of an analysis parallel to the one presented in Supplementary Table 3, conducted on the white adipose, rather than the liver, transcriptome. Refer to the legend of Supplementary Table 3 for further details.

Gene set	***Direction***	***Gene set size***	***ES***	***NES***	***Nominal p-value***	***FDR q-value***
**Rosiglitazone**						
KEGG_HUNTINGTONS_DISEASE	up	136	-0.55	-2.02	0	0.005
KEGG_PARKINSONS_DISEASE	up	92	-0.65	-1.95	0	0.005
KEGG_ALZHEIMERS_DISEASE	up	129	-0.44	-1.9	0.002	0.011
KEGG_AMINOACYL_TRNA_BIOSYNTHESIS	up	37	-0.55	-1.86	0.012	0.014
KEGG_PEROXISOME	up	72	-0.58	-1.79	0.012	0.026
KEGG_CITRATE_CYCLE_TCA_CYCLE	up	28	-0.83	-1.78	0.002	0.024
KEGG_OXIDATIVE_PHOSPHORYLATION	up	94	-0.58	-1.77	0.002	0.023
KEGG_PPAR_SIGNALING_PATHWAY	up	65	-0.62	-1.63	0.004	0.087
KEGG_FATTY_ACID_METABOLISM	up	38	-0.69	-1.62	0.024	0.09
KEGG_REGULATION_OF_AUTOPHAGY	up	30	-0.48	-1.6	0.006	0.088
KEGG_LYSINE_DEGRADATION	up	40	-0.51	-1.57	0.028	0.103
KEGG_CARDIAC_MUSCLE_CONTRACTION	up	54	-0.43	-1.54	0.011	0.124
KEGG_VALINE_LEUCINE_AND_ISOLEUCINE_DEGRADATION	up	41	-0.55	-1.5	0.085	0.151
KEGG_GLYCEROPHOSPHOLIPID_METABOLISM	up	67	-0.39	-1.47	0.019	0.175
KEGG_PYRUVATE_METABOLISM	up	33	-0.54	-1.47	0.09	0.17
KEGG_TASTE_TRANSDUCTION	up	41	-0.46	-1.42	0.033	0.222
**Pioglitazone**						
KEGG_TYPE_I_DIABETES_MELLITUS	down	36	0.55	1.44	0.053	0.246
KEGG_ANTIGEN_PROCESSING_AND_PRESENTATION	down	63	0.44	1.44	0.028	0.245
KEGG_PEROXISOME	up	72	-0.59	-1.86	0.002	0.044
KEGG_FATTY_ACID_METABOLISM	up	38	-0.69	-1.83	0	0.035
KEGG_PPAR_SIGNALING_PATHWAY	up	65	-0.62	-1.7	0	0.119
KEGG_AMINOACYL_TRNA_BIOSYNTHESIS	up	37	-0.47	-1.66	0.031	0.139
KEGG_CITRATE_CYCLE_TCA_CYCLE	up	28	-0.74	-1.63	0.008	0.148
KEGG_PARKINSONS_DISEASE	up	92	-0.44	-1.62	0.033	0.139
KEGG_BETA_ALANINE_METABOLISM	up	21	-0.53	-1.6	0.013	0.146
KEGG_REGULATION_OF_AUTOPHAGY	up	30	-0.46	-1.56	0	0.184
KEGG_PYRUVATE_METABOLISM	up	33	-0.53	-1.53	0.029	0.223
KEGG_LYSINE_DEGRADATION	up	40	-0.44	-1.49	0.046	0.263
KEGG_HUNTINGTONS_DISEASE	up	136	-0.35	-1.49	0.075	0.242
**T0901317**						
No enriched gene sets						
**Salicylate**						
KEGG_PARKINSONS_DISEASE	up	92	-0.46	-1.81	0.006	0.127
KEGG_AMINOACYL_TRNA_BIOSYNTHESIS	up	37	-0.49	-1.8	0.014	0.077
**LFD**						

KEGG_SYSTEMIC_LUPUS_ERYTHEMATOSUS	down	98	0.59	1.72	0	0.203
KEGG_ALLOGRAFT_REJECTION	down	31	0.62	1.7	0.004	0.18
KEGG_LEISHMANIA_INFECTION	down	59	0.65	1.69	0.016	0.141
KEGG_GRAFT_VERSUS_HOST_DISEASE	down	28	0.63	1.68	0.006	0.131
KEGG_TYPE_I_DIABETES_MELLITUS	down	36	0.56	1.67	0.006	0.121
KEGG_LYSOSOME	down	111	0.51	1.66	0.029	0.112
KEGG_PATHOGENIC_ESCHERICHIA_COLI_INFECTION	down	42	0.49	1.64	0.004	0.121
KEGG_CELL_CYCLE	down	108	0.48	1.64	0.008	0.109
KEGG_SPHINGOLIPID_METABOLISM	down	31	0.56	1.63	0.014	0.103
KEGG_TOLL_LIKE_RECEPTOR_SIGNALING_PATHWAY	down	91	0.5	1.62	0.006	0.101
KEGG_P53_SIGNALING_PATHWAY	down	59	0.48	1.6	0.018	0.125
KEGG_EPITHELIAL_CELL_SIGNALING_IN_HELICOBACTER_PYLORI_INFECTION	down	59	0.4	1.56	0.015	0.154
KEGG_NOD_LIKE_RECEPTOR_SIGNALING_PATHWAY	down	43	0.47	1.56	0.036	0.155
KEGG_HOMOLOGOUS_RECOMBINATION	down	23	0.47	1.54	0.052	0.161
KEGG_ECM_RECEPTOR_INTERACTION	down	72	0.43	1.54	0.006	0.154
KEGG_HEMATOPOIETIC_CELL_LINEAGE	down	72	0.5	1.54	0.028	0.147
KEGG_OTHER_GLYCAN_DEGRADATION	down	15	0.57	1.53	0.05	0.15
KEGG_B_CELL_RECEPTOR_SIGNALING_PATHWAY	down	66	0.5	1.52	0.04	0.157
KEGG_T_CELL_RECEPTOR_SIGNALING_PATHWAY	down	97	0.47	1.48	0.056	0.194
KEGG_GLIOMA	down	54	0.41	1.48	0.044	0.185
KEGG_PRIMARY_IMMUNODEFICIENCY	down	32	0.62	1.48	0.04	0.18
KEGG_NATURAL_KILLER_CELL_MEDIATED_CYTOTOXICITY	down	99	0.45	1.47	0.039	0.188
KEGG_CELL_ADHESION_MOLECULES_CAMS	down	112	0.43	1.47	0.014	0.19
KEGG_VIBRIO_CHOLERAE_INFECTION	down	46	0.41	1.45	0.053	0.205
KEGG_INTESTINAL_IMMUNE_NETWORK_FOR_IGA_PRODUCTION	down	37	0.55	1.45	0.075	0.2
KEGG_FC_EPSILON_RI_SIGNALING_PATHWAY	down	73	0.43	1.44	0.049	0.206
KEGG_COMPLEMENT_AND_COAGULATION_CASCADES	down	67	0.39	1.44	0.008	0.199
KEGG_FC_GAMMA_R_MEDIATED_PHAGOCYTOSIS	down	83	0.44	1.42	0.085	0.212
KEGG_JAK_STAT_SIGNALING_PATHWAY	down	140	0.33	1.41	0.006	0.225
KEGG_APOPTOSIS	down	73	0.33	1.41	0.087	0.219
KEGG_ANTIGEN_PROCESSING_AND_PRESENTATION	down	63	0.43	1.41	0.079	0.216
KEGG_VIRAL_MYOCARDITIS	down	58	0.42	1.4	0.097	0.215
KEGG_CHEMOKINE_SIGNALING_PATHWAY	down	149	0.39	1.39	0.034	0.229
KEGG_PROTEASOME	down	41	0.38	1.38	0.181	0.24
KEGG_CYTOKINE_CYTOKINE_RECEPTOR_INTERACTION	down	219	0.36	1.37	0.016	0.243
KEGG_CITRATE_CYCLE_TCA_CYCLE	up	28	-0.63	-1.7	0.01	0.149
KEGG_VALINE_LEUCINE_AND_ISOLEUCINE_DEGRADATION	up	41	-0.57	-1.69	0.004	0.105
KEGG_PROPANOATE_METABOLISM	up	27	-0.56	-1.67	0.012	0.107
KEGG_AMINOACYL_TRNA_BIOSYNTHESIS	up	37	-0.46	-1.66	0.025	0.096
KEGG_BETA_ALANINE_METABOLISM	up	21	-0.53	-1.65	0.01	0.084
KEGG_NICOTINATE_AND_NICOTINAMIDE_METABOLISM	up	18	-0.52	-1.52	0.027	0.24

**HFD-9weeks**						
KEGG_FRUCTOSE_AND_MANNOSE_METABOLISM	down	31	0.55	1.78	0.002	0.224
KEGG_AMINOACYL_TRNA_BIOSYNTHESIS	up	37	-0.48	-1.78	0.018	0.205
KEGG_VALINE_LEUCINE_AND_ISOLEUCINE_DEGRADATION	up	41	-0.55	-1.68	0.006	0.2
KEGG_SELENOAMINO_ACID_METABOLISM	up	23	-0.54	-1.67	0.016	0.164
KEGG_PROPANOATE_METABOLISM	up	27	-0.5	-1.65	0.02	0.153
KEGG_CITRATE_CYCLE_TCA_CYCLE	up	28	-0.65	-1.61	0.028	0.193
**DLI**						
KEGG_ASTHMA	down	22	0.59	1.59	0.029	0.234
KEGG_ECM_RECEPTOR_INTERACTION	down	72	0.48	1.57	0.018	0.242
KEGG_LEISHMANIA_INFECTION	down	59	0.51	1.52	0.061	0.244
KEGG_PROPANOATE_METABOLISM	up	27	-0.58	-1.89	0.002	0.041
KEGG_CITRATE_CYCLE_TCA_CYCLE	up	28	-0.62	-1.73	0	0.163
KEGG_BUTANOATE_METABOLISM	up	31	-0.62	-1.71	0.002	0.138
KEGG_VALINE_LEUCINE_AND_ISOLEUCINE_DEGRADATION	up	41	-0.53	-1.69	0.025	0.121
KEGG_LYSINE_DEGRADATION	up	40	-0.45	-1.61	0.014	0.223
KEGG_PYRUVATE_METABOLISM	up	33	-0.5	-1.58	0.077	0.241

5. Supplementary Table 5: Probes used to construct the liver gene expression space

N/A = Not available

	nuID	Entrez gene ID	Gene symbol	Synonym	Illumina probe ID
1	cfUjX9rkP0ie5SOchE	72056	1810055G02Rik	1810055G02Rik	ILMN_2650275
2	QuXFGy4Nc43EIdiVZI	70113	Odf3b	2010001J22Rik	ILMN_1251371
3	oCVCDSiEq64KK0eIK0	69134	Fam25c	2200001I15Rik	ILMN_2678637
4	WSu17KUufXnCEhU.e4	N/A	N/A	2310047D13Rik	ILMN_2543108
5	udDrktKbjeIeUee3NE	71003	Prss41	4931440B09Rik	ILMN_2650180
6	BoqeavtMncSIPPDn8Q	27413	Abcb11	Abcb11	ILMN_2758509
7	3b4uKAJAL6LZJ2eg9U	26874	Abcd2	Abcd2	ILMN_1243944
8	utxBTSj1N5hX7t4V_4	27409	Abcg5	Abcg5	ILMN_2725781
9	ZkRr6dUvnm574d4_Xg	67470	Abcg8	Abcg8	ILMN_2789904
10	9gitW3XBeNXYaUJezs	216725	Adamts2	Adamts2	ILMN_2729103
11	KinVdDt3JFDlHgjRUg	216725	Adamts2	Adamts2	ILMN_1226259
12	fegNclOadFldOebves	268822	Adck5	Adck5	ILMN_2918317
13	96FK4Vku279xIpnNQk	71562	Afmid	Afmid	ILMN_2702894
14	c5zrnXpWehQk6bRFQU	71562	Afmid	Afmid	ILMN_2626209
15	9uo3GF6JV0yUXVIOFc	71562	Afmid	Afmid	ILMN_2742295
16	uuhSuFZLtu.cSKZzUI	71562	Afmid	Afmid	ILMN_2702893
17	TrSTh1Ui9RaS1Ci9xg	71562	Afmid	Afmid	ILMN_2597013
18	BncUheMHPXkkRR6l94	71562	Afmid	Afmid	ILMN_2827607
19	inm67o0f4TTIg3hWoo	71562	Afmid	Afmid	ILMN_2600443
20	WO_6gegqBExEiFfm4o	71760	Agxt2l1	Agxt2l1	ILMN_2661820
21	NlO_6gegqBExEiFfm4	71760	Agxt2l1	Agxt2l1	ILMN_1229990
22	Z3ELkBrfFR4lN30aNk	269378	Ahcy	Ahcy	ILMN_2852533
23	3l4QV19JSJ7tRS1oEw	432720	Akr1c19	Akr1c19	ILMN_3160292
24	ros_UG67goKpRHre64	107747	Aldh1l1	Aldh1l1	ILMN_3100276
25	xpf4g6HuqAjA5m4WqU	107747	Aldh1l1	Aldh1l1	ILMN_3027287
26	EpEpFh6KeOqQIEu_DE	74018	Als2	Als2	ILMN_2620053
27	0qbJt7hOIR9d87Hu24	12306	Anxa2	Anxa2	ILMN_2657175
28	0XogiXiogrQdfziRXo	11808	Apoa4	Apoa4	ILMN_2834123
29	EV8V_tU71I9dOXn4d0	66113	Apoa5	Apoa5	ILMN_2641301
30	uqpXUicwJeUFR6LHos	11813	Apoc2	Apoc2	ILMN_2647820
31	HdTAJRdIlPjELQ3FO4	11865	Arntl	Arntl	ILMN_2707510
32	uJCcBQLiv9UpfoV93U	74008	Arsg	Arsg	ILMN_2732601
33	KuwCwO.fWneCtiEmEQ	27053	Asns	Asns	ILMN_2643513
34	f0r444Djt5JdUgv1fQ	27053	Asns	Asns	ILMN_3006123

35	HRAuB61uPgBUu_e0N4	54140	Avpr1a	Avpr1a	ILMN_1242999
36	ln0qzHfq..UrM87OF8	230789	Fam76a	BC008163	ILMN_1242176
37	iUol5eQoCqI3KFVw0g	227622	BC029214	BC029214	ILMN_2768209
38	H5u.9cAxTJHtJh_rnU	208164	Fam180a	BC064033	ILMN_2833163
39	9UngGQivgB0jTVEjWE	63857	Bcmo1	Bcmo1	ILMN_2713638
40	fmSurpXAXSpQzrn3Nw	71911	Bdh1	Bdh1	ILMN_1231553
41	N6tXjusorjrS_RUkIo	12111	Bgn	Bgn	ILMN_2964042
42	TcqIpivEu.M.6BfXgo	20893	Bhlhe40	Bhlhb2	ILMN_1249378
43	QGSK6CkmF5VQpwqwbk	55950	Bri3	Bri3	ILMN_2700334
44	HERI41YuB1BXREfJSk	110382	C8b	C8b	ILMN_1227404
45	TrfRI55fopUuYfgI3g	67426	Adck3	Cabc1	ILMN_2800813
46	90TfNULVF1BDP0ufeQ	12338	Capn6	Capn6	ILMN_2695143
47	rq3K7qDfp6O3_X3np4	23831	Car14	Car14	ILMN_2973824
48	Zb.t7_jc75937GgSsM	231214	Cc2d2a	Cc2d2a	ILMN_2704619
49	HnElNcHl0g0dC99R2s	20308	Ccl9	Ccl9	ILMN_2776603
50	We9.KtMEv1kt6TwKT4	270166	Clpx	Clpx	ILMN_2613869
51	xnLoik_v1SekU8sEik	270166	Clpx	Clpx	ILMN_3154849
52	cX26Ex0Tp_6O.dw9P0	68396	Nat8	Cml4	ILMN_1216539
53	0UCfEod46vuDv7nPXs	107581	Col16a1	Col16a1	ILMN_1248099
54	EJWL20G7n3r_5dQl9o	246277	Csad	Csad	ILMN_2810624
55	il5kQPtRAbohNP_vsg	107869	Cth	Cth	ILMN_2733193
56	x5iOh9Lz.kV_wBkp9Q	55985	Cxcl13	Cxcl13	ILMN_2760019
57	fuYQk6CjkSvivJFyKU	13074	Cyp17a1	Cyp17a1	ILMN_2874352
58	lVSeUzcil9NeiNxYxE	13077	Cyp1a2	Cyp1a2	ILMN_2739847
59	ZuheEVBzq_FOCVqF7g	13077	Cyp1a2	Cyp1a2	ILMN_2795106
60	QblOqdWmeelLmd5F9s	13082	Cyp26a1	Cyp26a1	ILMN_2691295
61	xsnnRFfX3HvvdQJ1Ck	13094	Cyp2b9	Cyp2b9	ILMN_2617625
62	HS.3j1_787s1PfiwAw	13095	Cyp2c29	Cyp2c29	ILMN_2769991
63	9e40V4p.D0ef1EehEc	13096	Cyp2c37	Cyp2c37	ILMN_2691060
64	Z3uNFeKfw9Hn9RHoRE	13096	Cyp2c37	Cyp2c37	ILMN_2691059
65	3dTNe0HPKhN3dHHXdE	72082	Cyp2c55	Cyp2c55	ILMN_2736539
66	QXtc7F5N6y1.ODpKjk	226105	Cyp2c70	Cyp2c70	ILMN_1245514
67	6ko4jY4p3lQQpF1Rs4	13112	Cyp3a11	Cyp3a11	ILMN_2753183
68	ZdV9Ad1F.SfE.CLflM	13118	Cyp4a12b	Cyp4a12b	ILMN_1217072
69	l7bUp7YIwlSqfG7N6I	28042	Ept1	D5Wsu178e	ILMN_1253323
70	xUut6UnK5WCBtOOSEA	13170	Dbp	Dbp	ILMN_2616226
71	9XVJXSyiRs8MLIlE6g	13171	Dbt	Dbt	ILMN_2820948
72	Wgp9kCnOZXw6ISt0kk	13190	Dct	Dct	ILMN_1251894
73	BWhQei4VUQJwpTno2w	67880	Dcxr	Dcxr	ILMN_2868280
74	BTtt1iEL_5xn_e7mdk	13195	Ddc	Ddc	ILMN_2628647

75	KX0hOexHHq4eoBSA5w	54722	Dfna5	Dfna5h	ILMN_2652482
76	NVQpex1EUfocTEXAek	23856	Dido1	Dido1	ILMN_1257214
77	iv7XgrWekiHoISp4v4	13370	Dio1	Dio1	ILMN_2772070
78	3deCOG0AnQ1O7UP_Uc	13370	Dio1	Dio1	ILMN_2647234
79	EdkK7qf_wfRK7qiIeU	13436	Dnmt3b	Dnmt3b	ILMN_1252310
80	rI8pXoRCYf4VIufVSI	207521	Dtx4	Dtx4	ILMN_2651706
81	xuYs4mVP66OQSviKCo	67603	Dusp6	Dusp6	ILMN_2925711
82	rk0UtoggRfMVDkjFBQ	503845	Ear12	Ear12	ILMN_2814127
83	3L1GolECSEqATq1gnk	13587	Ear2	Ear2	ILMN_1232396
84	uWCefi1Yf7deee_ofs	53877	Ear4	Ear4	ILMN_2868480
85	QqPtGFPOOghOAU6Xgk	13909	Ces3b	EG13909	ILMN_2733745
86	ohKcUISD0gDUKJXaX0	241041	Gm4956	EG241041	ILMN_2911009
87	6pwHkgiNnm6CRq4Uhc	12686	Elovl3	Elovl3	ILMN_2682207
88	otcPJfur.e.oKL5pN4	68801	Elovl5	Elovl5	ILMN_2652757
89	xukfSH.vdevjQrvl64	170439	Elovl6	Elovl6	ILMN_2614752
90	oXJxF7qyd.xRWJXFVA	98845	Eps8l2	Eps8l2	ILMN_1223406
91	lQH0HXo..Ls3hEgRas	14026	Evl	Evl	ILMN_2731454
92	xnuSc_tE_q7EUq3VLE	14068	F7	F7	ILMN_2708871
93	BJcO0s3u7lDnvFE164	76267	Fads1	Fads1	ILMN_2607786
94	iVR64Ld3mUJOk_DFKI	56473	Fads2	Fads2	ILMN_2713071
95	HUrxR5FU9bqe9M3t0k	98952	Fam102a	Fam102a	ILMN_1225348
96	ceJOsiX8SpYJVJzm14	56636	Fgf21	Fgf21	ILMN_2710698
97	QUuV5FCPjzfSVNUHPw	14251	Flot1	Flot1	ILMN_1241618
98	fT0b5DgKp.VXdbcIEc	114142	Foxp2	Foxp2	ILMN_3140000
99	ZU5XfH1n10R06X1O30	14287	Fpgs	Fpgs	ILMN_2747070
100	WvsXu1lPoJXoor6HlM	14287	Fpgs	Fpgs	ILMN_1233334
101	uqJl0d5HvJKOkn0EdE	103988	Gck	Gck	ILMN_2698004
102	ishclTUNDudqAu9.PQ	14609	Gja1	Gja1	ILMN_1244291
103	ciiXb5RL_UsEeU0h6I	229599	Gm129	Gm129	ILMN_3102736
104	fVKd59.VePVD_zA7O8	229599	Gm129	Gm129	ILMN_3029489
105	orHq1.d7R2u0ujDu70	14733	Gpc1	Gpc1	ILMN_2635784
106	oflNeOAHliiB_hZIvU	14571	Gpd2	Gpd2	ILMN_1247257
107	HfWh5yqiCOpi7E.MOk	14756	Gpld1	Gpld1	ILMN_2673522
108	cupqXVeJW3uuUlfLk8	14778	Gpx3	Gpx3	ILMN_2715546
109	ZkvdR7gK7QXoUxoY48	14773	Grk5	Grk5	ILMN_1249435
110	9wgvXkFaC0igl11I6U	14860	Gsta4	Gsta4	ILMN_2892441
111	BtfjSTYO.gVG5ehl9U	14867	Gstm6	Gstm6	ILMN_1246321
112	cCpdUjSeAdx2i9eqCQ	14867	Gstm6	Gstm6	ILMN_2633096
113	cqj3X3OvjzfH6X6.SY	14873	Gsto1	Gsto1	ILMN_1254523
114	Hrr33gIDQgnOol9Si8	56794	Hacl1	Hacl1	ILMN_2739364

115	NJHru_OiIi3SgeXsO4	15109	Hal	Hal	ILMN_2984332
116	ZbUxEgnTTVhfgIitQU	15486	Hsd17b2	Hsd17b2	ILMN_1213811
117	cLqxnuXuuGuvImOeBA	53415	Htatip2	Htatip2	ILMN_2603834
118	9caWIY4guJRESdgpwA	114663	Impa2	Impa2	ILMN_2662160
119	00sAKrngdsL3yn1V2I	16326	Inhbe	Inhbe	ILMN_1229605
120	WnVPR3Unt99eX0.gh0	54139	Irf6	Irf6	ILMN_1216279
121	Z6j0lP1p0igjIonomI	16768	Lag3	Lag3	ILMN_2719811
122	BvVHsXu0_fuzh_ggqU	16792	Laptm5	Laptm5	ILMN_1217849
123	Zi6gH_T93dfNffsITU	16854	Lgals3	Lgals3	ILMN_1223317
124	l3tR_9ECSX0Ru0uIvY	N/A	N/A	LOC100043671	ILMN_1258600
125	WzoiT1HVo26SeF5Dos	N/A	N/A	LOC100045567	ILMN_1256633
126	0LT9Hsu5EGm3t6u1_4	N/A	N/A	LOC100046232	ILMN_2595732
127	9QVQKdenGBzEIZDs48	N/A	N/A	LOC100047046	ILMN_2635387
128	WXonq6AtFXzTf54e4U	N/A	N/A	LOC100047937	ILMN_2706906
129	EDaQVXoSIxQU6VkgU4	N/A	N/A	LOC100047937	ILMN_2703392
130	fYmCiJ3SoSMcB3SIeo	N/A	N/A	LOC100048733	ILMN_2759344
131	TXXrfnp9CPIC6TP43o	N/A	N/A	LOC676420	ILMN_2749958
132	9j5E4o8a6R0Tg3qRCk	67580	Lrrc18	Lrrc18	ILMN_2728504
133	ZqeTe7.ldhTfO3wuic	67867	Lrrc28	Lrrc28	ILMN_2700699
134	6bDkE3kBENO5VRQFk0	109245	Lrrc39	Lrrc39	ILMN_2746123
135	H6d6uvhK_nuoXeen5w	17304	Mfge8	Mfge8	ILMN_3133448
136	QKnnHuDOUKHh6lydoE	76574	Mfsd2a	Mfsd2	ILMN_1225764
137	lVLR1UbMdcuqBezafI	23945	Mgll	Mgll	ILMN_2700408
138	ufUJWF6KOrSFXwe4OI	17347	Mknk2	Mknk2	ILMN_2733887
139	l6J5Foh46d5mJkO55I	64144	Mllt1	Mllt1	ILMN_2756121
140	HhKuXzH_9y5PUK79Kk	75104	Mmd2	Mmd2	ILMN_1252636
141	BJei8NMDqXqNMkUzns	338467	Morc3	Morc3	ILMN_2874739
142	QOSkuuznhIrZ56t9Gc	381269	Mreg	Mreg	ILMN_2976159
143	0SncVLXohCglUSiu4A	17925	Myo9b	Myo9b	ILMN_2612484
144	ESdC5ZVdFlD3Qevluw	83814	Nedd4l	Nedd4l	ILMN_2878501
145	BXSKiToPsMuEXGNZOg	56349	Net1	Net1	ILMN_2610771
146	lq_HSp1ed1QKM5nkgA	106582	Nrm	Nrm	ILMN_2733524
147	HeEU03ekX1FDoCKkUY	18391	Sigmar1	Oprs1	ILMN_1238081
148	0JCkYov4fTdKB2Tigk	23972	Papss2	Papss2	ILMN_2638349
149	9SaUKcQQejfTtR0ceo	18551	Pcsk4	Pcsk4	ILMN_1245529
150	BVLoIju18dP_iSsS6k	18551	Pcsk4	Pcsk4	ILMN_1252464
151	WK01VH0eUSWOX0CKFk	72599	Pdia5	Pdia5	ILMN_1255177
152	0WOCH_FL_4KJHXe120	72599	Pdia5	Pdia5	ILMN_2607066
153	okuH9Ekof4dUT6_NEQ	27273	Pdk4	Pdk4	ILMN_1259322
154	xll76CD_TsSoLZo6IU	55983	Pdzrn3	Pdzrn3	ILMN_3156010

155	Kqat9rwr54Qnngut7U	18626	Per1	Per1	ILMN_2813484
156	9ndIv7mOOPbiX5aEIo	18627	Per2	Per2	ILMN_2987862
157	flCTUle.38g7ufEau0	18627	Per2	Per2	ILMN_2987863
158	of33UCE7qJfQ10uk6g	74769	Pik3cb	Pik3cb	ILMN_2680549
159	uJ5QgSar9ZpddOGZJQ	56695	Pnkd	Pnkd	ILMN_1221275
160	97qi0pC5IldX0Ra90Q	19157	Cyth1	Pscd1	ILMN_2868827
161	BFSnVP54LALHi.1HxQ	59038	Pxmp4	Pxmp4	ILMN_2688639
162	BtFOPzWQJSpYHXLlF4	19369	Raet1b	Raet1b	ILMN_3158725
163	Tl11cUh3F3kJfqDR4o	19734	Rgs16	Rgs16	ILMN_2600744
164	NVamOKn.0.XXSesHSo	20111	Rps6ka1	Rps6ka1	ILMN_2975718
165	6uKWXsPk7RItR7vn6U	235636	Rtp3	Rtp3	ILMN_2864416
166	fn6VEsk0nPUzXQ.k9U	N/A	N/A	Samd9l	ILMN_1224855
167	rXFU7v7Ax3p9f6V4oo	104175	Sbk1	Sbk	ILMN_2724545
168	l3f0.rFexVLl3m2XX0	20276	Scnn1a	Scnn1a	ILMN_2729607
169	f5L3jN6N4KdEcqlVdQ	20342	Selenbp2	Selenbp2	ILMN_2939652
170	0ehh1NdAfzoeyurl5I	66222	Serpinb1a	Serpinb1a	ILMN_1231573
171	627v5cTXs7ud1rgigg	20500	Slc13a2	Slc13a2	ILMN_2646369
172	Eu4U7Te4Iu_7T9OT6U	216227	Slc17a8	Slc17a8	ILMN_2806439
173	EvXnQo_oVCt1EkxQNU	212980	Slc45a3	Slc45a3	ILMN_1218226
174	xrtPe03uQc8.X_ov10	330064	Slc5a6	Slc5a6	ILMN_1225056
175	WVlnlS7uSByr.AtbK8	14664	Slc6a9	Slc6a9	ILMN_2667384
176	Wl9KiX_8eku9.eCUWo	24059	Slco2a1	Slco2a1	ILMN_2961216
177	ZcLKvi6p5fft1KYNPE	319317	Snhg11	Snhg11	ILMN_2952098
178	Kcs_FcINeIlTM7.U4k	66442	Spc25	Spc25	ILMN_1255960
179	lNNUBTl3_KwT_4ngBI	66442	Spc25	Spc25	ILMN_2901180
180	iop7gpDpDBxSJCii7c	20733	Spint2	Spint2	ILMN_2645845
181	NJWfB17XDk33RMieHo	114715	Spred1	Spred1	ILMN_2490252
182	lXOAeKE5Xq64vshYKU	68792	Srpx2	Srpx2	ILMN_2698728
183	BXbuURJDtiBEBcpu3U	20442	St3gal1	St3gal1	ILMN_2749178
184	HJWB6gXqmOkKuCOJ7c	71116	Stx18	Stx18	ILMN_2612350
185	9QNvcaVUDc16FLU.Vc	69083	Sult1c2	Sult1c2	ILMN_2612973
186	W33pV4V7n6Iinnaulg	211389	Suox	Suox	ILMN_2912532
187	KXJ559ff_v_7xKL96I	74126	Syvn1	Syvn1	ILMN_2628258
188	loCX6Tr3sCd_xZ6IBo	21810	Tgfbi	Tgfbi	ILMN_2834379
189	EzinRbomCFiT1nu_X8	21826	Thbs2	Thbs2	ILMN_2635229
190	ZkQoBQhG4l75SgR9R0	380712	Tlcd2	Tlcd2	ILMN_2814385
191	iXuHlOb1FICWdz.rd4	223693	Tmem184b	Tmem184b	ILMN_2697433
192	69IUiowc.tHCkBdeJU	66279	Tmem218	Tmem218	ILMN_2639402
193	WUkRQQ5.fUneouL3A4	67893	Tmem86a	Tmem86a	ILMN_2645662
194	KX9e6SCUoQjoohCh.4	29820	Tnfrsf19	Tnfrsf19	ILMN_2793522

195	rVddRQh9795x.59IpY	83433	Trem2	Trem2	ILMN_2992709
196	00e5PHiFe0Ql97VURI	218793	Ube2e2	Ube2e2	ILMN_2792485
197	ou9N69yF6jF5bnsRP0	216558	Ugp2	Ugp2	ILMN_1244631
198	iUcxJceWC1rxKYOkR8	53376	Usp2	Usp2	ILMN_1240264
199	iX93et.k.fXrWOAk3U	67701	Wfdc2	Wfdc2	ILMN_1236758
200	9MHuVJwX7COD6ok1Xc	22764	Zfx	Zfx	ILMN_3024592

6. <u>Supplementary Table 6: Probes used to construct the adipose gene expression space</u>

N/A = Not available

	nuID	Entrez gene ID	Gene symbol	Synonym	Illumina probe ID
1	ZteIg.lKU0fNffMaBc	Fam198b	1110032E23Rik	ILMN_1235811	68659
2	l6Fws33vr8U_Ld9XiI	Hilpda	2310016C08Rik	ILMN_2926198	69573
3	E7SbiV6hXaPugD13lc	Snrnp25	3300001G02Rik	ILMN_2990229	78372
4	6hVAOyYB_rNKU.TXSE	Txlng	4932441K18Rik	ILMN_2895908	353170
5	KjUJK_qBfrHsd39y.I	Unc79	9030205A07Rik	ILMN_1222844	217843
6	6lW51dcMvXqd.c_4e0	Aacs	Aacs	ILMN_1253601	78894
7	ftkShJB0XeBLxUUCCk	Abi3bp	Abi3bp	ILMN_3132588	320712
8	319TZItJ77SS1IVTSI	Acsm3	Acsm3	ILMN_3111685	20216
9	6Uq7r41xh1W519SQHc	Adap2	Adap2	ILMN_2589256	216991
10	Q0X1SUle_CUpItRZ0E	Adrb3	Adrb3	ILMN_2764057	11556
11	0YhXaS5RQRfQYBLeCc	Adssl1	Adssl1	ILMN_1245079	11565
12	E5UV.6uaKQN4EE_uEU	Agt	Agt	ILMN_1227398	11606
13	xeK0Eg2Ufq1Ig5R0Q0	Angpt1	Angpt1	ILMN_1226520	11600
14	BpP96ngxIpq1x1_Kks	Angptl1	Angptl1	ILMN_2874422	72713
15	QA57CW61NUvly5_q9Q	Aox3	Aox3	ILMN_2640097	71724
16	Wkcfd5Ll_qgPXceOxI	Ar	Ar	ILMN_2684075	11835
17	ZdS56UHip5_iKeB76A	Arhgap25	Arhgap25	ILMN_3155245	232201
18	Ku0qNAd6l17O4JLTQ4	Arl4a	Arl4a	ILMN_3144984	11861
19	KljCLigMV8.une8Q64	Asns	Asns	ILMN_2636755	27053
20	f0r444Djt5JdUgv1fQ	Asns	Asns	ILMN_3006123	27053
21	EuOr7vudbng57dlN3U	Atp1b1	Atp1b1	ILMN_2767615	11931
22	964X7_BV3tf_5WvG40	Atp6v0a1	Atp6v0a1	ILMN_1247682	11975
23	WneUp8xSIJQjOvfUcE	Ces1f	AU018778	ILMN_1238140	234564
24	oRbp9UJf7ijwEfesLk	Fam83f	AW544981	ILMN_2759499	213956
25	94kSJdRoisuFKeVOKA	Fam20c	BC004044	ILMN_1240719	80752
26	TiHKrXuX3I3_TXQJUk	Bcat1	Bcat1	ILMN_3131478	12035
27	NsXQgcWTZRfQgelfXo	Birc5	Birc5	ILMN_2681241	11799
28	xyNBQlAS9dAkTtl1Kg	Blnk	Blnk	ILMN_2726931	17060
29	3oJ9Ixd4BqlKv63zQQ	Blvra	Blvra	ILMN_1257284	109778
30	QsudAtxXBOEth17n7I	Bmp3	Bmp3	ILMN_1235433	110075
31	EUjpcTv4HlMlLu59ZI	Bnc2	Bnc2	ILMN_2721466	242509
32	cUo_5ahd7lF7gkLd3c	C2	C2	ILMN_2612895	12263
33	ZSjteFN1U.SIuzSPqg	C6	C6	ILMN_1216720	12274
34	xoo0Kd6IwjkC5eefpk	C6	C6	ILMN_2798129	12274
35	ZYg.Rv7o9x_1JF3pGk	Car13	Car13	ILMN_1249727	71934

36	oUkI41Q4sp6InEIo0U	Ccl2	Ccl2	ILMN_1245710	20296
37	3GtcKjKJ7eyg7gEtGU	Ccl7	Ccl7	ILMN_2835117	20306
38	WlMh666tRoN5XuE4Ck	Ccnd1	Ccnd1	ILMN_2601471	12443
39	0pTIeuurUaDeV7hOAo	Ccnd1	Ccnd1	ILMN_1221503	12443
40	9VRK1X6vhKBH_oi7rw	Cd6	Cd6	ILMN_2769330	12511
41	6lud.efPdPWO_k.e9Y	Cd84	Cd84	ILMN_2754698	12523
42	TllK.BB1S9OrVWulJQ	Cited2	Cited2	ILMN_2905866	17684
43	NQhFSDqcRMQaFRt5Ts	Cldn10	Cldn10	ILMN_1214954	58187
44	6vuQiB7XRDLX1Cndew	Cldn10	Cldn10	ILMN_2515816	58187
45	EN5vFj1FrtlB5Ci9V0	Cldn10	Cldn10	ILMN_2723576	58187
46	E1eId60nnuP3N8ddJE	Clec4b1	Clec4b1	ILMN_2603898	69810
47	6UTR3kXeyi0X7wnfuE	Clic6	Clic6	ILMN_2667635	209195
48	iAFACiqBFJ0eSS91fc	Col12a1	Col12a1	ILMN_2862538	12816
49	0UCfEod46vuDv7nPXs	Col16a1	Col16a1	ILMN_1248099	107581
50	3fTp1VE9VsN41CUnN0	Col6a1	Col6a1	ILMN_1259388	12833
51	Et618unLX1HeAkCuc0	Col6a1	Col6a1	ILMN_2768087	12833
52	ukR7EfUaUSfUMQkSql	Cp	Cp	ILMN_3083163	12870
53	3Uumgk2HqfOF_k0DHQ	Cpa2	Cpa2	ILMN_1216566	232680
54	NXOPGgl8_j666YaAqA	Crtac1	Crtac1	ILMN_2764112	72832
55	QXtc7F5N6y1.ODpKjk	Cyp2c70	Cyp2c70	ILMN_1245514	226105
56	cd3RON9XwV6l5Rs0sc	Cyp2f2	Cyp2f2	ILMN_2702903	13107
57	cRdJ2C9fvJ7n7Psd0U	Cytip	Cytip	ILMN_1251748	227929
58	TurRSxLdz6F.9IQmKI	Dbf4	Dbf4	ILMN_2952661	27214
59	ohnL5SeXnXDsRS0ISA	Ddah1	Ddah1	ILMN_1256676	69219
60	KX0hOexHHq4eoBSA5w	Dfna5	Dfna5h	ILMN_2652482	54722
61	HJOed5EoBR11E5K49g	Dido1	Dido1	ILMN_3016099	23856
62	Epfl51zUdB1RJc4I6o	Dnajb13	Dnajb13	ILMN_2667257	69387
63	cSl9q.d17_PlKOXe5I	Dnmt3l	Dnmt3l	ILMN_3112268	54427
64	6orlxp3kQvVrqMF5Ts	Dnmt3l	Dnmt3l	ILMN_1250149	54427
65	Tu3ExUudMii_eIYp6A	Dpep2	Dpep2	ILMN_2692315	319446
66	ftR.NR15ZdBqrB5fuQ	Dusp15	Dusp15	ILMN_2675090	252864
67	BUEiVRLX13_rHY9KTo	Ebf2	Ebf2	ILMN_1251248	13592
68	r7azVIFkisQVI7VSUI	Vsig8	EG240916	ILMN_2824741	240916
69	Kul1UI6d8n_Q4heCwk	Cela1	Ela1	ILMN_2693403	109901
70	o6fySSV69fVLTHzoI4	Ephx1	Ephx1	ILMN_2664224	13849
71	KlW0.Cfl8Db_B.tB0M	Fabp5	Fabp5	ILMN_1235908	16592
72	BJcO0s3u7lDnvFE164	Fads1	Fads1	ILMN_2607786	76267
73	TU1.kUCsDgcI.PRh1U	Fam13a	Fam13a	ILMN_1224427	58909
74	rod6umOmNf1ufJT51w	Fcgr4	Fcgr4	ILMN_2631161	246256
75	3U4lEGDQbJS4qQBip0	Fgf13	Fgf13	ILMN_1257196	14168

76	NdF7E7ELXXtPo.pG4E	Fgf13	Fgf13	ILMN_2745480	14168
77	ceJOsiX8SpYJVJzm14	Fgf21	Fgf21	ILMN_2710698	56636
78	WNoI1QKdSr2EJl557E	Fn3k	Fn3k	ILMN_1223313	63828
79	HKKkXSkIoh4h.IlCl4	Gadd45b	Gadd45b	ILMN_2900653	17873
80	uJKv6ERJHp13ZOteQQ	Gas6	Gas6	ILMN_2686327	14456
81	Ts3sEd_GXsUbhQVEr4	Gata6	Gata6	ILMN_2868133	14465
82	KofgjVf8Qa6d6Xf7eg	Accsl	Gm1967	ILMN_2787817	381411
83	ucflQByWjQfReTQ5Sg	Got1l1	Got1l1	ILMN_1246289	76615
84	orHq1.d7R2u0ujDu70	Gpc1	Gpc1	ILMN_2635784	14733
85	lltVHgjXTS7pTc7puc	Gpc3	Gpc3	ILMN_2719973	14734
86	oflNeOAHliiB_hZIvU	Gpd2	Gpd2	ILMN_1247257	14571
87	uh7f2CMaWpMT1Tdgrg	Gpnmb	Gpnmb	ILMN_2614655	93695
88	QXideEA8w6lan_7OuY	Gpnmb	Gpnmb	ILMN_2648669	93695
89	KuEKqJJx6gi4PNe4z4	Gpr64	Gpr64	ILMN_3113571	237175
90	cupqXVeJW3uuUlfLk8	Gpx3	Gpx3	ILMN_2715546	14778
91	cqWPcQpTv0ifUJNJxY	Grhl1	Grhl1	ILMN_1246419	195733
92	um3u0PSshOHhigaWjk	Gsta3	Gsta3	ILMN_1241437	14859
93	0IkPj9Xqnk4OOjEkqE	Gsta3	Gsta3	ILMN_3138685	14859
94	9wgvXkFaC0igl11I6U	Gsta4	Gsta4	ILMN_2892441	14860
95	cV41eX9NKeARJ3cgP8	Gtse1	Gtse1	ILMN_2908070	29870
96	cfShFUf37tMeXc9K1U	H2-DMb1	H2-DMb1	ILMN_1244977	14999
97	rCVSML.d5dfvqCi7pU	H2-M2	H2-M2	ILMN_2964185	14990
98	0eTXFa3vip3hTp.NBQ	Hn1l	Hn1l	ILMN_2851251	52009
99	05zVBWp1ieVeCmftEo	Igfbp2	Igfbp2	ILMN_1236788	16008
100	NeC5B5t1dJx2inx51Q	Inmt	Inmt	ILMN_2803249	21743
101	o.lEJt2o_7_HXt7UIE	Isl1	Isl1	ILMN_2727472	16392
102	NAEqrqCbpZd_R5.t1o	Itpk1	Itpk1	ILMN_2723920	217837
103	KweVopFpXqXykXQof8	Kcnh2	Kcnh2	ILMN_1244402	16511
104	WoY0SJwYOgCoKCKeKU	Kcnj14	Kcnj14	ILMN_2898924	211480
105	WfpZSUkSa1d_EcX3.A	Kif22	Kif22	ILMN_2762326	110033
106	3IwkIWC0aF9hoV1Kl8	Krt19	Krt19	ILMN_2614462	16669
107	u7ZSXeV_g02UGToIiQ	Lama1	Lama1	ILMN_2973288	16772
108	r15qhfuba4yoDAHlV0	Lat2	Lat2	ILMN_3143483	56743
109	WJdnNUKfe5LPEKI00Q	Lctl	Lctl	ILMN_1235276	235435
110	Zi6gH_T93dfNffsITU	Lgals3	Lgals3	ILMN_1223317	16854
111	0XTaMUs6kQAgpw597g	Lilrb4	Lilrb4	ILMN_1236702	14728
112	Eoj31cQ0XofTepjo5k	Lipf	Lipf	ILMN_2863532	67717
113	l3tR_9ECSX0Ru0uIvY	N/A	LOC100043671	ILMN_1258600	N/A
114	xPKK7Os8nk0e7o7Q3w	N/A	LOC100048295	ILMN_2470646	N/A
115	9n1EZxdJ6n3PwKISDU	Lrig3	Lrig3	ILMN_1213273	320398

116	KojfYEAZ6rlZVYNUv8	Ltc4s	Ltc4s	ILMN_2658687	17001
117	TV_c3RBcXlU7ESr.0A	Maob	Maob	ILMN_2719069	109731
118	olKFXjV4JICQihMjko	Matk	Matk	ILMN_2743902	17179
119	llOodoyA5cJ3XRfy7o	Mcm10	Mcm10	ILMN_2970532	70024
120	NSQJPrTu7P.Uy1Rfok	Meis1	Meis1	ILMN_1218266	17268
121	QgLUJ4MsDBJcqknXUo	Mest	Mest	ILMN_2642417	17294
122	QAtQngywMElyqSddSo	Mest	Mest	ILMN_2642418	17294
123	Wkg.XAHlKTwgeTe5SI	Mest	Mest	ILMN_2846904	17294
124	u83XiGXfhX5Hol0vhA	Mfge8	Mfge8	ILMN_2771034	17304
125	rk69IpQevzewvv9wQU	Mmp12	Mmp12	ILMN_1250421	17381
126	TnIisiqt7m869K0h7s	Mmp9	Mmp9	ILMN_2711075	17395
127	lxos_h0Tp93hUQX1eg	Myl4	Myl4	ILMN_2610744	17896
128	ESdC5ZVdFlD3Qevluw	Nedd4l	Nedd4l	ILMN_2878501	83814
129	WoiQ9Hu3pu6CCKteCQ	Nedd9	Nedd9	ILMN_2654186	18003
130	BXSKiToPsMuEXGNZOg	Net1	Net1	ILMN_2610771	56349
131	uVX_3Qeg4EBFbpLdHk	Net1	Net1	ILMN_3151722	56349
132	cSScjt6EqBO_v1y78Y	Nt5e	Nt5e	ILMN_2813830	23959
133	cR0N1ViJ7w6LfV5P78	Nup210	Nup210	ILMN_1257579	54563
134	xfhbvvt3yPB7niHVrI	Olfm1	Olfm1	ILMN_1240615	56177
135	TnM1JfVImKS7s4sWWY	Oplah	Oplah	ILMN_2662191	75475
136	36IJxIEaq_eOjFS5p4	Palld	Palld	ILMN_3092653	72333
137	3OP_Izh18nR79eqdSk	Paqr7	Paqr7	ILMN_1222036	71904
138	BkRuB6F8qQnn4iL3Yc	Paqr9	Paqr9	ILMN_2752524	75552
139	xqRAhEBWqfBWchDk6A	Paqr9	Paqr9	ILMN_3094043	75552
140	iwg0glTgZHggQku8l4	Pcolce2	Pcolce2	ILMN_1238603	76477
141	HppPfFoi3o.PpLgr.U	Pcolce2	Pcolce2	ILMN_2678421	76477
142	NroTT0kgQIiugIvp5U	Pde1a	Pde1a	ILMN_3146952	18573
143	Er0qvRKaHlxP15.VyA	Pfkfb4	Pfkfb4	ILMN_2712668	270198
144	WVTrzr15x7dQQ7lq9Y	Pfkp	Pfkp	ILMN_1237695	56421
145	l6Xvqz8nXp.N2.lehc	Acer3	Phca	ILMN_2681057	66190
146	u3qJzCX3p93HuRf744	Phkg1	Phkg1	ILMN_2769795	18682
147	BIPp1PoCHdefTFiftk	Pik3r1	Pik3r1	ILMN_2473531	18708
148	lTFiftlOIyT6eVKOec	Pik3r1	Pik3r1	ILMN_3114641	18708
149	clMewrlxRLjd5wcODk	Pon1	Pon1	ILMN_2676379	18979
150	0S5edLLh.3T4EqdxHI	Ppl	Ppl	ILMN_3155363	19041
151	WbUQ4p3UBU0cd9VAkc	Prc1	Prc1	ILMN_2757125	233406
152	WECGi5zTx1UJIiAFFI	Prlr	Prlr	ILMN_2868699	19116
153	To8Xo1xufTkdf0eInI	Prlr	Prlr	ILMN_2617005	19116
154	QOk99Oi6HX241mDmXU	Prtn3	Prtn3	ILMN_2758029	19152
155	fSSK0gpxI1Nc1NbxOk	Ptprd	Ptprd	ILMN_2501929	19266

156	HuV4x1JIrSCnEjU1zU	Ptprd	Ptprd	ILMN_3103904	19266
157	6Bf.A4l4xe79JP6Ub4	Ptpre	Ptpre	ILMN_2826916	19267
158	NdVTXc6V2jhlPfegOc	Pvalb	Pvalb	ILMN_1218223	19293
159	HXenoTnnL3v6df9zUo	Rab7l1	Rab7l1	ILMN_2681186	226422
160	fbZuXex_91Ju59ViDM	Rasgrf1	Rasgrf1	ILMN_1233146	19417
161	3tm5d7H73Um7n1WIMw	Rasgrf1	Rasgrf1	ILMN_2699663	19417
162	Em577sg7vgd_Q3X1fo	Rgs1	Rgs1	ILMN_2625377	50778
163	fg7qSK6U_iPvsd4otI	Ripk4	Ripk4	ILMN_2840856	72388
164	0ClAZ.epZNunZBJZCM	Rrad	Rrad	ILMN_1219106	56437
165	Kl7mXoxHl0.9BCZAfY	Mtus1	scl33870.2_144	ILMN_2459676	102103
166	9NWdNt3eUG.7gBwCDU	Sema5a	Sema5a	ILMN_2604224	20356
167	ZVnTbd3lBv_4AcAg1w	Sema5a	Sema5a	ILMN_2604226	20356
168	ENKH_CgN1JGehUqR1o	Sept9	Sept9	ILMN_2602185	53860
169	lUoKaWsn0uyXeQwCJ8	Serpinf1	Serpinf1	ILMN_2639239	20317
170	rJ7l_XZd7UZUfpdF9E	Sfrp5	Sfrp5	ILMN_1232779	54612
171	3l3EI328ItCeWtKLeE	Sfxn1	Sfxn1	ILMN_1233606	14057
172	9Sc0s8mK7fljNItLu0	Sfxn1	Sfxn1	ILMN_2675569	14057
173	3BKvfBFBTn3XI5ehUQ	Sirpb1a	Sirpb1	ILMN_3154691	320832
174	NVt4_n.itd94n_.Snc	Slamf9	Slamf9	ILMN_2663249	98365
175	WRJbXv3ej562c4ih2U	Slc15a3	Slc15a3	ILMN_2987709	65221
176	ZVqd5c7uxUe6HqAfR8	Slc1a3	Slc1a3	ILMN_2634317	20512
177	HdwkxN3NS0ot357SK4	Slc2a13	Slc2a13	ILMN_2925424	239606
178	6HSPnOgKp.nqAph13A	Slc2a3	Slc2a3	ILMN_2616565	20527
179	HeV7LNUOjNHVLdA_p4	Slc44a1	Slc44a1	ILMN_1241827	100434
180	BCEAi4P_VB003uV9TY	Slc5a7	Slc5a7	ILMN_1243388	63993
181	9pUi1_1Ls_wIlF2iXo	Sik2	Snf1lk2	ILMN_1232372	235344
182	Kcs_FcINeIlTM7.U4k	Spc25	Spc25	ILMN_1255960	66442
183	lNNUBTl3_KwT_4ngBI	Spc25	Spc25	ILMN_2901180	66442
184	cp0UGek5_K.cfsAIKk	Stambpl1	Stambpl1	ILMN_1252400	76630
185	3urLUy_XoBUyyRAl1U	Steap2	Steap2	ILMN_2797726	74051
186	HdBm3_.F.QVXj0Cjpc	N/A	Synpo2	ILMN_2678838	N/A
187	foV3nu3uG_suXuNu74	Syp	Syp	ILMN_2630182	20977
188	oFKGRaWBuie7YjPlSw	Tekt1	Tekt1	ILMN_1239718	21689
189	fuURItSCr.v8LCcoOI	Thbd	Thbd	ILMN_1249767	21824
190	u3ihxSJbR.n5eUaDUo	Timp1	Timp1	ILMN_3103896	21857
191	rcUiW0f5_XlGg1KfuQ	Timp1	Timp1	ILMN_2769918	21857
192	lKiX1Cu1D5XN3q3qkg	Tnfrsf12a	Tnfrsf12a	ILMN_2424299	27279
193	6Sd1_MVEB83uX0l0uk	Tpcn2	Tpcn2	ILMN_1236133	233979
194	9Kj_fS8reOI7lOtMJQ	Tph2	Tph2	ILMN_2460179	216343
195	H0_UblkKq0eXtG_V6c	Tst	Tst	ILMN_2493175	22117

196	iqjhRqjmuOIJ0CWP10	Ubd	Ubd	ILMN_2426853	24108
197	reORWLyxdHH.pXv9d8	Ugt3a2	Ugt3a2	ILMN_2658355	223337
198	Qv50TwY14KC14QtFP4	Upk1b	Upk1b	ILMN_2936646	22268
199	rgXkn3K4f6SP_XmgpM	Wfdc1	Wfdc1	ILMN_2466164	67866
200	HIllOkuc8CXdM3_AF0	Xpnpep2	Xpnpep2	ILMN_1248998	170745

7. Supplementary Table 7: Correlations of liver and adipose TDIs with individual PDIs

The table lists the Spearman correlations and their respective p-values between liver and adipose TDIs and the PDIs of individual physiological markers. P-values were adjusted to multiple hypotheses testing by the Benjamini-Hochberg (BH) method. Refer to Supplementary Table 1 for complete details concerning the physiological markers.

		Adipose		Liver	
	**Physiological marker**	**Spearman rho**	**BH-adjusted p-value**	**Spearman rho**	**BH-adjusted p-value**
1	Body weight	0.84	2.6e-17	0.34	2.9e-04
2	Liver weight	0.16	1.2e-01	0.52	1.4e-08
3	Heart weight	0.37	2.1e-03	0.04	3.9e-01
4	Visceral WAT	0.75	7.2e-12	0.32	6.0e-04
5	Gonadal WAT	0.84	< 1e-17	0.27	2.8e-03
6	Subcutaneous WAT	0.84	2.6e-17	0.31	8.0e-04
7	Total WAT (visceral + gonadal + subcutaneous)	0.85	< 1e-17	0.28	2.7e-03
8	Ratio visceral / subcutaneous WAT	0.59	5.3e-07	0.22	1.4e-02
9	Kidneys weight (total both kidneys)	0.01	4.7e-01	0.29	1.9e-03
10	Liver triglycerides	0.54	4.8e-06	0.61	5.5e-12
11	Atherosclerotic lesion area	0.19	1e-01	0.43	1.2e-05
12	Urine glucose	0.18	1.1e-01	0.27	3.8e-03
13	Plasma cholesterol	0.59	5.3e-07	0.76	2.5e-21
14	Plasma triglycerides	0.50	3.3e-05	0.64	1.8e-13
15	Plasma glucose	0.38	1.6e-03	0.15	6.6e-02
16	Plasma insulin	0.70	3.4e-10	0.42	1.2e-05
17	Plasma glucagon	0.06	3.5e-01	0	5.1e-01
18	Plasma E-selectin	0.39	1.4e-03	0.16	6.6e-02
19	Plasma VCAM	0.50	2.5e-05	0.38	6.5e-05
20	Plasma MCP-1	0.06	3.5e-01	0.14	9.2e-02
21	Plasma adiponectin	0.50	2.8e-05	0.02	4.4e-01
22	Plasma leptin	0.86	2.6e-17	0.36	2.3e-04
23	Plasma resistin	0.67	1.1e-08	0.09	2.1e-01
24	HOMA insulin resistance	0.68	1.9e-09	0.37	7.0e-05
25	QUICKI insulin resistance	0.60	5.3e-07	0.40	3.4e-05
26	ACR (urine albumin / creatinine ratio)	0.76	2.4e-10	0.27	5.5e-03

8. <u>Supplementary Table 8: Correlations of liver MDI with individual PDIs</u>

The table lists the Spearman correlations and their respective p-values between liver MDI and the PDIs of individual physiological markers. P-values were adjusted to multiple hypotheses testing by the Benjamini-Hochberg (BH) method. Refer to Supplementary Table 1 for complete details concerning the physiological markers.

	**Physiological marker**	**Spearman rho**	**BH-adjusted p-value**
1	Body weight	0.12	2.5e-01
2	Liver weight	0.49	5.1e-07
3	Heart weight	0.11	2.5e-01
4	Visceral WAT	0.02	5.1e-01
5	Gonadal WAT	0	5.4e-01
6	Subcutaneous WAT	0.12	2.5e-01
7	Total WAT (visceral + gonadal + subcutaneous)	0.02	5.1e-01
8	Ratio visceral / subcutaneous WAT	0.12	2.5e-01
9	Kidneys weight (total both kidneys)	0.35	5.8e-04
10	Liver triglycerides	0.31	2.4e-03
11	Atherosclerotic lesion area	0.16	1.7e-01
12	Urine glucose	0.09	3.2e-01
13	Plasma cholesterol	0.42	3.5e-05
14	Plasma triglycerides	0.36	3.4e-04
15	Plasma glucose	0	5.5e-01
16	Plasma insulin	0.12	2.5e-01
17	Plasma glucagon	0.05	4.3e-01
18	Plasma E-selectin	0.30	3.2e-03
19	Plasma VCAM	0.09	3.2e-01
20	Plasma MCP-1	-0.11	8.7e-01
21	Plasma adiponectin	0.01	5.1e-01
22	Plasma leptin	0.07	3.42e-01
23	Plasma resistin	0.08	3.42e-01
24	HOMA insulin resistance	0.07	3.42e-01
25	QUICKI insulin resistance	0.07	3.42-01
26	ACR (urine albumin / creatinine ratio)	0.24	3.5e-02

9. Supplementary Table 9: Adverse side-effects of drugs in the current study

The following is a comprehensive list of adverse side-effects of the drugs included in the current study as stated in the package inserts provided by the manufacturers. Highlighted in bold letters are side-effects which were investigated or could have been inferred from the physiological data collected in the animal model studied here. Superscript numbers refer to the physiological parameters relevant to the highlighted side effects as follows: 1. Body weight 2. Plasma glucose 3. Liver weight 4. Liver triglycerides 5. Heart weight 6. Atherosclerotic lesion area 7. Plasma cholesterol 8. Kidneys weight 9. ACR (urine albumin/ creatinine ratio).

Side-effects data was downloaded from the SIDER database (http://sideeffects.embl.de/; accessed December 2014; (Kuhn *et al*, 2010)), which aggregates package-insert information from several public sources, among which are the US Food and Drug Administration (FDA) and Health Canada. SIDER lists side-effects using the Medical Dictionary for Regulatory Activities (MedDRA) – a standardized medical terminology that facilitates sharing of information concerning medical products (see http://www.meddra.org/; accessed December 2014).

Note, however, that the side-effects listed here are ones that pertain to human patients and may not apply to the mouse model studied here. In addition, the list is comprehensive and includes rare side-effects that may not be observed in a limited-scale study even if they occur in the animal model.

Drug	***PubChem Compound ID***	***Adverse side-effects***
metformin	4091	Abdominal discomfort, Abdominal distension, Abdominal pain, Abdominal pain upper, Abnormal faeces, Abscess, **Acute prerenal failure**[8,9], Anaemia, Anaemia megaloblastic, **Angina pectoris**[6], **Angina unstable**[6], **Angiopathy**[6], Aortic dissection, Asthenia, **Azotaemia**[8,9], Blood disorder, **Blood glucose decreased**[2], Breast disorder, **Cardiac disorder**[5,6], Chest discomfort, Chest pain, Chills, Connective tissue disorder, Constipation, Decreased appetite, Dehydration, Dermatitis, Diarrhoea, Discomfort, Dizziness, Dysgeusia, Dyspepsia, Dyspnoea, Ear pain, Emotional distress, Epigastric discomfort, Erythema, Eye disorder, Fatigue, Feeling abnormal, Flatulence, Flushing, Fungal infection, Gastric disorder, Gastroenteritis, Gastrointestinal disorder, Gastrointestinal pain, Gastrointestinal tract irritation, Headache, **Hepatitis**[3,4], Hyperhidrosis, Hypertension, Hypoaesthesia, **Hypoglycaemia**[2], Ill-defined disorder, Immune system disorder, Infection, Infestation, Influenza, Lactic acidosis, Lethargy, **Liver function test abnormal**[3,4], Loss of consciousness, Malaise, Mediastinal disorder, Migraine, Muscle spasms, Musculoskeletal discomfort, Myalgia, Nail disorder, Nasal congestion, Nasopharyngitis,

		Nausea, Nervous system disorder, Neuropathy peripheral, Neutropenia, Oedema, Oedema peripheral, Pain, Pain in extremity, Palpitations, Pancreatitis, Paraesthesia, Pruritus, Rash, Respiratory disorder, Respiratory tract infection, Rhinitis, Rhinitis seasonal, Rhinorrhoea, Seasonal allergy, Shock, Sinus congestion, Sinus headache, Somnolence, Syncope, Thrombocytopenia, Tonsillitis, Tooth abscess, Toothache, Tremor, Upper respiratory tract infection, Urticaria, Viral diarrhoea, Vision blurred, Vomiting, White blood cell count increased
glibenclamide	3488	Abdominal distension, Abdominal pain, Agranulocytosis, Anaemia, Angioedema, Aplastic anaemia, Arthralgia, Asthenia, Cholestasis, Diarrhoea, Dyspepsia, Dyspnoea, Erythema, Erythropenia, Gastrointestinal disorder, Gastrointestinal pain, Haemolytic anaemia, **Hepatic failure**[3,4], **Hepatic function abnormal**[3,4], **Hepatitis**[3,4], Hypersensitivity, Hyponatraemia, Hypopituitarism, Hypotension, Inappropriate antidiuretic hormone secretion, Jaundice, Jaundice cholestatic, Leukocytoclastic vasculitis, Leukopenia, **Liver disorder**[3,4], Malnutrition, Musculoskeletal discomfort, Myalgia, Nausea, **Nephropathy**[8,9], Nervous system disorder, Pain, Pancytopenia, Photosensitivity reaction, Porphyria, Porphyria non-acute, Pruritus, Purpura, Rash, Shock, Thrombocytopenia, Urticaria, Vascular purpura, Vasculitis, Vision blurred, Visual impairment, Vomiting
sitagliptin	4369359	Abdominal discomfort, Abdominal pain, Abdominal pain upper, Anaphylactic shock, **Angina pectoris**[6], Angioedema, **Angiopathy**[6], Anxiety, Aortic dissection, Arthralgia, Asthenia, Back pain, **Blood glucose decreased**[2], **Blood glucose increased**[2], Breast disorder, Bronchitis, Bundle branch block, **Cardiac disorder**[5,6], Cellulitis, Chest pain, Connective tissue disorder, Constipation, Cough, Decreased appetite, Dermatitis, Diarrhoea, Discomfort, Dizziness, Dysmenorrhoea, Dyspepsia, Eczema, Erectile dysfunction, Eye disorder, Face oedema, Fatigue, Feeling abnormal, Flatulence, Gastric ulcer, Gastritis, Gastroenteritis, Gastrointestinal disorder, Gastrointestinal pain, Gastrooesophageal reflux disease, Headache, Helicobacter gastritis, **Hepatic steatosis**[3,4], Hepatobiliary disease, Herpes virus infection, Herpes zoster, **Hyperglycaemia**[2], Hypersensitivity, Hypertension, Hypoaesthesia, **Hypoglycaemia**[2], Hypotension, Ill-defined disorder, Infection, Infestation, Influenza, Insomnia, Malaise, Mediastinal disorder, Mental disorder, Migraine, Muscle spasms, Muscle tightness, Musculoskeletal discomfort, Musculoskeletal pain, Myalgia, Nasopharyngitis, Nausea,

		Neck pain, Nephrolithiasis, Nervous system disorder, Neuropathy peripheral, Oedema, Oedema peripheral, Oesophagitis, Oropharyngeal pain, Orthostatic hypotension, Osteoarthritis, Pain, Pain in extremity, Parosmia, Pharyngitis, Pneumonia, Rash, Reflux oesophagitis, Respiratory tract infection, Retching, Sciatica, Sinus congestion, Sinus headache, Sinusitis, Skin disorder, Somnolence, Tooth infection, Toothache, Tracheobronchitis, Ulcer, Upper respiratory tract infection, Urethral disorder, **Urinary tract disorder**[8,9], Urinary tract infection, Urticaria, Vertigo, Viral diarrhoea, Vision blurred, Vomiting, **Weight increased**[1]
rosiglitazone	77999	Abdominal pain, **Acute coronary syndrome**[6], Alanine aminotransferase increased, Anaemia, Anaphylactic shock, Angioedema, Arthralgia, Asthenia, Back pain, **Cardiac disorder**[5,6], **Cardiac failure**[5,6], **Cardiac failure congestive**[5,6], Constipation, Cough, Dermatitis, Diabetic retinal oedema, Diarrhoea, Dizziness, Dysmenorrhoea, Effusion, Fatigue, Gastrointestinal pain, Headache, **Hepatic failure**[3,4], **Hepatitis**[3,4], Hyperbilirubinaemia, **Hyperglycaemia**[2], Hypertension, **Hypoglycaemia**[2], Increased appetite, **Infarction**[6], Influenza, **Ischaemia**[6], Ketonuria, Macular oedema, Musculoskeletal discomfort, **Myocardial infarction**[6], **Myocardial ischaemia**[6], Nasopharyngitis, Nausea, Oedema, Oropharyngeal pain, Pain, Pleural disorder, Pleural effusion, Pruritus, Pulmonary oedema, Rash, Respiratory tract infection, Sinusitis, Social avoidant behaviour, Upper respiratory tract infection, Urticaria, Visual acuity reduced, Volume blood increased, Vomiting, **Weight increased**[1]
pioglitazone	4829	**Acute coronary syndrome**[6], Anaemia, Back pain, Bladder cancer, Bladder neoplasm, Blood creatine phosphokinase increased, Chest pain, **Diabetes mellitus**[2], Diabetic retinal oedema, Diarrhoea, Disease progression, Dizziness, Electrocardiogram abnormal, Flatulence, Fluid retention, Gamma-glutamyltransferase decreased, Generalised oedema, Headache, **Hyperglycaemia**[2], Hypoaesthesia, **Ischaemia**[6], **Liver function test abnormal**[3,4], Musculoskeletal discomfort, Myalgia, **Myocardial ischaemia**[6], Neoplasm, Neoplasm malignant, Oedema, Pain in extremity, Pharyngitis, Respiratory tract infection, Sinusitis, Social avoidant behaviour, Tooth disorder, Upper respiratory tract infection, Urinary tract infection, Visual impairment
fenofibrate	3339	Abdominal pain, Acne, **Acute coronary syndrome**[6], Alanine aminotransferase increased, Alopecia, Alveolitis, Amblyopia, Anaemia, **Angina pectoris**[6], Anorectal disorder, Anxiety, Arrhythmia, Arthralgia, Arthritis, Arthropathy, Aspartate

		aminotransferase increased, Asthenia, Asthma, Atrial fibrillation, Back pain, Bladder pain, Blindness, Blood alkaline phosphatase increased, Blood bilirubin increased, Blood creatine phosphokinase increased, **Blood creatinine increased**[8,9], Blood lactate dehydrogenase increased, Blood urea increased, Blood uric acid increased, Body temperature increased, Bronchitis, Bursitis, Calculus urinary, Candidiasis, Cardiac fibrillation, **Cardiovascular disorder**[5,6], Cataract, Chest pain, Cholecystitis, Cholelithiasis, Colitis, Conjunctivitis, Constipation, Coronary artery disease, Cough, Cyst, Cystitis, Cystitis noninfective, Decreased appetite, Deep vein thrombosis, Dermatitis, Dermatitis contact, Diarrhoea, Discomfort, Disturbance in sexual arousal, Dizziness, Dry mouth, Duodenal ulcer, Dyspepsia, Dyspnoea, Dysuria, Ear infection, Ear pain, Ecchymosis, Eczema, Electrocardiogram abnormal, Embolism, Eosinophilia, Erectile dysfunction, Eructation, Erythema, Extrasystoles, Eye disorder, Fatigue, Feeling abnormal, Flatulence, Fungal skin infection, Gamma-glutamyltransferase increased, Gastritis, Gastroenteritis, Gastrointestinal disorder, Gastrointestinal pain, Gout, Gynaecomastia, Haemoglobin, Haemorrhage, Haptoglobin decreased, Headache, **Hepatic cirrhosis**[3,4], **Hepatic function abnormal**[3,4], **Hepatic steatosis**[3,4], **Hepatitis**[3,4], Hernia, Herpes simplex, Herpes virus infection, Herpes zoster, Hyperhidrosis, Hypersensitivity, Hypertonia, Hyperuricaemia, **Hypoglycaemia**[2], Hypotension, Ill-defined disorder, Increased appetite, **Infarction**[6], Infection, Influenza, Insomnia, Jaundice, **Laboratory test abnormal**[2,7,5], Laryngitis, Leukopenia, Libido decreased, **Liver function test abnormal**[3,4], Lymphadenopathy, Malaise, Migraine, Multiple hereditary exostosis, Muscle spasms, Muscular weakness, Musculoskeletal discomfort, Myalgia, **Myocardial infarction**[6], Myositis, Nail disorder, Nausea, Nervousness, Neuralgia, Oedema, Oedema peripheral, Oesophagitis, Osteoarthritis, Otitis media, Pain, Palpitations, Paraesthesia, Peptic ulcer, Pharyngitis, Phlebitis, Photosensitivity reaction, Pneumonia, Pollakiuria, Prostatic disorder, Pruritus, Pulmonary embolism, Rash, Rash maculo-papular, Rectal haemorrhage, Refraction disorder, **Renal failure**[8,9], **Renal failure acute**[8,9], **Renal impairment**[8,9], Respiratory disorder, Rhabdomyolysis, Rhinitis, Sinusitis, Skin disorder, Skin ulcer, Somnolence, Tachycardia, Tenosynovitis, Tension, Thrombocytopenia, Thrombosis, Tooth disorder, Ulcer, Unintended pregnancy, Urticaria, Varicose vein, Vasodilation procedure, Venous thrombosis, Ventricular extrasystoles, Vertigo, Visual acuity reduced, Visual impairment, Vomiting, Vulvovaginal candidiasis, Vulvovaginal mycotic infection,

		**Weight increased**[1]
T0901317	447912	(*) This compound was never approved for use in humans, and therefore has no SIDER entry. There exist, however, multiple reports that implicate LXR agonists, including T0901317, in impaired lipid metabolism and hepatic dysfunction (Schultz *et al*, 2000; Chisholm *et al*, 2003; Lund *et al*, 2003; Millatt *et al*, 2003; Zanotti *et al*, 2008; Jung *et al*, 2011). Administration of LXR-623, a synthetic LXR agonist, to healthy human subjects led to neurological and psychiatric adverse events (Katz *et al*, 2009).<br><br>While the physiological data studied here allows detection of adverse treatment outcomes related to disruption of lipid metabolism and liver functions, it is not rich enough to study adverse events related to the central nervous system.
atorvastatin	60822 (atorvastatin calcium)	Abdominal discomfort, Abdominal pain, Abnormal dreams, Acne, Affect lability, Ageusia, Aggression, Agitation, Alanine aminotransferase increased, Alopecia, Amblyopia, Amnesia, Anaemia, Anaphylactic shock, Angioedema, Anorectal discomfort, Arrhythmia, Arthralgia, Arthritis, Asthenia, Asthma, Back pain, Biliary colic, Bladder pain, Blood creatine phosphokinase increased, Blood magnesium decreased, Body temperature increased, Breast enlargement, Bronchitis, Bursitis, Cells in urine, Cerebral haemorrhage, Cheilitis, Chest pain, Cholestasis, Colitis, Constipation, Coordination abnormal, Cystitis, Cystitis noninfective, Deafness, Death, Decreased appetite, Dermatitis, Dermatitis contact, Diarrhoea, Discomfort, Disturbance in sexual arousal, Dizziness, Dry eye, Dry mouth, Dry skin, Duodenal ulcer, Dysgeusia, Dyspepsia, Dysphagia, Dyspnoea, Dysuria, Ecchymosis, Eczema, Ejaculation disorder, Enteritis, Epididymitis, Epistaxis, Erectile dysfunction, Eructation, Erythema, Erythema multiforme, Eye haemorrhage, Face oedema, Fatigue, Feeling abnormal, Fibrocystic breast disease, Flatulence, Fluid retention, Gastric disorder, Gastric ulcer, Gastritis, Gastroenteritis, Gastrointestinal pain, Generalised oedema, Gingival bleeding, Glaucoma, Glossitis, Gout, Haematuria, Haemoglobin, Haemorrhage, Haemorrhagic stroke, Headache, **Hepatic failure**[3,4], **Hepatitis**[6], **Hyperglycaemia**[2], Hyperhidrosis, Hyperkinesia, Hypersensitivity, Hypertonia, Hypoaesthesia, **Hypoglycaemia**[2], Hypotension, Ill-defined disorder, Incontinence, Increased appetite, Infection, Influenza, Insomnia, **Ischaemic stroke**[6], Jaundice, Jaundice cholestatic, Joint swelling, Libido decreased, **Liver function test abnormal**[3,4], Loss of consciousness, Lymphadenopathy,

		Malaise, Melaena, Memory impairment, Metrorrhagia, Micturition urgency, Migraine, Mood swings, Mouth ulceration, Muscle contracture, Muscle fatigue, Muscle rigidity, Muscle spasms, Muscular weakness, Musculoskeletal discomfort, Musculoskeletal pain, Musculoskeletal stiffness, Myalgia, Myopathy, Myositis, Nasopharyngitis, Nausea, Neck pain, Nephritis, Nephrolithiasis, Neuropathy peripheral, Nightmare, Nocturia, Nuchal rigidity, Oedema, Oedema peripheral, Oesophagitis, Oropharyngeal pain, Orthostatic hypotension, Pain, Pain in extremity, Palpitations, Pancreatitis, Paraesthesia, Paralysis, Parosmia, Petechiae, Pharyngitis, Phlebitis, Photosensitivity reaction, Pneumonia, Pollakiuria, Proctalgia, Pruritus, Rash, Rectal haemorrhage, Rectal tenesmus, Refraction disorder, Rhabdomyolysis, Rhinitis, Seborrhoeic dermatitis, Shock, Sinusitis, Skin ulcer, Somnolence, Stomatitis, Swelling, Syncope, Tendinous contracture, Tenosynovitis, Thrombocytopenia, Tinnitus, Torticollis, Toxic epidermal necrolysis, **Transient ischaemic attack**[6], Ulcer, Urinary incontinence, **Urinary retention**[8], Urinary tract infection, Urticaria, Uterine haemorrhage, VIIth nerve paralysis, Vaginal haemorrhage, Vasodilation procedure, Vision blurred, Vomiting, **Weight increased**[1], White blood cells urine positive
salicylate	2244 (acetylsalicylic acid)	(*) Salicylate is not listed in SIDER; we provide here the information for its artificial derivative acetylsalicylic acid (aspirin).<br><br>Anaemia, Anaphylactic shock, Angioedema, Asthma, Confusional state, Deafness, Diarrhoea, Dyspepsia, Feeling abnormal, Haematemesis, Hearing impaired, Hyperhidrosis, Hypersensitivity, Leukopenia, Melaena, Nausea, Oedema, Pruritus, Purpura, Rash, Somnolence, Thirst, Thrombocytopenia, Tinnitus, Ulcer, Urticaria, Vascular purpura, Vertigo, Vomiting
rofecoxib	5090	Abdominal distension, Abdominal pain, Abdominal pain upper, Abdominal tenderness, Abscess, **Acute coronary syndrome**[6], Agranulocytosis, Alopecia, Alveolar osteitis, Alveolitis, Anaemia, Analgesic therapy, Anaphylactic shock, Anaphylactoid reaction, **Angina pectoris**[6], **Angina unstable**[6], Angioedema, Anxiety, Aphthous stomatitis, Aplastic anaemia, Arrhythmia, Arthralgia, Arthropathy, Asthenia, Asthma, Atopy, Atrial fibrillation, Back pain, Basal cell carcinoma, Bladder pain, Blister, Body temperature increased, Bradycardia, Breast cancer, Breast mass, Bronchitis, Bronchospasm, Bursitis, Calculus urinary, **Cardiac**

		**failure**[5,6], **Cardiac failure congestive**[5,6], Cardiac fibrillation, Cellulitis, **Cerebrovascular accident**[6], Cerumen impaction, Chest pain, Chills, Cholecystitis, Colitis, Confusional state, Congenital anomaly, Conjunctivitis, Constipation, Cough, Cyst, Cystitis, Cystitis noninfective, Death, Deep vein thrombosis, Dental caries, Dermatitis, Dermatitis atopic, Dermatitis bullous, Dermatitis contact, Developmental delay, Diaphragmatic hernia, Diarrhoea, Disability, Discomfort, Dizziness, Dry mouth, Dry throat, Duodenal perforation, Duodenal ulcer, Dysgeusia, Dyspepsia, Dyspnoea, Dysuria, Ear infection, Embolism, Epigastric discomfort, Epilepsy, Epistaxis, Erythema, Fatigue, Feeling abnormal, Flatulence, Fluid retention, Flushing, Fungal infection, Gastric disorder, Gastric perforation, Gastric ulcer, Gastritis, Gastroenteritis, Gastrointestinal disorder, Gastrointestinal haemorrhage, Gastrointestinal pain, Gastrooesophageal reflux disease, Haematochezia, Haematoma, Haemoglobin, Haemorrhage, Haemorrhoids, Hallucination, Heart rate irregular, **Hepatic failure**[3,4], **Hepatitis**[3,4], Hernia, Herpes simplex, Herpes virus infection, Herpes zoster, **Hypercholesterolaemia**[7], Hyperhidrosis, Hyperkalaemia, Hypersensitivity, Hypertension, Hypertensive crisis, Hypoaesthesia, Hyponatraemia, **Infarction**[6], Infection, Influenza, Insomnia, Intestinal obstruction, Jaundice, Joint swelling, Laryngitis, Leukocytoclastic vasculitis, Leukopenia, Loss of consciousness, Lymphoma, Meningitis, Meningitis aseptic, Menopausal symptoms, Menopause, Menstrual disorder, Mental disorder, Mouth ulceration, Muscle spasms, Muscular weakness, Musculoskeletal discomfort, Musculoskeletal pain, Musculoskeletal stiffness, Myalgia, **Myocardial infarction**[6], Myopathy, Nasal congestion, Nausea, Neoplasm, Neoplasm malignant, Nephritis, Neuropathy peripheral, Nocturia, Obstruction, Oedema, Oedema peripheral, Oesophageal disorder, Oesophageal ulcer, Oesophagitis, Oral disorder, Oral infection, Osteoarthritis, Otitis media, Pain in extremity, Palpitations, Pancreatitis, Pancytopenia, Paraesthesia, Pelvic pain, Pharyngitis, Photosensitivity reaction, Pneumonia, Procedural pain, Pruritus, Pulmonary congestion, Pulmonary embolism, Pulmonary oedema, Rash, **Renal failure**[8,9], **Renal failure acute**[8,9], **Renal failure chronic**[8,9], Respiratory tract congestion, Respiratory tract infection, Rhinitis, Rhinitis allergic, Sciatica, Shock, Sinusitis, Somnolence, Stomatitis, Swelling, Syncope, Tachycardia, Tenderness, Tendonitis, Thrombocytopenia, Thrombosis, Tinnitus, Tonsillitis, Tooth impacted, Toothache, Toxic epidermal necrolysis, **Transient**

		**ischaemic attack**[6], **Tubulointerstitial nephritis**[8,9], Ulcer, Upper respiratory tract infection, **Urinary retention**[8], Urinary tract infection, Urticaria, Vaginal infection, Vaginal inflammation, Vasculitis, Venous insufficiency, Venous thrombosis, Ventricular extrasystoles, Vertigo, Viral diarrhoea, Viral infection, Vision blurred, Vomiting, **Weight increased**[1], Xerosis

Supplementary References

Ahmadian M, Suh JM, Hah N, Liddle C, Atkins AR, Downes M & Evans RM (2013) PPARγ signaling and metabolism: the good, the bad and the future. *Nat Med* **99:** 557–566

American Diabetes Association (2013) Standards of Medical Care in Diabetes—2013. *Diabetes Care* **36:** S11–S66

Awtry EH & Loscalzo J (2000) Aspirin. *Circ.* **101 :** 1206–1218

Baron SH (1982) Salicylates as hypoglycemic agents. *Diabetes Care* **5:** 64–71

Berg AH & Scherer PE (2005) Adipose Tissue, Inflammation, and Cardiovascular Disease. *Circ. Res.* **96 :** 939–949

Campbell C, Smyth S, Montalescot G & SR S (2007) Aspirin dose for the prevention of cardiovascular disease: A systematic review. *JAMA* **297:** 2018–2024

Chisholm JW, Hong J, Mills SA & Lawn RM (2003) The LXR ligand T0901317 induces severe lipogenesis in the db/db diabetic mouse. *J. Lipid Res.* **44 :** 2039–2048

Escher P, Braissant O, Basu-Modak S, Michalik L, Wahli W & Desvergne B (2001) Rat PPARs: Quantitative Analysis in Adult Rat Tissues and Regulation in Fasting and Refeeding. *Endocrinology* **142:** 4195–4202

Evans RM, Barish GD & Wang Y-X (2004) PPARs and the complex journey to obesity. *Nat Med* **10:** 355–361

Forcheron F, Cachefo A, Thevenon S, Pinteur C & Beylot M (2002) Mechanisms of the Triglyceride- and Cholesterol-Lowering Effect of Fenofibrate in Hyperlipidemic Type 2 Diabetic Patients. *Diabetes* **51 :** 3486–3491

Foretz M, Hébrard S, Leclerc J, Zarrinpashneh E, Soty M, Mithieux G, Sakamoto K, Andreelli F & Viollet B (2010) Metformin inhibits hepatic gluconeogenesis in mice independently of the LKB1/AMPK pathway via a decrease in hepatic energy state. *J. Clin. Invest.* **120:** 2355–2369

Frantz B, O'Neill EA, Ghosh S & Kopp E (1995) The Effect of Sodium Salicylate and Aspirin on NF-κB. *Science (80-.).* **270:** 2017–2019

Furst DE, Ulrich RW & Prakash S (2012) Nonsteroidal Anti-Inflammatory Drugs, Disease-Modifying Antirheumatic Drugs, Nonopioid Analgesics, & Drugs Used in Gout. In *Basic & Clinical Pharmacology (12th ed.)*, Katzung BG Masters SB & Trevor AJ (eds) New York, NY: McGraw-Hill

Gilgore SG (1960) The Influence of Salicylate on Hyperglycemia. *Diabetes* **9 :** 392–393

Gilgore SG & Rupp JJ (1962) The long-term response of diabetes mellitus to salicylate therapy: Report of a case. *JAMA* **180:** 65–66

Goldfine AB, Buck JS, Desouza C, Fonseca V, Chen Y-DI, Shoelson SE, Jablonski KA, Creager MA & Team for the T-F (Targeting IUS in T 2 D-MDAS (2013) Targeting Inflammation Using Salsalate in Patients With Type 2 Diabetes: Effects on Flow-Mediated Dilation (TINSAL-FMD). *Diabetes Care* **36 :** 4132–4139

Hawley SA, Fullerton MD, Ross FA, Schertzer JD, Chevtzoff C, Walker KJ, Peggie MW, Zibrova D, Green KA, Mustard KJ, Kemp BE, Sakamoto K, Steinberg GR & Hardie DG (2012) The Ancient Drug Salicylate Directly Activates AMP-Activated Protein Kinase. *Sci.* **336 :** 918–922

Hong C & Tontonoz P (2014) Liver X receptors in lipid metabolism: opportunities for drug discovery. *Nat Rev Drug Discov* **13:** 433–444

Hundal RS, Petersen KF, Mayerson AB, Randhawa PS, Inzucchi S, Shoelson SE & Shulman GI (2002) Mechanism by which high-dose aspirin improves glucose metabolism in type 2 diabetes. *J. Clin. Invest.* **109:** 1321–1326

Iorio F, Bosotti R, Scacheri E, Belcastro V, Mithbaokar P, Ferriero R, Murino L, Tagliaferri R, Brunetti-Pierri N, Isacchi A & di Bernardo D (2010) Discovery of drug mode of action and drug repositioning from transcriptional responses. *Proc. Natl. Acad. Sci.* **107 :** 14621–14626

Jakobsson T, Treuter E, Gustafsson J-Å & Steffensen KR (2012) Liver X receptor biology and pharmacology: new pathways, challenges and opportunities. *Trends Pharmacol. Sci.* **33:** 394–404

Jung UJ, Millman PN, Tall AR & Deckelbaum RJ (2011) n−3 Fatty acids ameliorate hepatic steatosis and dysfunction after LXR agonist ingestion in mice. *Biochim. Biophys. Acta - Mol. Cell Biol. Lipids* **1811:** 491–497

Katz A, Nambi SS, Mather K, Baron AD, Follmann DA, Sullivan G & Quon MJ (2000) Quantitative Insulin Sensitivity Check Index: A Simple, Accurate Method for Assessing Insulin Sensitivity In Humans. *J. Clin. Endocrinol. Metab.* **85:** 2402–2410

Katz A, Udata C, Ott E, Hickey L, Burczynski ME, Burghart P, Vesterqvist O & Meng X (2009) Safety, Pharmacokinetics, and Pharmacodynamics of Single Doses of LXR-623, a Novel Liver X-Receptor Agonist, in Healthy Participants. *J. Clin. Pharmacol.* **49:** 643–649

Kopp E & Ghosh S (1994) Inhibition of NF-kappa B by sodium salicylate and aspirin. *Sci.* **265 :** 956–959

Kuhn M, Campillos M, Letunic I, Jensen LJ & Bork P (2010) A side effect resource to capture phenotypic effects of drugs. *Mol. Syst. Biol.* **6:**

Lamb J, Crawford ED, Peck D, Modell JW, Blat IC, Wrobel MJ, Lerner J, Brunet J-P, Subramanian A, Ross KN, Reich M, Hieronymus H, Wei G, Armstrong SA, Haggarty SJ, Clemons PA, Wei R, Carr SA, Lander ES & Golub TR (2006) The Connectivity Map: Using Gene-Expression Signatures to Connect Small Molecules, Genes, and Disease. *Sci.* **313 :** 1929–1935

Larsen TM, Toubro S & Astrup A (2003) PPARgamma agonists in the treatment of type II diabetes: is increased fatness commensurate with long-term efficacy? *Int. J. Obes.* **27:** 147–161

Law V, Knox C, Djoumbou Y, Jewison T, Guo AC, Liu Y, Maciejewski A, Arndt D, Wilson M, Neveu V, Tang A, Gabriel G, Ly C, Adamjee S, Dame ZT, Han B, Zhou Y & Wishart DS (2013) DrugBank 4.0: shedding new light on drug metabolism. *Nucleic Acids Res.*

Lund EG, Menke JG & Sparrow CP (2003) Liver X Receptor Agonists as Potential Therapeutic Agents for Dyslipidemia and Atherosclerosis. *Arterioscler. Thromb. Vasc. Biol.* **23 :** 1169–1177

Matthews DR, Hosker JP, Rudenski AS, Naylor BA, Treacher DF & Turner RC (1985) Homeostasis model assessment: insulin resistance and β-cell function from fasting plasma glucose and insulin concentrations in man. *Diabetologia* **28:** 412–419

Millatt LJ, Bocher V, Fruchart J-C & Staels B (2003) Liver X receptors and the control of cholesterol homeostasis: potential therapeutic targets for the treatment of atherosclerosis. *Biochim. Biophys. Acta - Mol. Cell Biol. Lipids* **1631:** 107–118

Mudaliar S & Henry RR (2012) The incretin hormones: from scientific discovery to practical therapeutics. *Diabetologia* **55:** 1865–1868

Nolte Kennedy MS (2012) Pancreatic Hormones & Antidiabetic Drugs. In *Basic & Clinical Pharmacology (12th ed.)*, Katzung BG Masters SB & Trevor AJ (eds) New York, NY: McGraw-Hill

Oie S, Matsuzaki K, Yokoyama W, Tokunaga S, Waku T, Han S-I, Iwasaki N, Mikogai A, Yasuzawa-Tanaka K, Kishimoto H, Hiyoshi H, Nakajima Y, Araki T, Kimura K, Yanagisawa J & Murayama A (2014) Hepatic rRNA Transcription Regulates High-Fat-Diet-Induced Obesity. *Cell Rep.* **7:** 807–820

Oosterveer MH, Grefhorst A, van Dijk TH, Havinga R, Staels B, Kuipers F, Groen AK & Reijngoud D-J (2009) Fenofibrate Simultaneously Induces Hepatic Fatty Acid Oxidation, Synthesis, and Elongation in Mice. *J. Biol. Chem.* **284 :** 34036–34044

Pacini C, Iorio F, Gonçalves E, Iskar M, Klabunde T, Bork P & Saez-Rodriguez J (2013) DvD: An R/Cytoscape pipeline for drug repurposing using public repositories of gene expression data. *Bioinformatics* **29:** 132–134

Poulsen L la C, Siersbæk M & Mandrup S (2012) PPARs: fatty acid sensors controlling metabolism. *Semin. Cell Dev. Biol.* **23:** 631–9

Praticò D & Dogné J-M (2005) Selective Cyclooxygenase-2 Inhibitors Development in Cardiovascular Medicine. *Circ.* **112 :** 1073–1079

Radonjic M, Wielinga PY, Wopereis S, Kelder T, Goelela VS, Verschuren L, Toet K, van Duyvenvoorde W, van der Werff van der Vat B, Stroeve JHM, Cnubben N, Kooistra T, van Ommen B & Kleemann R (2013) Differential Effects of Drug Interventions and Dietary Lifestyle in Developing Type 2 Diabetes and Complications: A Systems Biology Analysis in LDLr−/− Mice. *PLoS One* **8:** e56122

Reddy JK & Sambasiva Rao M (2006) Lipid Metabolism and Liver Inflammation. II. Fatty liver disease and fatty acid oxidation. **290:** G852–G858

Rena G, Pearson E & Sakamoto K (2013) Molecular mechanism of action of metformin: old or new insights? *Diabetologia* **56:** 1898–1906

Schultz JR, Tu H, Luk A, Repa JJ, Medina JC, Li L, Schwendner S, Wang S, Thoolen M, Mangelsdorf DJ, Lustig KD & Shan B (2000) Role of LXRs in control of lipogenesis. *Genes Dev.* **14 :** 2831–2838

Shaw RJ, Lamia KA, Vasquez D, Koo S-H, Bardeesy N, DePinho RA, Montminy M & Cantley LC (2005) The Kinase LKB1 Mediates Glucose Homeostasis in Liver and Therapeutic Effects of Metformin. *Sci.* **310 :** 1642–1646

Shoelson S (2002) JMM – Past and Present. Invited comment on W. Ebstein: On the therapy of diabetes mellitus, in particular on the application of sodium salicylate. *J. Mol. Med.* **80:** 618–619

Sirota M, Dudley JT, Kim J, Chiang AP, Morgan AA, Sweet-Cordero A, Sage J & Butte AJ (2011) Discovery and Preclinical Validation of Drug Indications Using Compendia of Public Gene Expression Data. *Sci. Transl. Med.* **3 :** 96ra77–96ra77

Soccio RE, Chen ER & Lazar MA (2014) Thiazolidinediones and the Promise of Insulin Sensitization in Type 2 Diabetes. *Cell Metab.* **20:** 573–591

Staels B, Dallongeville J, Auwerx J, Schoonjans K, Leitersdorf E & Fruchart J-C (1998) Mechanism of Action of Fibrates on Lipid and Lipoprotein Metabolism. *Circ.* **98 :** 2088–2093

Steffensen KR & Gustafsson J-Å (2004) Putative Metabolic Effects of the Liver X Receptor (LXR). *Diabetes* **53 :** S36–S42

Steinberg GR, Dandapani M & Hardie DG (2013) AMPK: mediating the metabolic effects of salicylate-based drugs? *Trends Endocrinol. Metab.* **24:** 481–487

Subramanian A, Tamayo P, Mootha VK, Mukherjee S, Ebert BL, Gillette MA, Paulovich A, Pomeroy SL, Golub TR, Lander ES & Mesirov JP (2005) Gene set enrichment analysis: A knowledge-based approach for interpreting genome-wide expression profiles. *Proc. Natl. Acad. Sci. United States Am.* **102 :** 15545–15550

Sukardi H, Zhang X, Lui EY, Ung CY, Mathavan S, Gong Z & Lam SH (2012) Liver X receptor agonist T0901317 induced liver perturbation in zebrafish: Histological, gene set enrichment and expression analyses. *Biochim. Biophys. Acta - Gen. Subj.* **1820:** 33–43

Tousoulis D, Psarros C, Demosthenous M, Patel R, Antoniades C & Stefanadis C (2014) Innate and Adaptive Inflammation as a Therapeutic Target in Vascular DiseaseThe Emerging Role of Statins. *J. Am. Coll. Cardiol.* **63:** 2491–2502

Ulven SM, Dalen KT, Gustafsson J-Å & Nebb HI (2005) LXR is crucial in lipid metabolism. *Prostaglandins, Leukot. Essent. Fat. Acids* **73:** 59–63

Vasudevan AR & Balasubramanyam A (2004) Thiazolidinediones: A Review of Their Mechanisms of Insulin Sensitization, Therapeutic Potential, Clinical Efficacy, and Tolerability. *Diabetes Technol. Ther.* **6:** 850–863

Waget A, Cabou C, Masseboeuf M, Cattan P, Armanet M, Karaca M, Castel J, Garret C, Payros G, Maida A, Sulpice T, Holst JJ, Drucker DJ, Magnan C & Burcelin R (2011) Physiological and Pharmacological

Mechanisms through which the DPP-4 Inhibitor Sitagliptin Regulates Glycemia in Mice. *Endocrinology* **152:** 3018–3029

Wellen KE & Hotamisligil GS (2003) Obesity-induced inflammatory changes in adipose tissue. *J. Clin. Invest.* **112:** 1785–1788

Williamson RT (1901) On the Treatment of Glycosuria and Diabetes Mellitus with Sodium Salicylate. *BMJ* **1:** 760–762

Zanotti I, Potì F, Pedrelli M, Favari E, Moleri E, Franceschini G, Calabresi L & Bernini F (2008) The LXR agonist T0901317 promotes the reverse cholesterol transport from macrophages by increasing plasma efflux potential. *J. Lipid Res.* **49 :** 954–960

Zhao C & Dahlman-Wright K (2010) Liver X receptor in cholesterol metabolism. *J. Endocrinol.* **204 :** 233–240
