# Supplementary material for: Drugs that reverse disease transcriptomic signatures are more effective in a mouse model of dyslipidemia
Source: Mol Syst Biol. 2015 Mar 3;11(3):0791. doi: 10.15252/msb.20145486 (PMC4380926; doi:10.15252/msb.20145486)
Supplement: Supplementary file 2 [file msb0011-0791-sd2.zip › msb145486-sup-0007-Suppl-datasetS1/LDLR_paper_code/reference_manual.pdf]

The following reference manual to accompanies the code submitted with the paper "Drugs that reverse disease transcriptomic signatures are more effective in a mouse model of dyslipidemia" by A. Wagner, N. Cohen, T. Kelder, U. Amit, E. Liebman, D. M. Steinberg, M. Radonjic & E. Ruppin.

Included Functions:

CloseAllPlots.m **2**

DoMainAnalysis.m **2**

dsxy2figxy.m **2**

EvaluateExpressionModule.m **3**

FisherFeatureSelectionScore.m **3**

GetColorForIntervention_LDLR.m **3**

GetLinearRegressionRSquared.m **4**

GetTissueData.m **4**

GetTranscriptomeDistance.m **5**

PlotPCA.m **5**

ReadLDLR_NormalizedExpression.m **6**

ReadLDLR_PhysiologicalData.m **6**

ScatterInterventions.m **6**

CloseAllPlots.m

```
function CloseAllPlots
```

Closes all the currently open figures.

DoMainAnalysis.m

This is the main analysis script, runs the following steps (the different steps are highlighted in the documentation of the code itself):

- It first reads data through the GetTissueData function. Two data types are read: the transcriptomic and the physiological data by the functions ReadLDLR_NormalizedExpression and ReadLDLR_PhysiologicalData, respectively.
- The user may select whether to average probe data for probes that are associated with the same gene symbol. In the current study we did not do that to accommodate cases where probes associated with the same gene carry different information, and to maintain a completely data-driven approach. We verified, however, that averaging gene probes does not significantly alter results.
- Dimensionality reduction then follows in three steps:
    - Keeping only the genes that are differentially expressed between the untreated disease and healthy groups (default: keep top 200 differentially expressed genes).
    - Compute Fisher scores for the differentially expressed genes that weight them by their ability to distinguish between the untreated disease and healthy groups.
    - PCA-transform, weighted by the Fisher scores. A 3-dimensional plot of the top 3 PCs is produced.
- The script now runs an analysis that tests whether the treated disease animals are closer to the healthy animals than are the untreated disease animals. P-values for each treated group are computed. This analysis is the one presented in Supplementary Results 4.
- TDIs and PDIs are computed from the transcriptomic and physiological data, respectively, and are correlated one against the other.

dsxy2figxy.m

```
function varargout = dsxy2figxy(varargin)
```

Transforms point or position from data space coordinates into normalized figure coordinates for the purpose of producing figures. This is a Matlab core function that is usually not include in the distributions. See in the function itself for further details.

EvaluateExpressionModule.m

```
function [cRank, pRank, dataForSpearmanPlot] = EvaluateExpressionModule(exp,
physiologicalData, markerString, varargin)
```

Computes the correlation (either Spearman or Pearson) between the TDIs and the PDI of a given marker and plots a corresponding scatter plot. The marker's values and a printable string with its name are given in the physiologicalData, markerString arguments, respectively. exp is the expression matrix (rows: features, columns: animals).

Optional arguments are:

- ExcludeAnimals: a binary vector or a list of indices for animals to be excluded from the analysis.
- ShowPearsonPlot/ShowSpearmanPlot: whether to plot a scatter plot showing the Pearson or Spearman correlations, respectively. In the latter case, the plot shows the ranks of each data point in the x- and y-axes, in accordance with the definition of the Spearman correlation coefficient.

FisherFeatureSelectionScore.m

```
function score = FisherFeatureSelectionScore(X,labels)
```

Computes the generalized Fisher score and supports separation into an arbitrary number of classes, although in the current work only two classes are used (healthy and untreated disease). See the paper "Generalized Fisher Score for Feature Selection" by Gu and Han (http://arxiv.org/abs/1202.3725) for details. X holds the data (rows of X - features, columns of X - observations) and the labels (which are assumed to be numbers) denote for which observation to which class it belongs. It is therefore assumed the the number of labels equals the number of columns in X.

GetColorForIntervention_LDLR.m

```
function color = GetColorForIntervention_LDLR(interventionString,
HEALTHY_DESCRIPTION, PRE_DESCRIPTION)
```

Makes sure that all the experimental groups are consistently drawn using the same colors every time. The function accepts a the name of one of the intervention groups (out of a predefined set), as well as the names of the healthy and untreated groups (see in the data loading procedures for explanation of these variables) and returns the group's color.

GetLinearRegressionRSquared.m

```
function [rsq, adjusted_rsq, beta] = GetLinearRegressionRSquared(x, y)
```

Returns r squared, adjusted r squared and beta, which is a vector of regression coefficients, for a linear regression between the dependent variable y and the explanatory variable x.

GetTissueData.m

```
function [expressionMatrix, animalNumber, interventionString, probeList,
eBayesTable, physiologicalData, markerString, HEALTHY_DESCRIPTION,
PRE_DESCRIPTION, GetColorForIntervention_FunctionHandle] =
GetTissueData(TISSUE_TO_USE, RELOAD_DATA)
```

Loads the data for the analysis. It accepts two input arguments: a) TISSUE_TO_USE which is assumed to be in the format MAJOR_MINOR (i.e., an underscore separating them). Major is the dataset to use (currently supported only LDLR - the dataset analyzed in the paper) and minor identifies a subset, which in our study represents one of the two tissues. b) RELOAD_DATA - boolean added for convenience. When true, data is reloaded from scratch and saved in a .mat file. When false this .mat file is read to save time.

The function returns:

a. expressionMatrix = gene expression. Rows are probes, columns are
b. animalNumber = ID number for each animal participating in the study, that allows tying its gene expression and physiological data.
c. interventionString = a vector of strings whose length equals the number of columns in expressionMatrix and denotes the experimental group to which each animal belongs.
d. probeList = a table containing metadata on the rows of of the expressionMatrix (e.g., probe IDs, the genes to which they correspond etc.).
e. eBayesTable = as produced by R bioconductor's limma package.
f. physiologicalData = a numeric table that contains physiological data on the study animals. Its rows are animals, and its columns are physiological markers (it is assumed that the number of rows equals the number of *columns* in expressionMatrix).
g. markerString = a string representation of the name of each of the physiolocal markers (this is a vector of strings assumed to be in the same length as the number of columns in physiological).
h. HEALTHY_DESCRIPTION = the name of one of the experimental groups, which represents the healthy state (in our study: the LFD group). i) PRE_DESCRIPTION = the name of one of the experimental groups, which represents the untreated disease state (in our study: the untreated 16 weeeks HFD group).

i. GetColorForIntervention_FunctionHandle = a function handle for a function that gets the name of one of the experimental groups and returns a color. This is used to make sure that each experimental group is drawn with a consistent color. See the function GetColorForIntervention_LDLR.m for its prototype.

GetTranscriptomeDistance.m

```
function [transcriptomeDistance, healthyTranscriptomeRepresentative,
preTranscriptomeRepresentative, desiredDirection, animalRejectionVectors] =
GetTranscriptomeDistance(exp, varargin)
```

Computes TDIs and returns them in transcriptomeDistance. Also returns:

a. healthyTranscriptomeRepresentative - the point from which distances to the healthy group are computed. By default this is the centroid of the healthy group.
b. preTranscriptomeRepresentative - same, for the untreated group.
c. desiredDirection, animalRejectionVectors – direction of the desired change (i.e., from untreated representative to healthy representative) and the rejection vector of each animal on it.

Optional arguments are:

- ExcludeAnimals: a binary vector or a list of indices for animals to be excluded from the analysis.
- DISTANCE_METHOD can get one of 3 values:
    a. L_NORM = L_p norm, by default p = 2 (i.e., Euclidean distances). P is controlled by the constant NORM_P.
    b. PROJECTION = the norm of the projection of the animal's point onto the vector that goes in the desired direction of change (i.e., from the untreated presentative to healthy representative).
    c. REJECTION = the same, substituting vector rejection for vector projection (this option is used for assessing side-effects)

PlotPCA.m

```
function combined_fig_h = PlotPCA(expPCA, outside_params)
```

Plots the gene expression space, coloring each of the experimental groups in its regular color. expPCA is the PCA-transformed data, outside_params can be used to pass additional info to this function. Currently, it used to pass the explained variance by each of the PCs which is printed as part of the axes labels.

ReadLDLR_NormalizedExpression.m

```
function [expressionMatrix, mouseNumber, probeList, eBayesTable] =
ReadLDLR_NormalizedExpression(tissueID)
```

Reads normalized gene expression, as produced by R bioconductor's limma package. The returned arguments from this function are transferred to the function GetTissueData.m. Refer to its documentation for their meaning

ReadLDLR_PhysiologicalData.m

```
function [physiologicalData, markerString, interventionString] =
ReadLDLR_PhysiologicalData(mouseNumber)
```

Reads the physiological data associated with the animals and return it ordered by the same order of animals as in the animal ID vector mouseNumber. The returned arguments from this function are transferred to the function GetTissueData.m.

The function returns 3 variables that describe the physiological data:

a. physiologicalData = a numeric matrix whose rows correspond to animals, and whose columns correspond to physiological markers.
b. markerString = a string representation of the name of each of the physiological markers. This is assumed to be a vector of strings of the same length as the number of columns in physiologicalData
c. interventionString = a string representation of the experimental group to which each animal belongs. This is assumed to be a vector of the same length of as the number of rows in physiologicalData.

NOTE: the input argument mouseNumber is a vector of unique IDs associated with the studied animals. Both the rows of physiologicalData and interventionString are expected to be returned ordered in the order dictated by the mouseNumber input argument

ScatterInterventions.m

```
function [fig, rsq, beta] = ScatterInterventions(GetColorForIntervention, X,
Y, interventionString, varargin)
```

A 2-dimensional scatter plotthat makes sure that the different experimental groups are consistently colored in the same colors. X and Y are assumed to be vectors of the same length, each dot i in the scatter plot will correspond to (X(i), Y(i)). fig is a handle to the resulting figure, rsq and beta are computed by the GetLinearRegressionRSquared function.
