# Supplementary material for: Drugs that reverse disease transcriptomic signatures are more effective in a mouse model of dyslipidemia
Source: Mol Syst Biol. 2015 Mar 3;11(3):0791. doi: 10.15252/msb.20145486 (PMC4380926; doi:10.15252/msb.20145486)
Supplement: Supplementary file 3 [file msb0011-0791-sd3.pdf]

Drugs that reverse disease transcriptomic signatures are more effective in a mouse model of dyslipidemia

Allon Wagner, Noa Cohen, Thomas Kelder, Uri Amit, Elad Liebman, David M. Steinberg, Marijana Radonjic and Eytan Ruppin

*Corresponding author: Eytan Ruppin, Tel-Aviv University -**

Review timeline:

Submission date:	13 June 2014
Editorial Decision:	18 July 2014
Revision received:	20 October 2014
Editorial Decision:	01 December 2014
Revision received:	18 January 2015
Accepted:	23 January 2015

Editor: Thomas Lemberger

Transaction Report:

(Note: With the exception of the correction of typographical or spelling errors that could be a source of ambiguity, letters and reports are not edited. The original formatting of letters and referee reports may not be reflected in this compilation.)

1st Editorial Decision 18 July 2014

Thank you again for submitting your work to Molecular Systems Biology. We have now heard back from three referees who agreed to evaluate your manuscript. As you will see from the reports below, the referees find the topic of your study of potential interest. They raise, however, several issues on your work, which should be convincingly addressed in a revision of the study.

The most important points are the following:
- the quantification of treatment effects and side-effects should be more rigorous, in particular with regard to the interpretation of the data shown in Figure 2a&b (review #1 and more specifically, reviewer #2)
- A comparison between your PCA-based method with the results obtained with a pathway-based method (reviewer #2, reviewer #3 refers specifically to GSEA) should be performed.
- the potential limitation of the disease model should be clearly discussed (reviewer #3).
- some of the choices made, eg with regard to the selection of drugs or the markers for side-effects, should be justified or complemented with more systematical approaches.

If you feel you can satisfactorily deal with these points and those listed by the referees, you may wish to submit a revised version of your manuscript. Please attach a covering letter giving details of the way in which you have handled each of the points raised by the referees. A revised manuscript will be once again subject to review and you probably understand that we can give you no guarantee at this stage that the eventual outcome will be favorable.

© European Molecular Biology Organization

REFEREE REPORTS

Reviewer #2:

In this work, Wagner and colleagues test an hypothesis widely (and successfully) used for computational drug repositioning: that alterations induced by drugs at the molecular level (as described by transcriptional data) are correlated with their physiological consequence. As authors state, despite its broad use to study and repurpose drugs, this hypothesis has not been validated in a systematic way. Perhaps this is due to the fact that this sounds so natural that it does not require validation. In any case, given the broad and growing use of this rationale, this study it is a very welcomed contribution to the field, that provides a solid basis for previous and future analyses based on this idea.

Wagner et al. used a mouse model under different dietary conditions, in combination with exposure to different drugs (based on a recently published study), to test the hypothesis. They could find that the rationale above largely holds, and treatments that revert expression patterns are associated with restoration of physiological readouts. In addition they could shed light on the related idea that side-effects are due to additional 'non-restorative' changes in gene expression due to drug treatment, using an elegant formalisation to quantify side effects.

Below we outline some questions and concerns that we consider authors should address:

MAJOR POINTS
- It is a bit puzzling that for the majority of the drugs the corresponding expression profiles do not tend to cluster together. For example, in figure 2 points with same color do not seem to tend to be close to each other? apparently this happens only for a minority of drugs, and are those that do not exert an effective treatment.
Can authors quantify this similarity? And what would be their explanation for this phenomenon? Does it have a biological reason, or is it perhaps a result of the type of analysis made? For example, authors may want to look beyond the two first principal components that are used in their analysis, since these may not be enough to cover the observed variability? Or even they may want to use more sophisticated scaling/dimensionality reductions techniques, such as MDS or t-SNE? Authors state in their methods "further dimensionality reduction was performed to produce the results presented in this study; we verified that further dimensionality reduction (by selecting only the top principal components) did not significantly alter the results."

- Authors state that "most dots lie close to the dashed arrow that leads from the HFD mean (circled in red) to the LFD mean (circled in blue). Hence, the treatments tend to alter the gene expression of the treated HFD groups in a way that brings it closer to the gene expression of the LFD group" . To us, there is no clear support for this statement from the data.
First of all, the proximity to the 'trajectory' leading from the the 'disease' to the healthy state is not quantified and its statistical significance not assessed. This should be performed.
Secondly, we are not convinced of the justification for this to be a 'trajectory'. Proximity to this line does not necessarily imply that treating with the drugs corresponding to circles that are close to the line (linking the disease to the healthy state) on the first two PC of the resulting expression dataset, actually 'brings the samples from the disease to the normal state'. A trajectory could be obtained by for example a time course analysis of gene expression following drug treatment, in which one could see how the samples tend to 'move' from the disease state to the healthy one across different consecutive observation time points (and this trajectory would not be necessarily a straight line).

- How the ten drugs were chosen? a very brief description of each compound should be provided.

- Can the mode of action of the drug be connected to the results obtained, e.g. to the similarities in gene-expression and physiological space? Even more, authors could perform a further analysis, as they state in the discussion "...to incorporate into the gene expression spaces known pathways and gene- modules ...".
While we appreciate that there is a limit to what can put into a single manuscript, some (even if simple) functional /pathway-based/mechanistic analysis of the data in relation to the drug mode of

action would enhance the results and possibly help to address the two points mentioned above.

- Authors should provide the code used to perform the analysis that lead to the results and figures of the paper.

MINOR POINTS

- End of page 3: "This model is particularly apt to study the questions at hand since diagnosis and clinical risk- assessment in the case of the metabolic syndrome and related disorders depend almost exclusively on physiological markers". Does this make the results presented in this manuscript very 'disease-specific'? We feel this should be further discussed. What is the expected the general applicability of this analysis to more complex disease where the phenotype is not very easily "quantifiable"?

- LDLR should be defined at its first occurrence

- The authors describe their reduction as 'Euclidean embedding' ; it seems to be a simple PCA?. Authors should provide a clarification of the use of terms.

DISCRETIONARY POINTS
Finally, some very minor suggestions about writing:
- Authors claim, their work represents "a sound theoretical basis to in silico methods that rely on omic metrics for drug repositioning (repurposing) and drug discovery". We would not use 'theoretical' in this context - in our opinion, the key value of this work is that it provides an experimental support for that, certainly with the corresponding computational analysis associated, but theoretical suggest at least to us a novel theorem or something alike. We would suggest to simply remove 'theoretical' from the sentence.

- In the abstract the authors claim that "patients' diagnosis and response to treatment are often judged through measurements of disease-relevant physiological markers, mostly in blood and urine". This sounds a bit too specific to the disease they are interested in.

- The following points, stating the 2 key hypothesis testes in the work, seem to be expressed in a rather verbose manner and we feel could be simplified: " to what extent is the restoration of physiological indices back to their baseline levels following extant treatments accompanied by a reversal of the disease phenotypes at the molecular level? And (2) when treatments lead to molecular-level alterations that are incongruent with reversal of disease phenotypes, are parallel maladaptive responses observed at the physiological level? "

- The use of HFD to indicate both the HFD-untreated group and the group of mouses sacrificed at 9 weeks is quite confusing. Maybe the first group could be indicated with HFDu

Reviewer #3:

The present manuscript is a commendable re-analysis of published data, showing that there is a correlation between deviations in gene expression and physiological measurements, and that side effects of drug treatment may be correlated with aberrant gene expression states of the studied tissues. The authors developed a transcriptome deviation index (TDI) that aims to capture difference between healthy and untreated mice, aiming at reducing the high-dimensional transcriptome space to just one dimension.

As detailed below, I am not entirely convinced by the key conclusions. If these concerns can be addressed, the work would be of interest for a wide audience interested in the effects of drugs. The main innovation is the TDI, which is demonstrated to be correlated with physiological measures.

At first, it was surprising to me that there is such a high correlation between the gene expression and the physiological measures. However, this is probably explained from the fact that the studied physiological markers are quite closely related to adipose and liver function and therefore do not

© European Molecular Biology Organization

capture off-target effects caused within other tissues. However, it is clear that such side effects are a main concern in human drugs.

My main disagreement is with the statements "most dots lie close to the dashed arrow" (p. 5) and "In this paper, we examined whether effective drug treatments, which restore various organism-level physiological indices to their norm, also serve to reverse molecular-level, disease-induced gene expression deviations from the norm." (p. 9): In 2a, most dots are close to the untreated condition and in 2b the dots are close to the line for ~half of the drugs. As seen in Suppl. Fig. 2, only dietary intervention really restores the physiology to the norm and T0901317 comes closest of all the drugs. Interestingly, it also causes side effects in the liver. Could this be an effect of dosage, i.e. that the other drugs are under-dosed to have a better treatment effect? The authors should better quantify treatment success to motivate the two statements.

It's also problematic that the authors measure treatment effect and side effects using the same scale. Put differently, there should be physiological measures that don't change between healthy and untreated mice, but upon drug treatment. These would be true side effects. However, as I understand the weighting scheme, these would have little impact on the GPDI. So perhaps a different index (focussing on stability rather than deviation) should be employed to judge the strength of side effects (let's call this SEPDI). The authors propose that the gene expression deviation is made up of two components: restorative and non-restorative alterations. To show that these components are valid, they should be plotted against GPDI and SEPDI. The restorative alterations should correlate stronger with the GPDI, while the non-restorative deviations should correlate stronger with the SEPDI.

Why does T0901317 have such a high magnitude of non-restorative changes in the adipose tissue? Judging from Fig. 2b it seems to be quite close to the dashed line.

Concerning the presentation: the many colors are quite hard to distinguish. I would suggest to also use different point shapes for similar colors. In Fig 2, it should be "principal" instead of "principle".

Reviewer #4:

"Drugs that successfully reverse disease transcriptomic signatures are more effective and have lesser side effects: lessons from a mouse model of dyslipidemia."

This is an interesting manuscript that sheds light on some issues surrounding drug repurposing by inverse transcriptomic profiles. However, there are some parts of the manuscript that need clarification and some parts of the methods that need to be extended. Moreover, it is based on one disease only which makes it not clear how generalizable the principle is.

Major comments:

1. How were the drug treatments selected?
Neither this manuscript nor the PLoS ONE paper that published most of the data on which this manuscript is based mentions selection of drug candidates. It is not obvious to me that the drugs that revert the gene expression profile in the animal, are the same as one would find in vitro. For a true insight into drug repurposing by inverse profiling, the authors should use a method such as DvD (Pacini et al, bioinformatics 2013; publicly available as R/Cytoscape module) to predict which are the best candidate drugs and then test if indeed these correspond to the drugs that revert gene expression in the disease model.

2. I have some doubts about the disease model. Judging from figures 2 and 3, salicylic acid (aspirin) is consistently one of the best treatment options, whereas it has been reported to be only moderately effective against dyslipidemia and only in certain patients. Moreover, it is associated with risk of bleeding which is not assessed as side-effect by the authors. The authors should discuss the best treatment options according to this study and why they are the most effective in this case. Eg. could it be the mice are on high-fat diet for a period that is too short?
3. Regarding side-effects. Why are 26 physiological markers taken to assess side-effects (page 8)? Why not take the real side-effects as indicated on drug labels and measure those? The choice seems

arbitrary here and also ignores some severe side-effects as mentioned above.
4. How does the method of transforming gene expression profiles with PCA compare to Gene Set Enrichment Analysis that has been used in most previous manuscripts (eg: Dudley et al)? In GSEA the differentially expressed genes from the treatment are compared to the gene expression profile of the disease state to find anti-correlation and result in one clear score. Is there a reason to not use an established method like GSEA? Also, can the choice of 200 most differentially expressed genes be justified? Is the method robust to taking a different number of genes?
5. What is the variation between mice? It would seem the variation is significant in Figure 2 b. Based on differences in gene-expression profiles, would different mice benefit from different drug-treatments? Can this be predicted from the data?
6. In order to translate the results into treatment practice, would we now need to take samples from patients for eg liver gene expression profiling to determine the optimal treatment strategy?

Minor comments:
1. The authors use 'lesser side-effects' in several places. I am sure they mean 'less side-effects' as in Figure 2 "the number of side-effects associated with treatment" is reported. As it is written now, they have less important side-effects rather than just less.

1st Revision - authors' response 20 October 2014

Reviewer #2:

In this work, Wagner and colleagues test an hypothesis widely (and successfully) used for computational drug repositioning: that alterations induced by drugs at the molecular level (as described by transcriptional data) are correlated with their physiological consequence. As authors state, despite its broad use to study and repurpose drugs, this hypothesis has not been validated in a systematic way. Perhaps this is due to the fact that this sounds so natural that it does not require validation. In any case, given the broad and growing use of this rationale, this study it is a very welcomed contribution to the field, that provides a solid basis for previous and future analyses based on this idea.
Wagner et al. used a mouse model under different dietary conditions, in combination with exposure to different drugs (based on a recently published study), to test the hypothesis. They could find that the rationale above largely holds, and treatments that revert expression patterns are associated with restoration of physiological readouts. In addition they could shed light on the related idea that side-effects are due to additional 'non-restorative' changes in gene expression due to drug treatment, using an elegant formalisation to quantify side effects.

· We thank the reviewers for this positive appreciation.

Below we outline some questions and concerns that we consider authors should address: MAJOR POINTS
- It is a bit puzzling that for the majority of the drugs the corresponding expression profiles do not tend to cluster together. For example, in figure 2 points with same color do not seem to tend to be close to each other? apparently this happens only for a minority of drugs, and are those that do not exert an effective treatment. Can authors quantify this similarity? And what would be their explanation for this phenomenon? Does it have a biological reason, or is it perhaps a result of the type of analysis made?

· We now point out the considerable intra-group variability in the manuscript (lines 172-173) and refer the reader to further hierarchical clustering analysis in the supplementary material (Supplementary Results 2, Supplementary Figures 12-13). The hierarchical clustering takes account of the full dimensionality of the gene expression space in contrast to the visual depiction of the PCA plots that show only the top two principal components. The new analysis corroborates the observations that are apparent from the PCA plots, including the considerable intra-group variability that the reviewer pointed out. We discuss this observation (Supplementary Results 2), and conclude that in our opinion it reflects the different mechanisms by which the drugs act. The four drugs that conspicuously cluster away from other groups all target master transcription factors, and thus lead to considerable cascading effects. Other drugs work through subtler mechanisms, such as inhibition of metabolic enzymes, or through molecular mechanisms that are located in tissues not included in the

current study, such as the pancreas. A supplementary table (Supplementary Table 2) has now been added, and details the mechanism of action of each drug in the study.

For example, authors may want to look beyond the two first principal components that are used in their analysis, since these may not be enough to cover the observed variability? Or even they may want to use more sophisticated scaling/dimensionality reductions techniques, such as MDS or t-SNE? Authors state in their methods "further dimensionality reduction was performed to produce the results presented in this study; we verified that further dimensionality reduction (by selecting only the top principal components) did not significantly alter the results."

· The two first principal components are shown only for the sake of visualization. Distance computations, throughout the manuscript, take account of all the dimensions of the PCA space. This might indeed not have been clearly explained in the manuscript. We replaced the sentence that the reviewer cites into one which we hope is clearer (lines 413-416): "All distance computations in this study used all the dimensions of the PCA space; i.e., we did not use only the top principal components. We verified that using only the top principal components did not significantly alter the results". In addition, we added the following sentence to the definition of GPDI (lines 437-438): "[The physiological space was defined in an analogous manner to the gene expression space…] Again, all principal components, rather than only the top ones, were used to define distances in the PCA space".

· PCA was chosen for dimensionality reduction in order to simplify the methodology employed in this work as much as possible, and thus to facilitate interpretation of the results. In the particular case of this manuscript, since we use only the Euclidean metric, distances obtained through classic MDS are equivalent to those obtained in the PCA space (Gower, 1966). In the course of the research we also tried to employ the more sophisticated Large Margin Nearest Neighbor (LMNN) algorithm (Weinberger & Saul, 2009) to learn a better distance metric in a supervised manner, based on the differences between the HFD and LFD groups. We found, however, that it failed to add more information beyond that revealed by the PCA analysis, probably due to the high dimension and small number of samples.

- Authors state that "most dots lie close to the dashed arrow that leads from the HFD mean (circled in red) to the LFD mean (circled in blue). Hence, the treatments tend to alter the gene expression of the treated HFD groups in a way that brings it closer to the gene expression of the LFD group" . To us, there is no clear support for this statement from the data. First of all, the proximity to the 'trajectory' leading from the the 'disease' to the healthy state is not quantified and its statistical significance not assessed. This should be performed. Secondly, we are not convinced of the justification for this to be a 'trajectory'. Proximity to this line does not necessarily imply that treating with the drugs corresponding to circles that are close to the line (linking the disease to the healthy state) on the first two PC of the resulting expression dataset, actually 'brings the samples from the disease to the normal state'. A trajectory could be obtained by for example a time course analysis of gene expression following drug treatment, in which one could see how the samples tend to 'move' from the disease state to the healthy one across different consecutive observation time points (and this trajectory would not be necessarily a straight line).

· The same concern was raised by reviewer #3. We accept the criticism, and revised accordingly - the relevant paragraph, which previously mixed two observations concerning Figure 2a-b was completely revised, as follows:

· The first observation was that "the treatments tend to alter the gene expression of the treated HFD groups in a way that brings it closer to the gene expression of the LFD group". We now qualify this observation by writing (page 167-169): "Many of the treatments altered the gene expression of the treated HFD groups in a way that brought it closer to the gene expression of the LFD group (Supplementary Results 1, Supplementary Figure 1)". Supplementary Results 1 provides a quantitative analysis that established this result. It had appeared already in the original manuscript, but it may have been unclear that it was supposed to support the point made here.

· The second observation was that four of the drugs, two in the adipose tissue and two in the liver, exerted strong non-restorative effects while the other drugs had more moderate non-restorative effects in comparison. This issue is discussed at length later in the manuscript (in the section "Non-

restorative alterations to the gene expression are associated with drug side-effects", on page 7-9), and was therefore omitted from the revised paragraph. We accept the reviewer's comment on the use of the word "trajectory" in the current study, which has no longitudinal data, and refrain from using it in the revised manuscript.

- How the ten drugs were chosen? a very brief description of each compound should be provided.

· Drugs were chosen for this study owing to their relevance to the disease in question. Eight of the drugs are FDA-approved and commonly prescribed to treat obesity-associated pathologies in human patients. The other two are an experimental compound, known to have anti-atherogenic effects in the mouse model we study, and an anti-inflammatory drug, included because inflammation is tightly coupled with atherogenesis. We thank the reviewer for noting the importance of addressing drug selection in the manuscript. To this end, we added lines 117-128 to the main text (page 4), Table 1, and Supplementary Table 2.

- Can the mode of action of the drug be connected to the results obtained, e.g. to the similarities in gene-expression and physiological space? Even more, authors could perform a further analysis, as they state in the discussion "...to incorporate into the gene expression spaces known pathways and gene- modules ...". While we appreciate that there is a limit to what can put into a single manuscript, some (even if simple) functional /pathway-based/mechanistic analysis of the data in relation to the drug mode of action would enhance the results and possibly help to address the two points mentioned above.

· To address this comment, the revised manuscript includes a GSEA analysis (main text lines 178-185, Supplementary Results 3) that compares the differentially expressed pathways between the untreated HFD group and all the other experimental groups. We find that all the drugs (except one) that work on central pathways in either liver or adipose tissues indeed exert their expected effects; Fenofibrate upregulated peroxisome proliferator-activated receptors (PPAR) signaling in the liver; fenofibrate, atorvastatin and T0901317 modulated hepatic fatty acid metabolism. Pioglitazone and rosiglitazone activated PPAR signaling and genes associated with fatty acid metabolism in white adipose. The only exception was metformin, which did not alter any hepatic pathway in a statistically-significant way. As detailed in the supplementary material, we suggest that stems from lack of statistical power rather than insufficient dosage to exert a significant effect.

· In addition, in various places in the manuscript we raise observations that tie the drug mechanism of action to the alterations observed in the gene expression and physiological spaces:

i. Pioglitazone and rosiglitazone belong to the same family (thiazolidinedione, or TZDs) and their effects are indeed similar in all analysis conducted in the manuscript. Most importantly, see Supplementary Figure 13 (a new figure added in during the current revision) which demonstrates their close association in adipose tissue, where they modulate the same major pathway.

ii. Pioglitazone and rosiglitazone modulate a key transcription factor (TF) that is active in adipose tissue, where indeed they conspicuously alter gene expression patterns (Supplementary Figure 10). Similarly, T0901317 and fenofibrate, which target master TFs in the liver, induce major similar changes in hepatic gene expression.

iii. T0901317 is shown to upregulate pro-inflammatory gene sets in the liver (e.g., KEGG_TYPE_I_DIABETES_MELLITUS), which is corroborated by the direct analysis in Supplementary Results 5. An inflammatory response probably mediates its adverse physiological side-effects in the liver (Figure 6c).

- Authors should provide the code used to perform the analysis that lead to the results and figures of the paper.

· A link to the code was added to the Methods section of the main text (lines 456-459). It can now be downloaded from: http://www.cs.tau.ac.il/~allonwag/LDLR_paper/LDLR_paper_code.zip

MINOR POINTS - End of page 3: "This model is particularly apt to study the questions at hand since diagnosis and clinical risk- assessment in the case of the metabolic syndrome and related

disorders depend almost exclusively on physiological markers". Does this make the results presented in this manuscript very 'disease-specific'? We feel this should be further discussed. What is the expected the general applicability of this analysis to more complex disease where the phenotype is not very easily "quantifiable"?

· Many diseases are diagnosed and monitored through clinical chemistries of blood and urine, mostly because they can be safely and economically collected from patients in their local community health center. For example, assessing thyroid functions through measurements of thyroid hormones in the plasma is less prone to complications and more cost-effective than collecting a tissue sample from the thyroid gland. This rationale incentivizes the large body of research seeking blood and urine biomarkers that correlate with the early onset and progression of various diseases (see recently (Hye *et al*, 2014)). The metabolic syndrome is unique though in being a major health concern that is diagnosed, monitored, and treated based *almost only* on physiological markers (Huang, 2009), and hence was chosen as the center of the current manuscript. Nonetheless, we believe that the prevalence of clinical chemistries in all areas of medicine, combined with the active search for novel blood biomarkers that are associated with various diseases, implies that the conclusions of the current study might extend to additional pathologies beyond obesity-associated disorders. We state this in the discussion (lines 350-354) yet qualify this statement by noting that the current research is limited to only one disease model, and further work is required to test whether it holds in other animal disease models, and, most importantly, whether it translates from animal models to human subjects. - LDLR should be defined at its first occurrence

· Corrected, thank you. The revised sentence now reads (lines 98-101): “We analyze hepatic and white adipose gene expression, as well as 26 disease-relevant physiological markers, measured in a LDLR−/− mouse model of diet-induced dyslipidemia (Supplementary Table 1). Low-Density Lipoprotein Receptor (LDLR)-deficient mice are genetically predisposed to develop hypercholesterolemia and atherosclerotic lesions”.

- The authors describe their reduction as 'Euclidean embedding' ; it seems to be a simple PCA?. Authors should provide a clarification of the use of terms.

· Indeed, as stated above, we conduct a straightforward PCA analysis. The use of the word Euclidean in various places was meant to clarify to that this was the metric chosen for calculation of distances (e.g., versus a L1 norm). Overall, we strived to make the description of the manuscript’s methodology in the results section as simple as possible, in order to make the manuscript accessible to clinicians, but to define the methodology precisely in the methods section.

DISCRETIONARY POINTS Finally, some very minor suggestions about writing: - Authors claim, their work represents "a sound theoretical basis to in silico methods that rely on omic metrics for drug repositioning (repurposing) and drug discovery". We would not use 'theoretical' in this context - in our opinion, the key value of this work is that it provides an experimental support for that, certainly with the corresponding computational analysis associated, but theoretical suggest at least to us a novel theorem or something alike. We would suggest to simply remove 'theoretical' from the sentence.
We accepted the suggestion and removed the word “theoretical”.

- In the abstract the authors claim that "patients' diagnosis and response to treatment are often judged through measurements of disease-relevant physiological markers, mostly in blood and urine". This sounds a bit too specific to the disease they are interested in.
The use of biofluid markers for clinical assessment is common in many areas of medicine (see recently (Enroth *et al*, 2014)). However, the metabolic syndrome is indeed unique at the extent in which clinicians rely on blood and urine indices, and we revised the relevant sentence in the abstract to reflect this as follows (lines 28-31): “High-throughput omics have proven invaluable in studying human disease, and yet day-to-day clinical practice still relies on physiological, non-omic markers. For example, the metabolic syndrome is diagnosed and monitored by blood and urine indices such as blood cholesterol levels.”

- The following points, stating the 2 key hypothesis tested in the work, seem to be expressed in a rather verbose manner and we feel could be simplified: " to what extent is the restoration of physiological indices back to their baseline levels following extant treatments accompanied by a

reversal of the disease phenotypes at the molecular level? And (2) when treatments lead to molecular-level alterations that are incongruent with reversal of disease phenotypes, are parallel maladaptive responses observed at the physiological level? "
Thank you for pointing this out. We rephrased as follows (lines 87-91): “[we] study two fundamental and related questions: (1) do extant treatments that restore physiological indices back to their baseline levels also reverse disease phenotypes at the molecular level? And (2) when treatments lead to additional molecular-level alterations other than disease reversal, are parallel deleterious responses observed at the physiological level?”
- The use of HFD to indicate both the HFD-untreated group and the group of mouses sacrificed at 9 weeks is quite confusing. Maybe the first group could be indicated with HFDu

· It might have been unclear that the designation “HFD-untreated” was reserved in the manuscript and denoted only the untreated 16 weeks group throughout. Whenever the 9 weeks group is referred to it is designated “HFD-9wks”. We clarify this in the revised manuscript by rephrasing as follows (lines 140-142): “The untreated 16-weeks HFD group (henceforth designated “untreated HFD” for brevity) was considered representative of an elevated-risk to develop cardiovascular disease.”

Reviewer #3:

The present manuscript is a commendable re-analysis of published data, showing that there is a correlation between deviations in gene expression and physiological measurements, and that side effects of drug treatment may be correlated with aberrant gene expression states of the studied tissues. The authors developed a transcriptome deviation index (TDI) that aims to capture difference between healthy and untreated mice, aiming at reducing the high-dimensional transcriptome space to just one dimension. As detailed below, I am not entirely convinced by the key conclusions. If these concerns can be addressed, the work would be of interest for a wide audience interested in the effects of drugs. The main innovation is the TDI, which is demonstrated to be correlated with physiological measures. At first, it was surprising to me that there is such a high correlation between the gene expression and the physiological measures. However, this is probably explained from the fact that the studied physiological markers are quite closely related to adipose and liver function and therefore do not capture off-target effects caused within other tissues. However, it is clear that such side effects are a main concern in human drugs.

· While off-target effects are indeed a prime concern, we note that the focus of the current manuscript is the correlation between molecular-level (as captured in gene expression) and physiological-level phenotypes. It is reasonable to look for such correlations in physiological indices that are related to the tissues in which gene expression was measured. One of our main results supports this premise by showing that gene expression alterations in each of the two tissues studied here are closely correlated with physiological markers that pertain to the specific functions of the same tissue (Figure 4). We completely agree, however, that given the relevant data is would be of considerable interest to test our conclusions in other experimental settings and additional tissues, as elaborated in the discussion section (lines 350-354). This is particularly true for the analyses that pertain to side-effects, as the reviewer notes below; a discussion of this limitation was added to the revised manuscript (lines 355-362).

My main disagreement is with the statements "most dots lie close to the dashed arrow" (p. 5) and "In this paper, we examined whether effective drug treatments, which restore various organism-level physiological indices to their norm, also serve to reverse molecular-level, disease-induced gene expression deviations from the norm." (p. 9): In 2a, most dots are close to the untreated condition and in 2b the dots are close to the line for ~half of the drugs. As seen in Suppl. Fig. 2, only dietary intervention really restores the physiology to the norm and T0901317 comes closest of all the drugs. Interestingly, it also causes side effects in the liver. Could this be an effect of dosage, i.e. that the other drugs are under-dosed to have a better treatment effect? The authors should better quantify treatment success to motivate the two statements.

· The same concern was raised by reviewer #2. We accept this criticism and as a result completely revised the relevant paragraph, which previously mixed two observations concerning Figure 2a-b, as follows:

· The first observation was that “the treatments tend to alter the gene expression of the treated HFD groups in a way that brings it closer to the gene expression of the LFD group”. We now qualify this observation by writing (page 167-169): “Many of the treatments altered the gene expression of the treated HFD groups in a way that brought it closer to the gene expression of the LFD group (Supplementary Results 1, Supplementary Figure 1)”. Supplementary Results 1 provides a quantitative analysis that established this result. It had appeared already in the original manuscript, but it may have been unclear that it was supposed to support the point made here.

· The second observation was that four of the drugs, two in the adipose tissue and two in the liver, exerted strong non-restorative effects while the other drugs had more moderate non-restorative effects in comparison. This issue is discussed at length later in the manuscript (in the section “Non-restorative alterations to the gene expression are associated with drug side-effects”, on pages 7-9), and was therefore omitted from the revised paragraph.

· No intentional over- or under-dosing was employed; proper dosages to elicit clinical response were determined by consultation with previously published peer-reviewed studies and records maintained by TNO (Netherland’s Organization for Applied Scientific Research). The GSEA analysis that was added to the revised manuscript shows that all the drugs, apart from metformin, which were expected to modulate major adipose or liver pathway indeed exerted a statistically significant effect (now in lines 178-185, Supplementary Results 3, Supplementary Tables 3-4, all of which added in the course of the revision). Metformin, however, was given in a dose which is comparable to the one used in previous studies (250 mg/kg, 0.25% w/w), alleviated some of the clinical phenotypes of the disease (Radonjic *et al*, 2013), and significantly decreased the hepatic TDI compared with untreated HFD group (Supplementary Results 1). This suggests that the drugs were not under-dosed.

It's also problematic that the authors measure treatment effect and side effects using the same scale. Put differently, there should be physiological measures that don't change between healthy and untreated mice, but upon drug treatment. These would be true side effects. However, as I understand the weighting scheme, these would have little impact on the GPDI. So perhaps a different index (focusing on stability rather than deviation) should be employed to judge the strength of side effects (let's call this SEPDI). The authors propose that the gene expression deviation is made up of two components: restorative and non-restorative alterations. To show that these components are valid, they should be plotted against GPDI and SEPDI. The restorative alterations should correlate stronger with the GPDI, while the non-restorative deviations should correlate stronger with the SEPDI.

· We completely agree with the reviewer that side-effects should be sought in markers (either transcriptomic or physiological) that are unchanged between the LFD and the untreated-HFD group. The reviewer rightfully observes that this makes GPDIs ill-suited for this task. For this reason we did not use GPDIs to assess physiological side-effects, but rather employed a different computation whose aim was to detect drug-induced disturbances that are otherwise not present. This is not the SEPDI metric proposed by the reviewer, but a different method that is motivated by the same spirit and can be computed based on the datasets studied in this manuscript. Please allow us to elaborate:

· The gist of the reviewer’s observation is that true side-effects are manifested in markers that are disturbed in some treatment group, but have comparable values in the untreated-HFD and LFD groups. However, the physiological markers in data we analyzed were measured in the first place due to their relevance to the disease in question. Therefore, this set is already biased towards markers that are affected by the high-fat diet. For this reason, we adopt a more general notion of side-effects: side-effects are manifested in markers in which the condition of the treated animals is significantly worse (in the statistical sense) than then that of the untreated HFD animals. This notion is quantified rigorously as described in the main text (lines 281-295). This formalization allows us to discern side-effects within the set of physiological markers that have been measured. However, we fully agree with the reviewer’s observation that side-effects should be primarily sought in biomarkers that remain stable between the untreated disease and the healthy state. We now address this limitation of our study in the discussion section and suggest that future studies should benefit from inspecting also biomarkers that are generally unaffected by the disease itself, but vary considerably between treated and untreated subjects (lines 355-362).

Why does T0901317 have such a high magnitude of non-restorative changes in the adipose tissue?

© European Molecular Biology Organization

Judging from Fig. 2b it seems to be quite close to the dashed line.

· We thank the reviewer for this sharp observation. Please allow us to elaborate:

· Figure 2 shows only the first two principal components of the gene expression space, which is standard when visualizing PCA spaces. These axes capture a considerable amount of the variance in the dataset, but not all of it. The reviewer is correct in noting that in the case of T0901317, almost of all the non-restorative changes occur outside of the first top principal components shown in Figure 2, and this results in the seeming discrepancy between Figure 2b and Figure 6c (we double checked our calculations and verified that this was indeed the cause). To address this point, we now added Supplementary Figure 13, which is computed based on all the dimensions of the gene expression space, and shows that while the T0901317 group is closer to the LFD group than any other pharmacological intervention group, it is still spaced apart from the LFD group. This contrasts with the DLI group, which is intermixed with the LFD group.

Concerning the presentation: the many colors are quite hard to distinguish. I would suggest to also use different point shapes for similar colors. In Fig 2, it should be "principal" instead of "principle".

· Thank you for noting the error. It has now been corrected. In addition, Figure 2, Supplementary Figure 5 and Supplementary Figure 10 now use different marker shapes to aid distinguishing similar colors. We added an explicit comment on the order by which the experimental groups are listed to the legend of Figure 6b-c.

Reviewer #4:

"Drugs that successfully reverse disease transcriptomic signatures are more effective and have lesser side effects: lessons from a mouse model of dyslipidemia." This is an interesting manuscript that sheds light on some issues surrounding drug repurposing by inverse transcriptomic profiles. However, there are some parts of the manuscript that need clarification and some parts of the methods that need to be extended. Moreover, it is based on one disease only which makes it not clear how generalizable the principle is. Major comments: 1. How were the drug treatments selected? Neither this manuscript nor the PLoS ONE paper that published most of the data on which this manuscript is based mentions selection of drug candidates.

· We thank the reviewer for noting this omission on our part. We rectified it by adding lines 117-128 to the main text (page 4), Table 1, and Supplementary Table 2.

· Drugs were chosen for this study owing to their relevance to the disease in question. Eight of the drugs are FDA-approved and commonly prescribed to treat obesity-associated pathologies in human patients. The other two are an experimental compound, known to have anti-atherogenic effects in the mouse model we study, and an anti-inflammatory drug, included because inflammation is tightly coupled with atherogenesis.

It is not obvious to me that the drugs that revert the gene expression profile in the animal, are the same as one would find in vitro. For a true insight into drug repurposing by inverse profiling, the authors should use a method such as DvD (Pacini et al, bioinformatics 2013; publicly available as R/Cytoscape module) to predict which are the best candidate drugs and then test if indeed these correspond to the drugs that revert gene expression in the disease model.

· As noted above, drugs were selected to this study on the grounds of already being indicated to treat dyslipidemia and related disorders. Rather than finding novel candidates through anti-correlative methods such as DvD, we sought to learn whether extant drug treatments, which are already known to alleviate the disease, induce the anti-correlative pattern that DvD searches. If effective and approved drugs induce an anti-correlative pattern, then this supports the conceptual basis of computational anti-correlative methods. We show that this is indeed the case, subject to the limitations detailed in the discussion section.

· In light of the reviewer's comment, we have now added an analysis that compares the metric by which we quantify transcriptomic disease-reversal (TDI) to the GSEA-based score used by DvD and

other studies (Supplementary Results 4.1 and Supplementary Figure 3). We show that the two are strongly correlated, as expected. Therefore, the ranking that DvD would have assigned to the animals in this study according to the success of their respective treatments to reverse the disease transcriptomic patterns is similar to that assigned according to TDIs. We show that the latter correspond to favorable physiological effects (again, subject to the limitations that we acknowledge), and this lends further support to the framework of DvD and related methods, as discussed in the manuscript (Introduction lines 75-86, Discussion lines 320-329, Summary lines 380-383).

2. I have some doubts about the disease model. Judging from figures 2 and 3, salicylic acid (aspirin) is consistently one of the best treatment options, whereas it has been reported to be only moderately effective against dyslipidemia and only in certain patients.

· We thank the reviewer for this sharp observation. Please allow us to elaborate:

· Salicylate (the main active compound in aspirin, as the reviewer notes) is an anti-inflammatory compound; as inflammation plays a critical role in atherogenesis (Ross, 1999; Libby *et al*, 2002; Hansson & Hermansson, 2011; Lumeng & Saltiel, 2011; Weber & Noels, 2011; van Diepen *et al*, 2013), it is quite conceivable that it will have favorable outcomes in the LDLR-/- mouse model, which is used to study diet-induced dyslipidemia and the resulting atherosclerotic disease. And indeed, treatment with aspirin has already been found to have positive consequences in LDLR-deficient mice (Jaichander *et al*, 2008). The crux of the reviewer's comment is asking whether this observation reflects clinical reality, and could be reproduced in human patients. It has been reported that aspirin treatment reduced cardiovascular risk (Ridker *et al*, 1997), and consequently it is commonly prescribed to prevent atherosclerotic complications in human patients (Awtry & Loscalzo, 2000; Campbell *et al*, 2007; Furst *et al*, 2012; American Diabetes Association, 2013).

· Furthermore, dyslipidemia and atherosclerosis tend to co-occur with other risk factors such as insulin resistance and obesity; when occurring together these conditions are designated "the metabolic syndrome" (Huang, 2009). One notes in this respect that there are many reports of favorable outcomes of salicylate and close compounds in diabetic patients (Williamson, 1901; Gilgore, 1960; Gilgore & Rupp, 1962; Baron, 1982; Hundal *et al*, 2002; Shoelson, 2002; Gao *et al*, 2003; Shoelson *et al*, 2006; Goldfine *et al*, 2013). One further notes that in our data salicylate outcomes have high variance both at the physiological and at the transcriptomic levels (Figures 2, 3, 5) – the condition of some of the animals is vastly improved compared with the untreated HFD groups, whereas other animals are only marginally affected. Therefore, our data suggests that salicylate is highly effective only in a subgroup of the studied animals.

· To address the reviewer comment, the revised manuscript now explicitly notes in the main text that aspirin is a member of the salicylates family, and points to its relevance to the metabolic syndrome (page 4 lines 121-123, Table 1). Both these points are elaborated at greater length in Supplementary Table 2 that has been added to the revised manuscript.

Moreover, it is associated with risk of bleeding which is not assessed as side-effect by the authors.

· Concerning the analysis of side-effects, please see below.

The authors should discuss the best treatment options according to this study and why they are the most effective in this case.

· All the drugs studied here are already known to be effective in treatment of various facets of the metabolic syndrome, and there exists a vast body of literature on their effects and mechanisms of action in human and in animal models. Furthermore, drug efficacy in the data that we study here has already been discussed to some extent in (Radonjic *et al*, 2013). Our contribution focuses on a different question - assessing the way that favorable or adverse physiological outcomes are paralleled in the animals' gene expression. In this respect, we decompose the transcriptomic drug effects into two components, restorative and non-restorative effects. We show that the former are associated with favorable outcomes, while the latter are associated with adverse side-effects. Rather than selecting the best treatment option among the ten drugs, these quantitative criteria allow a more complex view that measures both the positive and negative outcomes of each drug at the animal,

rather than the treatment group level. One notable conclusion, already reached by (Radonjic *et al*, 2013) through other means, is that the dietary lifestyle intervention strikingly outperformed all the pharmacological interventions in its ability to reverse the disease phenotypes without introducing non-restorative effects. These points are addressed in the discussion section, lines 332-349.

Eg. could it be the mice are on high-fat diet for a period that is too short?

· A period of 9 weeks or shorter of high-fat diet feeding has been used in previous studies as the period necessary to establish dyslipidemic phenotypes before commencing pharmacological treatment (for example, (Levin *et al*, 2005; Kappus *et al*, 2014)). In addition, in our study one group of mice was sacrificed after 9 weeks of high-fat diet (Figure 1a), and allowed us to verify that dyslipidemic phenotypes had indeed been established by that time.

3. Regarding side-effects. Why are 26 physiological markers taken to assess side-effects (page 8)? Why not take the real side-effects as indicated on drug labels and measure those? The choice seems arbitrary here and also ignores some severe side-effects as mentioned above.

· This point might have been ill-explained in the original manuscript. The 26 physiological markers are the entire corpus of physiological indices measured in the study animals, so there was no active selection of markers on our part. This set was used to assess both the favorable and the adverse physiological effects of the different intervention regimens. While we are aware of the limitations of this approach, we believe that it is warranted here; the data we study is unique in having both gene expression data and physiological indices measured *in the same animals*. This allows us to study the relationship of adverse physiological side-effects and trancscriptomic phenotypes based on data obtained from the very same animals without having to incorporate data from external sources, which may not necessarily reflect drug effects in the current study's animals.

· To address this comment and to clarify the source of the markers used to assess side-effects, we have revised the main text (lines 281-284) as follows: "In order to study the association between non-restorative transcriptomic shifts and drug side-effects, we looked for signs of unfavorable side-effects in the set of 26 physiological markers measured in the study animals (the same ones that had been analyzed throughout this paper, Supplementary Table 1)". We hope that this makes it clear that the data analyzed to detect side-effects is the same corpus of data that was analyzed throughout the manuscript, and includes all the physiological data we had available for the study animals. In addition, we now acknowledge and discuss the limitations that considering only a set of predefined biomarkers imposed on our study (lines 355-362).

4. How does the method of transforming gene expression profiles with PCA compare to Gene Set Enrichment Analysis that has been used in most previous manuscripts (eg: Dudley et al)? In GSEA the differentially expressed genes from the treatment are compared to the gene expression profile of the disease state to find anti-correlation and result in one clear score. Is there a reason to not use an established method like GSEA?

· We have now added an analysis that compares the metric by which we quantify transcritomic disease-reversal (TDI) to the GSEA-based score used by previous studies (page 6 in the main text lines 178-185, Supplementary Results 4.1 and Supplementary Figure 3). We show that the two scores are strongly correlated, as expected. Therefore, the ranking that GSEA-based methods would have assigned to the animals in this study according to the success of their respective treatments to reverse the disease transcriptomic patterns is similar to that assigned according to TDIs. We chose to retain the use of TDIs as it allows a simple decomposition of the treatment effect into the two orthogonal components (Figure 6a) that form the basis for the side-effect analysis (Figure 6b-d).

Also, can the choice of 200 most differentially expressed genes be justified? Is the method robust to taking a different number of genes?

· It was noted in the manuscript that the method is robust to this choice, but this might not have been conspicuous enough. We moved this note from its previous place to follow directly the sentence in which this choice is presented, which now reads as follows (lines 409-411): "The top $N = 200$ differentially expressed probes between the untreated 16-weeks HFD group and the LFD group were selected (we verified that our results were not sensitive to the choice of N within an order of

magnitude)".

5. What is the variation between mice? It would seem the variation is significant in Figure 2 b. Based on differences in gene-expression profiles, would different mice benefit from different drug-treatments? Can this be predicted from the data?

· It is true that there is considerable variation in the intra-group outcomes observed in the experiment, both with respect to transcriptome and physiology. Moreover, the animals that experienced favorable physiological outcomes are also the ones in which the drug was most successful in reversing the disease transcriptomic signatures (Figures 3, 5). This indeed suggests that different animals will benefit from different drugs. Predicting this is definitely worthwhile and intriguing, but we feel that the dataset we analyze in the current manuscript is not suitable for this purpose because it has only 8 animals in each treatment group.

6. In order to translate the results into treatment practice, would we now need to take samples from patients for eg liver gene expression profiling to determine the optimal treatment strategy?

· Indeed, performing tissue biopsies in patients to determine optimal treatment is not a viable option in most disorders. Rather, we think that the potential impact of our results lies not in prioritizing treatments in personalized medicine, but rather in calling attention and substantiating a conceptually new approach for guiding drug development and repurposing (Dudley *et al*, 2011; Sirota *et al*, 2011; Iorio *et al*, 2013; Pacini *et al*, 2013). This approach emphasizes the potential benefit of drugs that act to globally reverse the disease molecular signatures at relevant tissues, rather than aiming to remedy local aberrations, e.g. in specific enzymes, which is currently the scope of many extant treatments. This point is now addressed in more detail in the manuscript's summary (lines 380-391).

Minor comments: 1. The authors use 'lesser side-effects' in several places. I am sure they mean 'less side-effects' as in Figure 2 "the number of side-effects associated with treatment" is reported. As it is written now, they have less important side-effects rather than just less.

· Corrected. We thank the reviewer for noting the error.

References

American Diabetes Association (2013) Standards of Medical Care in Diabetes—2013. *Diabetes Care* **36:** S11–S66

Awtry EH & Loscalzo J (2000) Aspirin. *Circ.* **101 :** 1206–1218

Baron SH (1982) Salicylates as hypoglycemic agents. *Diabetes Care* **5:** 64–71

Campbell C, Smyth S, Montalescot G & SR S (2007) Aspirin dose for the prevention of cardiovascular disease: A systematic review. *JAMA* **297:** 2018–2024

Van Diepen JA, Berbée JFP, Havekes LM & Rensen PCN (2013) Interactions between inflammation and lipid metabolism: Relevance for efficacy of anti-inflammatory drugs in the treatment of atherosclerosis. *Atherosclerosis* **228:** 306–315

Dudley JT, Sirota M, Shenoy M, Pai RK, Roedder S, Chiang AP, Morgan AA, Sarwal MM, Pasricha PJ & Butte AJ (2011) Computational Repositioning of the Anticonvulsant Topiramate for Inflammatory Bowel Disease. *Sci. Transl. Med.* **3 :** 96ra76–96ra76

Enroth S, Johansson Å, Enroth SB & Gyllensten U (2014) Strong effects of genetic and lifestyle factors on biomarker variation and use of personalized cutoffs. *Nat Commun* **5:**

Furst DE, Ulrich RW & Prakash S (2012) Nonsteroidal Anti-Inflammatory Drugs, Disease-Modifying Antirheumatic Drugs, Nonopioid Analgesics, & Drugs Used in Gout. In *Basic & Clinical Pharmacology (12th ed.)*, Katzung BG Masters SB & Trevor AJ (eds) New York, NY: McGraw-Hill

Gao Z, Zuberi A, Quon MJ, Dong Z & Ye J (2003) Aspirin Inhibits Serine Phosphorylation of Insulin Receptor Substrate 1 in Tumor Necrosis Factor-treated Cells through Targeting Multiple Serine Kinases. *J. Biol. Chem.* **278 :** 24944–24950

Getz GS & Reardon CA (2012) Animal Models of Atherosclerosis. *Arterioscler. Thromb. Vasc. Biol.* **32 :** 1104–1115

Gilgore SG (1960) The Influence of Salicylate on Hyperglycemia. *Diabetes* **9 :** 392–393

Gilgore SG & Rupp JJ (1962) The long-term response of diabetes mellitus to salicylate therapy:

Report of a case. *JAMA* **180:** 65–66
Goldfine AB, Buck JS, Desouza C, Fonseca V, Chen Y-DI, Shoelson SE, Jablonski KA, Creager MA & Team for the T-F (Targeting IUS in T 2 D-MDAS (2013) Targeting Inflammation Using Salsalate in Patients With Type 2 Diabetes: Effects on Flow-Mediated Dilation (TINSAL-FMD). *Diabetes Care* **36 :** 4132–4139
Gower JC (1966) Some distance properties of latent root and vector methods used in multivariate analysis. *Biometrika* **53:** 325–338
Hansson GK & Hermansson A (2011) The immune system in atherosclerosis. *Nat. Immunol.* **12:** 204–212
Huang PL (2009) A comprehensive definition for metabolic syndrome. *Dis. Model. Mech.* **2 :** 231–237
Hundal RS, Petersen KF, Mayerson AB, Randhawa PS, Inzucchi S, Shoelson SE & Shulman GI (2002) Mechanism by which high-dose aspirin improves glucose metabolism in type 2 diabetes. *J. Clin. Invest.* **109:** 1321–1326
Hye A, Riddoch-Contreras J, Baird AL, Ashton NJ, Bazenet C, Leung R, Westman E, Simmons A, Dobson R, Sattlecker M, Lupton M, Lunnon K, Keohane A, Ward M, Pike I, Zucht HD, Pepin D, Zheng W, Tunnicliffe A, Richardson J, et al (2014) Plasma proteins predict conversion to dementia from prodromal disease. *Alzheimer's Dement.*
Iorio F, Bosotti R, Scacheri E, Belcastro V, Mithbaokar P, Ferriero R, Murino L, Tagliaferri R, Brunetti-Pierri N, Isacchi A & di Bernardo D (2010) Discovery of drug mode of action and drug repositioning from transcriptional responses. *Proc. Natl. Acad. Sci.* **107 :** 14621–14626
Iorio F, Rittman T, Ge H, Menden M & Saez-Rodriguez J (2013) Transcriptional data: a new gateway to drug repositioning? *Drug Discov. Today* **18:** 350–357
Jaichander P, Selvarajan K, Garelnabi M & Parthasarathy S (2008) Induction of paraoxonase 1 and apolipoprotein A-I gene expression by aspirin. *J. Lipid Res.* **49 :** 2142–2148
Kappus MS, Murphy AJ, Abramowicz S, Ntonga V, Welch CL, Tall AR & Westerterp M (2014) Activation of Liver X Receptor Decreases Atherosclerosis in Ldlr−/− Mice in the Absence of ATP-Binding Cassette Transporters A1 and G1 in Myeloid Cells. *Arterioscler. Thromb. Vasc. Biol.* **34 :** 279–284
Lamb J, Crawford ED, Peck D, Modell JW, Blat IC, Wrobel MJ, Lerner J, Brunet J-P, Subramanian A, Ross KN, Reich M, Hieronymus H, Wei G, Armstrong SA, Haggarty SJ, Clemons PA, Wei R, Carr SA, Lander ES & Golub TR (2006) The Connectivity Map: Using Gene-Expression Signatures to Connect Small Molecules, Genes, and Disease. *Sci.* **313 :** 1929–1935
Levin N, Bischoff ED, Daige CL, Thomas D, Vu CT, Heyman RA, Tangirala RK & Schulman IG (2005) Macrophage Liver X Receptor Is Required for Antiatherogenic Activity of LXR Agonists. *Arterioscler. Thromb. Vasc. Biol.* **25:** 135–142
Libby P, Ridker PM & Maseri A (2002) Inflammation and Atherosclerosis. *Circ.* **105 :** 1135–1143
Lumeng CN & Saltiel AR (2011) Inflammatory links between obesity and metabolic disease. *J. Clin. Invest.* **121:** 2111–2117
Pacini C, Iorio F, Gonçalves E, Iskar M, Klabunde T, Bork P & Saez-Rodriguez J (2013) DvD: An R/Cytoscape pipeline for drug repurposing using public repositories of gene expression data. *Bioinformatics* **29:** 132–134
Radonjic M, Wielinga PY, Wopereis S, Kelder T, Goelela VS, Verschuren L, Toet K, van Duyvenvoorde W, van der Werff van der Vat B, Stroeve JHM, Cnubben N, Kooistra T, van Ommen B & Kleemann R (2013) Differential Effects of Drug Interventions and Dietary Lifestyle in Developing Type 2 Diabetes and Complications: A Systems Biology Analysis in LDLr−/− Mice. *PLoS One* **8:** e56122
Ridker PM, Cushman M, Stampfer MJ, Tracy RP & Hennekens CH (1997) Inflammation, Aspirin, and the Risk of Cardiovascular Disease in Apparently Healthy Men. *N. Engl. J. Med.* **336:** 973–979
Ross R (1999) Atherosclerosis — An Inflammatory Disease. *N. Engl. J. Med.* **340:** 115–126
Shoelson S (2002) JMM – Past and Present. Invited comment on W. Ebstein: On the therapy of diabetes mellitus, in particular on the application of sodium salicylate. *J. Mol. Med.* **80:** 618–619
Shoelson SE, Lee J & Goldfine AB (2006) Inflammation and insulin resistance. *J. Clin. Invest.* **116:** 1793–1801
Sirota M, Dudley JT, Kim J, Chiang AP, Morgan AA, Sweet-Cordero A, Sage J & Butte AJ (2011) Discovery and Preclinical Validation of Drug Indications Using Compendia of Public Gene Expression Data. *Sci. Transl. Med.* **3 :** 96ra77–96ra77
Weber C & Noels H (2011) Atherosclerosis: current pathogenesis and therapeutic options. *Nat. Med.* **17:** 1410–1422
Weinberger KQ & Saul LK (2009) Distance Metric Learning for Large Margin Nearest Neighbor

Classification. *J. Mach. Learn. Res.* **10:** 207–244
Williamson RT (1901) On the Treatment of Glycosuria and Diabetes Mellitus with Sodium Salicylate. *BMJ* **1:** 760–762
Zadelaar S, Kleemann R, Verschuren L, de Vries-Van der Weij J, van der Hoorn J, Princen HM & Kooistra T (2007) Mouse Models for Atherosclerosis and Pharmaceutical Modifiers. *Arterioscler. Thromb. Vasc. Biol.* **27 :** 1706–1721

2nd Editorial Decision 01 December 2014

Thank you again for submitting your work to Molecular Systems Biology. We have now finally heard back from the two referees who accepted to evaluate the revised study. As you will see, the referees are now cautiously supportive. They still raise however several points that we would ask you to carefully address in a revision of the present work. In particular, Reviewer #4 notes that the conclusions about side-effects are not strong. While you show that non-restorative drugs have side-effects that are detectable with the available data, it seems to be difficult to claim that restorative drugs have *less* side-effects, since side effects relevant to this set of drugs were actually not tested. We would thus kindly ask the following:

- please condense the paper to the short Report format. We do not to be too strict about the character count but the number of key figures could be reduced. For example, Figure 1 could be transformed into Box 1 explaining the experimental design, and fig 2-5 could be kept. Figure 6 on side effects seems rather tentative in view of Reviewer #4 comments.
- The conclusions with regard to side-effects should thus be considerably toned down and removed from the title and abstract. Accordingly, the sections in Results and Discussion devoted to side-effects should also be condensed.
- To make the analysis more transparent, a table listing the known side-effects (from package inserts) for each drug should be provided and those that could be tested/estimated with the current measurements should be highlighted in this table with the resulting outcome based on the urine/blood measurements.
- Please re-test the code submitted, as Reviewer #2 seems to have encountered problems.
- As alternative title we would suggest: "Drugs that reverse disease transcriptomic signatures are more effective in a mouse model of dyslipidemia"
- When you resubmit your manuscript, please download our CHECKLIST (<http://msb.embopress.org/sites/default/files/additional-assets/EMBO%20Press%20Author%20Checklist%20-MSB.xlsx>) and include the completed form in your submission.

Thank you for submitting this paper to Molecular Systems Biology.

REFEREE REPORTS

Reviewer #2:

In this revised version, the authors have address adequately the points that we raised, as well as those of the other reviewers.

Two remaining minor points:

The code provided (which we applaud the authors for making available) does not seem to work. In addition, some documentation/manual should be provided, even if succinct, beyond the existing read me file.

As stated in our first review, we are convinced even if for the purpose of visualisation only, a more advanced dimensionaility reduction technique would be preferable beyond PCA (as authors themselves acknowledge in their response , the 2 PCAs used only capture part of the variability and hence the PCA plots do not show the actual clusters).

All in all, we consider this a nice piece of work. The analysis is relatively simple and based on published data, but conveys the message convincingly. This message, supporting the signature-

reversion paradigm, is of relevance for drug repositioning and discovery, and timely.

Reviewer #4:

The authors have addressed some but not all of the concerns raised by myself and the other reviewers. And I feel they are still making a shortcut that is not warranted.

I agree that this manuscript gives support to drug-repositioning by anti-correlated gene expression profiles. They do this by showing that indeed the drugs that are prescribed in this one particular disease revert gene expression profiles towards the healthy state.

Furthermore they claim that "non- restorative alterations to the gene expression are associated with drug side-effects". I can also agree with this, those side-effects that are reflected in the physiological paramaters are observed less in the treatments that revert the gene expression profiles better to the healthy state.

BUT there are many other side-effects not reflected in those physiological paramaters measured from blood and urine samples. This was raised by myself and other reviewers and no action was taken to address this point. Thus the authors cannot make a statement about those side-effects. The part of the title "and have fewer side-effects" should therefore be removed. All places where the authors claim fewer side-effects, should also be removed. That is, there are fewer side-effects for which blood- and urine-biomarkers are available. "Ulcer" and "Haematemesis" (gastrointestinal bleeding) being just two examples of side-effects that have not been investigated at all.

2nd Revision - authors' response 18 January 2015

Reviewer #2: In this revised version, the authors have address adequately the points that we raised, as well as those of the other reviewers. Two remaining minor points: # The code provided (which we applaud the authors for making available) does not seem to work. In addition, some documentation/manual should be provided, even if succinct, beyond the existing read me file.

· The constant RELOAD_DATA in the main script (DoMainAnalysis.m) determines whether to reload the raw data or to read preexisting matlab files that were previously loaded to save time. The first run of the code should choose the former option (in order to produce the matlab files), yet the code that we uploaded had a false value in that constant by default. We have now rectified this error.

· In addition, we now make it more explicit in the documentation that the code includes one function stub that should be implemented by the user to read the data table in the format that is particular to his or her study.

· We extended the documentation and included a reference manual (reference_manual.pdf) that describes the purpose of all functions and the analysis pipeline implemented by the main script.

As stated in our first review, we are convinced even if for the purpose of visualisation only, a more advanced dimensionaility reduction technique would be preferable beyond PCA (as authors themselves acknowledge in their response , the 2 PCAs used only capture part of the variability and hence the PCA plots do not show the actual clusters).

· We accept this point and now acknowledge in the discussion (page 11, 2nd paragraph) that using more sophisticated methods of dimensionality reduction may prove illuminating in future studies.

All in all, we consider this a nice piece of work. The analysis is relatively simple and based on published data, but conveys the message convincingly. This message, supporting the signature-reversion paradigm, is of relevance for drug repositioning and discovery, and timely.

· We thank the reviewers for their positive appreciation and for their comments that were instrumental in improving the manuscript.

© European Molecular Biology Organization

Reviewer #4: The authors have addressed some but not all of the concerns raised by myself and the other reviewers. And I feel they are still making a shortcut that is not warranted. I agree that this manuscript gives support to drug-repositioning by anti-correlated gene expression profiles. They do this by showing that indeed the drugs that are prescribed in this one particular disease revert gene expression profiles towards the healthy state. Furthermore they claim that "non- restorative alterations to the gene expression are associated with drug side-effects". I can also agree with this, those side-effects that are reflected in the physiological paramaters are observed less in the treatments that revert the gene expression profiles better to the healthy state. BUT there are many other side-effects not reflected in those physiological paramaters measured from blood and urine samples. This was raised by myself and other reviewers and no action was taken to address this point. Thus the authors cannot make a statement about those side-effects. The part of the title "and have fewer side-effects" should therefore be removed. All places where the authors claim fewer side-effects, should also be removed. That is, there are fewer side-effects for which blood- and urine-biomarkers are available. "Ulcer" and "Haematemesis" (gastrointestinal bleeding) being just two examples of side-effects that have not been investigated at all.

· We thank the reviewer for making this important distinction and corrected the manuscript accordingly. The manuscript no longer claims that drugs that induce large non-restorative transcriptomic alterations are associated with fewer side-effects. Instead, it makes the more modest claims that in the current dataset the drugs that had these effects were associated with unfavorable physiological outcomes. We explicitly acknowledge that the data available in the study animals allows observing only limited aspects of their physiology, and is thus unsuitable for a fully-developed study of adverse drug side-effects. We now only state in the discussion that we hypothesize that non-restorative transcritptomic alterations are associated with adverse side-effects, but that this hypothesis cannot be studied in the current data

© European Molecular Biology Organization
